# Evidence-based informed consent forms for total knee arthroplasty and anaesthesia: development and pilot study

**DOI:** 10.1186/s13018-026-06729-z

**Published:** 2026-02-05

**Authors:** Sandro Zacher, Julia Lauberger, Deha Murat Ates, Holger Bäthis, Andreas Böhmer, Stefanie Bühn, Anke Kaulbert, Tim Mathes, Sarah Oerder, Henning Rosenau, Anke Steckelberg, Felicia Steffen, Alina Weise, Julia Lühnen

**Affiliations:** 1 Institute of Health, Midwifery and Nursing Science, Medical Faculty of Martin Luther University Halle-Wittenberg, University Medicine Halle, Magdeburger Straße 8, 06112 Halle (Saale), Germany; 2 Department of Trauma and Orthopedic Surgery, University of Witten/Herdecke, Cologne-Merheim Medical Center, Ostmerheimer Str. 200, 51109 Cologne, Germany; 3 Department of Anaesthesiology and Intensive Care Medicine, Cologne-Merheim Medical Center, University of Witten-Herdecke, Ostmerheimer Straße 200, 51109 Cologne, Germany; 4 Faculty of Health-School of Medicine, Institute for Research in Operative Medicine, University of Witten/Herdecke, Ostmerheimer Str. 200, Building 38, 51109 Cologne, Germany; 5 Institute for Medical Statistics, University Medical Center Goettingen, Humboldtallee 32, 37073 Göttingen, Germany; 6 Interdisciplinary Scientific Center for Medicin, Ethics and Law, Martin Luther University Halle-Wittenberg, Universitätsplatz 5, 06108 Halle (Saale), Germany; 7 Institute of Clinical Nursing Science, Charité - Universitätsmedizin Berlin, Universität Berlin and Humboldt Universität zu Berlin, Charitéplatz 1, 10117 Berlin, Germany

**Keywords:** Osteoarthritis, Arthroplasty MeSH-Term, Consent forms, Consent documents, Risk communication, Informed consent form, Patient education

## Abstract

**Background:**

Informed consent is a legal and ethical prerequisite for elective procedures such as total knee arthroplasty (TKA). However, standard informed consent forms often lack evidence-based content and do not adequately support informed decision-making. The aim was to develop and pilot test an evidence-based informed consent form for TKA.

**Methods:**

We conducted a two-phase study. In Phase 1, we developed an extended, evidence-based informed consent form for TKA, as well as a corresponding form for anaesthesia, based on systematic evidence syntheses, expert reviews, and standards outlined in the *Guideline Evidence-based Health Information*. We also developed an e-learning program to train clinicians in the appropriate use of informed consent forms. In addition, we developed a multiple-choice test to assess patients’ knowledge and risk perception. In Phase 2, we piloted the informed consent forms with potential patients through think-aloud interviews and focus groups, assessing their acceptability, usability and comprehensibility with iterative revisions. The multiple-choice test was also piloted and revised. Qualitative data were analysed using qualitative content analysis.

**Results:**

We developed evidence-based informed consent forms for TKA and anaesthesia. Content and risk communication follow the *Guideline Evidence-based Health Information* and meet legal requirements under German law. Benefits and complications were presented using natural frequencies and visualised with bar charts. The forms were piloted in six think-aloud and three focus group interviews with 17 participants. Feedback from participants and experts highlighted the need for revisions in the presentation of numerical data, terminology, structure and layout, which were addressed iteratively. Overall, the forms were rated as understandable, relevant and helpful, though individual information needs varied. The 12-item multiple-choice knowledge test was revised to improve clarity and was perceived as comprehensible and applicable. The e-learning programme includes videos, texts and interactive elements, and is designed for flexible use over 90 to 180 min.

**Conclusions:**

The informed consent forms are now available. Evidence-based informed consent forms are feasible and perceived by patients as helpful and understandable. The variety of patients’ information needs underlines the need for personalised counselling and structural adjustments in clinical practice so that the potential of evidence-based informed consent forms can be exploited.

**Supplementary Information:**

The online version contains supplementary material available at 10.1186/s13018-026-06729-z.

## Background

Any invasive medical procedure requires the provision of information in advance and informed consent must be obtained. Otherwise, the measure legally constitutes an assault [1]. The human right to self-determination and the ethical principle of autonomy justify the principle of informed consent. This is also enshrined in legal provisions, which vary between countries [2–4].

In Germany, an informed consent discussion held with the patient is mandatory. A doctor is required to verbally provide information on the nature, importance, and scope of the intervention [5]. In practice, the discussion is usually completed on a standardised informed consent form [5].

For the English-speaking countries, a shift away from medical paternalism towards a more patient-centred approach has long been noted [4]. In Germany, patients’ rights have been codified in the German Civil Code (Sections 630a-630 h BGB) in 2013. However, Shah et al. raised concerns. They reported that out of the three acceptable legal approaches of informed consent, many states choose the reasonable patient standard: “What would the average patient need to know to be an informed participant in the discussion?” rather than the subjective standard: “What would this patient need to know and understand to make an informed decision?” [6].

Many sources mention what information should be provided or that the goal should be informed decision-making. They explicitly state that informed consent is about more than the patient’s signature. For example, the European Convention of Human Rights and Biomedicine states that information has to be given that also includes consequences and risks of the intervention and that helps patients take decisions [7]. Queensland Health for example emphasizes that informed consent comprises the entire process that aims to ensure that the patient fully understands the proposed health care and is supported to make an informed decision [8].

However, despite these developments, studies have still shown substantial shortcomings of informed consent forms. The analysis of German informed consent forms showed that the evaluation of the information in terms of topicality and reliability is only possible to a limited degree, and that weighing up different treatment options is not supported by a numerical description of benefits and harms [9]. To realise the demand for informed decision-making, new standards based on the criteria for evidence-based information are required [10].

One example of an elective procedure where the provision of evidence-based information and informed decision-making seems particularly relevant is knee replacement surgery for osteoarthritis. Osteoarthritis of the knee (OAK) reduces the quality of life of affected persons as osteoarthritis is associated with pain, loss of function and disability [11]. In Germany, the 12-month prevalence of OAK was 21.6% among women and 12.4% among men, assessed between 2019 and 2020 [12]. OAK is therefore highly relevant to the German healthcare system, as in addition to the direct costs of outpatient or inpatient treatment, the indirect costs of incapacity to work and early retirement due to OAK also have an economic impact [13].

Total knee arthroplasty (TKA) has the potential for improvements in pain, daily function and quality of life [14, 15] but should be prolonged for as long as possible. According to the German consensus-based guideline for the indication of TKA [16], conservative treatments such as physiotherapy or weight reduction, should be exhausted first. Nevertheless, with more than 200 surgical treatments per 100,000 persons TKA is frequently used in Germany [14]. However, a German study on second opinion showed that only 40% of recommended TKAs (*n* = 141) were confirmed by a second orthopaedist at an arthroplasty centre. It also showed that conservative treatments were not implemented consistently beforehand [17]. In addition, there are several studies on satisfaction after the surgery, with heterogeneous results [18, 19]. A systematic review displays that about 20% of patients report unsatisfactory pain reduction [20]. A report on TKAs in Germany indicates that only 43% of patients undergoing TKA are satisfied with the post-operative result [21]. Possible reasons for dissatisfaction could be, for example, unrealistic expectations regarding the outcomes of TKA, lack of knowledge about possible complications, as well as consultations with the physicians, which focused on clinical data only but not on the individual needs and preferences of the patients [22–25].

In preparation for the development of the evidence-based informed consent forms, we explored the decision-making processes as well as the information needs and preferences of patients. Interviews were conducted with patients and doctors [26] and an analysis of posts in Facebook groups was carried out [27]. The need for information is influenced by various factors, such as the extent of the symptoms, confidence in the doctor’s recommendation and whether or not the decision to undergo TKA has already been made [26]. Overall, the explorations show the need for information on the clinical picture of OAK, about how to deal with the diagnosis and with limitations in activities of daily living, and what this would mean for their future lifestyle. Patients reported information needs on different treatment options. Regarding conservative therapy they need an overview of possible interventions as well as more specific information about single options and their risks and benefits. Information needs for TKA include questions on indication and contraindication, the organisation and technical process (e.g., operating orthopaedist, surgical procedures), complications and risks (e.g., general risks and frequency of complications, frequency of failure to improve symptoms after surgery, time until revision surgery is necessary), prehabiltation, and on the course after surgery (e.g., expected pain intensity, restrictions in everyday and leisure activities, and long term improvement in quality of life) [26, 27]. With regard to anaesthesia, the interviews revealed the need for information on the possible anaesthetic procedures, sedation under spinal anaesthesia, on general risks and complications as well as on individually experienced complications in the past, and on the course after anaesthesia (e.g., expected restrictions, pain therapy) [26].

The aim of our study was to develop evidence-based informed consent forms for elective TKA and anaesthesia and to test their feasibility. In addition, the aim was to prepare material to test the informed consent form in a pilot interrupted time series study [28]. This included the development and cognitive pre-testing of a knowledge test and the development of a training programme for the use of the evidence-based informed consent forms.

## Methods

**Fig. 1:**
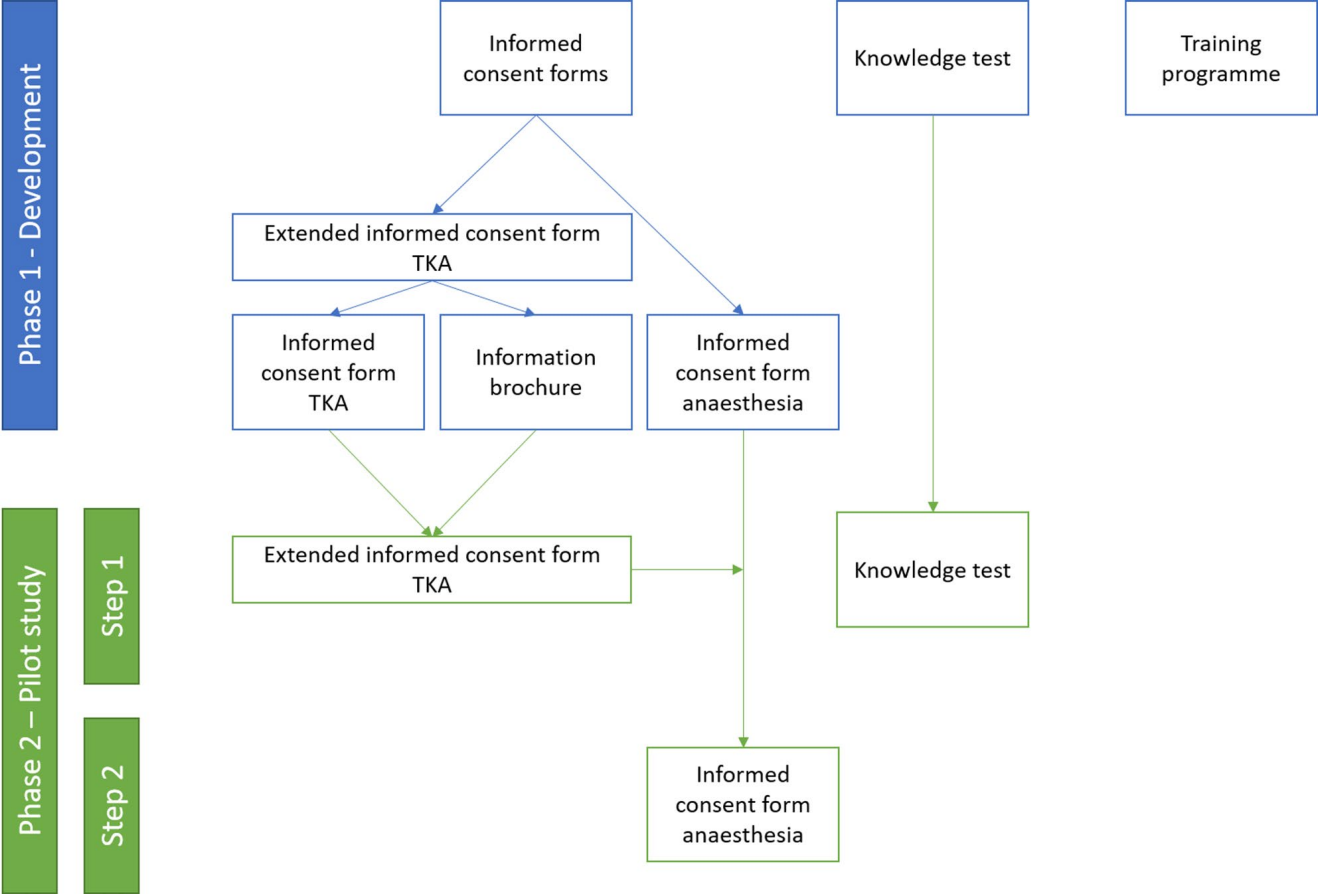
Study procedure

The study followed the Medical Research Council framework for the development and evaluation of complex interventions [29]. It was conducted in two phases (Fig. 1) and involved the development and piloting of multiple intervention components: evidence-based informed consent forms and a training programme and the development of a knowledge test.

Phase 1 included the development of the informed consent forms for TKA and anaesthesia, together with a knowledge test to assess risk perception and gist and verbatim knowledge, and a training programme for doctors to support the implementation of the informed consent forms. Due to the scope of the informed consent form for TKA, two documents were developed: the informed consent form itself and an information brochure. As we consider both documents to be a necessary unit, we refer to both documents as the extended informed consent form for TKA. If only one of the two documents is involved, we will refer to it separately.

In phase 2, we pilot tested these materials. The feasibility of the informed consent forms was assessed in a two-step process: first, the extended informed consent form for TKA was piloted, and based on these findings, the informed consent form for anaesthesia was finalised and also piloted. The knowledge test was cognitively pre-tested in this second phase.

The development and piloting of the informed consent forms are reported according to the revised criteria for reporting the development and evaluation of complex interventions in healthcare (CReDECI 2) [30], while the qualitative methods are reported according to the consolidated criteria for reporting qualitative research (COREQ) [31] to ensure transparency and comprehensiveness.

### Deviations from the study protocol

It was planned that data extraction in the systematic literature searches would be carried out independently by two people. In addition, the quality of the evidence was intended to be assessed using the GRADE approach [32]. However, due to the unexpectedly large number of individual studies and outcome measures, a deviation was made: data extraction was performed by one person and checked by a second. In addition, the quality of the evidence was not assessed.

### Development of the informed consent forms

Following the recommendations of the *Guideline Evidence-based Health Information* when developing the informed consent forms [10], we considered the content and the presentation of the informed consent forms. Particular attention was paid to ensuring that the materials were suitable for our target audience of patients making decisions regarding TKA. This was achieved by using appropriate language, short sentences, clear structural organisation and a reader-friendly layout. The content was structured to address key elements such as the purpose of surgery, the natural progression of the condition, available treatment options, and patient-relevant outcomes, including potential benefits and harms. To generate quantitative information for the development of the informed consent forms, we conducted systematic evidence syntheses using Cochrane methods [33], comparing the effectiveness and potential harms of TKA and related anaesthetic procedures. The PICO questions were informed by the needs and preferences of patients, as determined through a systematic literature search and qualitative interviews [26], as well as the clinical expertise of two orthopaedic surgeons (HB, DMA) and an anaesthesiologist (AB). Additionally, standard informed consent forms served as a reference for the content.

Another core element of the content is the communication of transparency criteria. We followed the recommended standards, for example by clearly stating the authorship, the funding sources, the references used, the timeliness of the information and the methods used to develop the materials [10].

With regard to the appropriate presentation of content, we communicated the benefits and harms of procedures using absolute risk formats, natural frequencies and standardised reference parameters (denominators). We avoided verbal presentations of risks and applied gain and loss framing to ensure balanced risk communication. Wherever necessary, uncertainty, missing or insufficient evidence were disclosed [10].

#### Extended informed consent form for TKA

Systematic evidence syntheses were conducted to provide quantitative information on the harms and benefits of TKA and different alternatives. The pre-defined PICO questions, included comparisons of TKA with conservative management and partial knee arthroplasty, as well as comparisons of different prosthesis types, fixation techniques, surgical approaches and procedures (e.g. fast-track) (Table 1).

**Table 1:** PICO questions for systematic evidence syntheses on TKA

No.	PICO question
1	Is total knee arthroplasty more effective than conservative treatment methods in people with osteoarthritis of the knee in terms of pain reduction, functional improvement, quality of life, patient satisfaction, and complications?
2	Is total knee arthroplasty more effective than unicondylar arthroplasty in people with osteoarthritis of the knee in terms of pain reduction, functional limitation, quality of life, patient satisfaction, and complications?
3	Is a non-constrained knee arthroplasty more effective than a semi-constrained (posterior stabilized) knee arthroplasty in people with osteoarthritis of the knee in terms of pain reduction, functional limitation, improvement in quality of life, patient satisfaction, and complications?
4	Is total knee arthroplasty with patellar resurfacing more effective than knee arthroplasty without patellar resurfacing in people with osteoarthritis of the knee in terms of pain reduction, reduction of functional impairment, improvement in quality of life, patient satisfaction, and complications?
5	Is total knee arthroplasty with mobile bearing more effective than knee arthroplasty with fixed bearing in people with osteoarthritis of the knee in terms of pain reduction, reduction of functional impairment, improvement in quality of life, patient satisfaction, and complications?
6	Is a cemented knee arthroplasty more effective than a cementless knee arthroplasty in people with osteoarthritis of the knee in terms of pain reduction, functional limitation, quality of life, patient satisfaction, and complications?
7	Is computer-assisted navigation for total knee arthroplasty in people with osteoarthritis of the knee more effective than the standard procedure for total knee arthroplasty in terms of pain reduction, reduction of functional impairment, improvement in quality of life, patient satisfaction, and complications?
8	Is the use of patient-specific instruments for total knee arthroplasty in people with osteoarthritis of the knee more effective than the standard procedure in terms of pain reduction, functional limitation, quality of life, patient satisfaction, and complications?
9	Is minimally invasive total knee arthroplasty more effective than conventional total knee arthroplasty in people with osteoarthritis of the knee in terms of pain reduction, functional improvement, quality of life, patient satisfaction, and complications?
10	Is robot-assisted navigation for total knee arthroplasty in people with osteoarthritis of the knee more effective than the standard procedure for total knee arthroplasty in terms of pain reduction, reduction of functional limitations, improvement in quality of life, patient satisfaction, and complications?
11	Is the use of patient-specific prostheses for total knee arthroplasty in people with osteoarthritis of the knee more effective than the standard procedure for total knee arthroplasty in terms of pain reduction, reduction of functional limitations, improvement in quality of life, patient satisfaction, and complications?
12	Is a fast track procedure for total knee arthroplasty in people with osteoarthritis of the knee more effective than the standard procedure in terms of pain reduction, reduction of functional limitations, improvement in quality of life, patient satisfaction, and complications?

We searched MEDLINE (via PubMed), EMBASE (via Elsevier), the Cochrane Central Register of Controlled Trials (CENTRAL, via the Cochrane Library), and Epistemonikos. The primary search was conducted on 5 November 2020, with an update for systematic reviews on 30 March 2021. In addition, reference lists of included systematic reviews were screened for further eligible studies.

As a comprehensive search was conducted for all PICO questions, in a first step, titles and abstracts were independently screened by pairs of reviewers and assigned to the PICO questions. In the second step, full texts were independently assessed for eligibility by pairs of reviewers using PICO-specific criteria. Disagreements were resolved by discussion or by involving a third reviewer. The screening of titles and abstracts was supported by Rayyan [34]. The review of full texts and allocation of studies was managed in structured Excel spreadsheets. Additional file 1 contains the PICO questions, inclusion and exclusion criteria, and search strategies for each database.

Data extraction was carried out by one assessor and checked by a second. We used a standardised Excel form to extract the outcomes. Study and patient characteristics and intervention details were marked in the study texts and extracted if needed for metanalyses. Classification of complications was based on Knee Society definitions [35], and patient-reported outcome measures were classified into activities of daily living, sports, quality of life, and pain. Risk of bias was assessed independently by pairs of reviewers. We used the Cochrane Risk of Bias 2 tool for randomised controlled trials and the CASP checklists for cohort and registry studies. Conflicts were resolved by discussion and, if necessary, by a third reviewer.

Meta-analyses were performed for each comparison, e.g. TKA vs. partial knee replacement, when there were sufficient data and clinical and methodological homogeneity. We calculated risk ratios for binary outcomes and mean difference for continuous outcomes. Where necessary, missing standard deviations were calculated from median, quartiles, (optional ranges), and sample size according to the approach suggested by Wan et al. [36]. All patient-reported outcome scales were transformed into a unified scale from 0 to 100, with 100 always representing the best outcome, without impairment or symptoms. We used the modified Hartung-Knapp method with an ad-hoc correction for the 95%-CIs for pooling studies [37]. For meta-analyses including zero event studies, we used beta-binomial models [38]. Data synthesis and visualisation were performed using R Version 4 and SAS 9.4.

Different strategies were employed to present complications. If meta-analyses revealed statistically significant differences in the frequency of a complication between different surgical options, these differences were presented. Where no statistically significant differences were found in individual comparisons, the observed frequencies across all comparisons were summarised. The calculations were performed using a structured Excel spreadsheet. If no causal relationship could be established between TKA and the complication, no frequencies were presented.

#### Informed consent form for anaesthesia

While the general methods described for the TKA informed consent form were retained, some aspects were adapted for anaesthesia. PICO questions were developed to compare spinal versus general anaesthesia, intubation versus laryngeal mask for airway management, and the use of sedation with spinal anaesthesia versus no sedation (Table 2). In consultation with the clinical expert, the population inclusion criteria were expanded from the TKA informed consent form and adjusted for each comparison.

**Table 2:** PICO questions for systematic evidence syntheses on anaesthesia

No.	PICO question
1	Is spinal anaesthesia more effective than general anaesthesia in terms of perioperative and postoperative complications in people undergoing elective knee or hip replacement surgery?
2	Is ventilation using a laryngeal mask during general anaesthesia more effective in terms of perioperative and postoperative complications compared to intubation in people undergoing elective surgery?
3	Is neuraxial anaesthesia combined with sedation more effective in terms of perioperative and postoperative complications compared to neuraxial anaesthesia with placebo or a form of distraction?

The literature search was conducted between 22 October 2021 and 3 March 2022 in MEDLINE (via PubMed), EMBASE (via Elsevier), the Cochrane Central Register of Controlled Trials (CENTRAL, via the Cochrane Library) and Epistemonikos. Databases were selected according to the specific focus of each PICO. The presentation of quantitative information for the anaesthesia informed consent form was limited to patient-relevant complications in accordance with the National Anaesthesia Clinical Outcomes Registry (NACOR) classification [39]. See Additional File 1 for more details.

#### Expert reviews

##### Legal review

To ensure that the informed consent forms complied with legal requirements in Germany, the study team’s legal experts (HR and FS) conducted an extensive review of relevant case law and legal literature concerning medical consent and disclosure. The legal search was carried out using a snowball approach, beginning with annotated statutory texts and expanding through cited sources. Individual court rulings were analysed in detail, with particular attention to whether legal principles from other medical contexts could be transferred to TKA. The legal analysis focused especially on the applicability of sections 630d and 630e of the German Civil Code (BGB), supported by continuous literature review and guided by the *Guideline Evidence-based Health Information* [10].

##### Reviews of medical societies

A legal report was prepared and, together with the evidence-based informed consent forms for TKA and anaesthetic procedures, submitted to relevant specialist societies for review and feedback.

##### Reviews of experts for health information

To ensure that the informed consent forms were written in clear and understandable language, we consulted two experienced health information creators (IH and RB) prior to the pilot phase. They checked the informed consent forms for general readability and clarity, highlighting complex or ambiguous passages and suggesting alternative wording to improve comprehension for the target group.

### Piloting of the informed consent forms

A qualitative feasibility study was conducted to assess the acceptability, usability and comprehensibility of the informed consent forms. Piloting involved an iterative process of data collection, data analysis and revision.

#### Setting and sample

The aim was to test the informed consent forms with the potential target group. Therefore, we recruited participants aged > 18 with knee pain who had already had a TKA or were planning a TKA or were considering the option of a TKA. Characteristics such as age, level of education and experience with TKA aimed to be as versatile as possible. A multifaceted recruitment strategy was implemented to achieve the planned sample. Recruitment took place at an academic teaching hospital in a large city in western Germany, where the main study was also conducted, and a university hospital in eastern Germany. Patients who met the inclusion criteria were informed about the study by a doctor or study staff within the consultation. If patients were interested, they could leave their contact details and were then contacted by study staff with information about the study. Furthermore, recruitment took place online throughout Germany by posting in Ebay Classifieds and in Facebook groups about OAK and TKA. An information text about the study was posted with contact details. All participants were offered an incentive of 20 euros.

#### Procedure and data collection

The pilot testing comprised two steps. In the first step we tested the extended informed consent form for TKA.

The data was collected using think-aloud [40] and follow-up probing questions in individual interviews. The think-aloud procedure began with a standardised introduction, during which participants were asked to read through the materials and speak aloud their thoughts as they read. During the think-aloud phase, participants were not interrupted, but were reminded, if necessary, to think aloud and field notes were taken. Subsequently, questions were asked about the material based on predefined probing questions, and any remaining uncertainties were addressed. The probing questions and the standardised think-aloud introduction can be found in Additional file 2. The interviews were conducted online using the Webex video conferencing software and were audio recorded.

After initial revisions, semi-structured focus group interviews on the extended informed consent form for TKA were conducted (Additional file 3). The participants received the extended informed consent form one week before the focus group interview and were requested to read through these materials and take notes if necessary. The audio-recorded focus group interviews were conducted online using the Webex video conferencing software by two researchers; while one person moderated the discussions, the second person took field notes.

The second step comprised the testing of the informed consent form for anaesthesia. Results of steps one also informed the draft of this informed consent form and therefore, no think-aloud interviews were conducted. The piloting using focus group interviews was identical to the first step.

Two focus groups were planned for each of the informed consent forms.

All interviews were conducted by JLa and SZ, who have moderate experience in conducting interviews and were involved in the development of the materials. The interview guides were created based on experience from previous projects, but were not piloted before use. The transcripts were not returned to the participants for comments. Participants received information about the development of the materials and the involvement of the interviewers prior to the interviews. Baseline characteristics regarding age, sex, education level and experience with TKA were collected prior to all interviews.

#### Data analysis

In accordance with the iterative process of data collection, data analysis and revision, in a first step, the audio recordings of the think-aloud interviews and the associated field notes were analysed immediately after they were conducted regarding the need for revision. Revisions were implemented directly so that these could be piloted in subsequent think-aloud interviews. Think-aloud interviews were conducted until no new revision requirements could be identified. In a second step, the transcribed focus group interviews were analysed using Mayring’s qualitative content analysis [41]. Based on the objective of the piloting, a deductive category system was developed with the main categories of acceptance, usability and comprehensibility, which was used to structure the transcripts. This structure was then used to identify the need for revisions. All analyses were carried out by JLa and SZ, with one person checking the analyses of the second person. We used MAXQDA and Excel for analysis. After each focus group, the materials were analysed and, if necessary, revised. The baseline characteristics were analysed descriptively.

### Development and cognitive pre-test of a knowledge test

To assess knowledge and risk perception in the monocentric interrupted time series pilot study [28], a knowledge test was developed based on the evidence included in the informed consent forms. The knowledge test contains questions on gist and verbatim knowledge, which were created based on the information needs identified and the estimated relevance for decision-making. We chose a multiple choice format with one correct answer and four distractors and an open answer format for frequency information. Cognitive pre-tests were carried out to ensure the comprehensibility of the questions for the target group.

#### Setting and sample

The planned sample included people aged ≥ 18 years with OAK who had considered a TKA, refused a TKA or had already had a TKA. The recruitment was advertised via an announcement on Ebay classifieds. This included information about the purpose, scope, procedure, and a contact person. When potential participants contacted the study team, informed consent was obtained. Participants received an incentive of 10 euros.

#### Data collection

The data was collected online by SZ via the Webex video conferencing software using think-aloud [40] interviews followed by probing questions about the comprehensibility of the questions, answer options and technical terms. The interviews were audio-recorded and field notes were taken. We planned five to eight interviews.

#### Data analysis

The data was analysed by JLa and SZ in an iterative process of data collection, analysis and revision using field notes in which incomprehensible terms, uncertainties or incomprehensible passages were marked and subsequently revised.

### Development of the training programme for the informed consent forms

To increase the acceptability and applicability of the newly developed evidence-based informed consent forms, a training programme was developed for doctors involved in the informed consent discussion. The training programme was based on the Evidence-Based Medicine Network’s curriculum for evidence-based decision making, which had already been developed and piloted as a blended learning course for doctors and medical students [42]. Due to possible restrictions caused by the COVID-19 pandemic and the time and organisational requirements in the clinic, the training programme was developed as e-learning for the ILIAS learning management system. Learning objectives were formulated, existing content from the curriculum was adopted, and further piloted training elements [43] were integrated. As part of the training programme, individual pages of the informed consent forms were worked through step by step, and the content presented, and its development were explained using the methods of evidence-based medicine. Interactive navigation elements were used to access the information in different media formats (text, images, videos). A forum was set up for dialogue between participants and researchers. The learning programme was designed to be self-directed, so that participants could shape the learning process individually. In addition, individual knowledge questions from the blended learning training programme were used to design a final exam. Passing this exam enabled participants to receive continuing education credits.

## Results

The German version of the informed consent forms for TKA and anaesthesia are available (Additional file 4).

### Extended informed consent form for TKA

The systematic literature search identified 7,681 articles on TKA. Out of these, 103 were included in the preparation of the extended informed consent form. Details on the results of the systematic evidence syntheses are available upon request and in the German-language method report [44].

Due to the comprehensive content, two documents were prepared. Firstly, there is the informed consent form, which is structured similarly to the standard form and primarily covers the complications and legal aspect of consent. Secondly, there is the information brochure, which provides a detailed comparison of the benefits of the various options. We consider both documents to be essential and together they constitute the extended informed consent form for TKA. Table 3 shows the content of the informed consent form.

**Table 3:** Content of the informed consent form for TKA

Section	Content summary
Purpose of the informed consent form and definition of the target population	This section outlines the purpose of the informed consent form, which is to enable patients to make informed decisions, and clarifies which patients the document is intended for
Overview of OAK and its natural course	This section describes the anatomy of the knee and the general pathophysiology and natural course of OAK without treatment
Methods for the preparation of the informed consent form	This section explains how the material was developed. It includes an explanation of a randomised controlled trial (RCT) and the stochastic uncertainty and generalisability of the presented frequencies
Conservative treatment options	This section compares TKA with conservative treatment and discusses the respective benefits and complications. The presentation of frequencies is explained at the beginning of the section
Surgical treatment options for joint replacement	This section describes partial knee arthroplasty and TKA, as well as the different types of prostheses, fixation techniques and surgical approaches for TKA
Complications and adverse events of TKA	This section provides a detailed overview of the potential complications and adverse events that can occur during and after TKA. The presentation of frequencies is explained at the beginning of the section
Information on pre- and postoperative management	This section provides information on preparing for surgery and managing the postoperative period. This includes information on mobility restrictions, postoperative medication and rehabilitation
Links to additional sources	This section contains QR codes that lead to additional health information, the bibliography for the informed consent form and a detailed list of authors, as well as the complete method report
Patient health status questionnaire	This section contains the standardised questionnaire on the current state of health, which was derived from the standard informed consent form in consultation with clinical experts
Patient notes and comments on the planned operation	This section contains additional fields for patient notes and detailed documentation of planned surgical methods, such as the intended surgery and the surgical procedure
Legal consent	This section contains the formal written consent statement and a checkbox to confirm receipt of a copy of the informed consent form. Explicit confirmation of rejection of the procedure has been removed, as this is not legally required and could create the impression that the procedure is mandatory, despite it being elective
Imprint and transparency criteria	This section includes the imprint, details of how up to date the informed consent form is, when the next update is planned, and the funding source

The information brochure reiterates key elements from the informed consent form. The brochure’s main focus is to present the benefits of the various surgical options in more detail. It provides more information on the procedures that were only briefly mentioned in the informed consent form and includes visuals of the different surgical options. The core of the information brochure consists of tables that use numerical data to compare the benefits and differences in complications of the various surgical options. Additionally, it offers information on discharge preparation and recovery,

No study could be identified for the comparison of patient-specific prostheses with standard prostheses and is therefore not included in the extended informed consent form. No sufficient evidence could be found to compare fast-track procedure with the standard procedure. Therefore, we decided not to present the benefits and complications.

#### Presentation of complications

Meta-analyses revealed statistically significant differences in the frequency of complications in only one comparison. This related to the frequency of deep prosthetic infections in fixed-bearing versus mobile-bearing TKA. This information was included in the informed consent form at the beginning of the section on complications and the frequencies were compared using standardised bar charts. In the information brochure, this comparison was presented in the relevant section. For the other comparisons, it was stated that no statistically significant differences were found in the meta-analyses.

The next section of the informed consent form listed all the complications identified by the Knee Society with descriptions and potential associated actions. These descriptions were developed in collaboration with two clinical experts (HB, DMA). The frequencies of complications were provided where possible. An example of how this was implemented in the final version of the informed consent form is shown in the results of the pilot testing in Fig. 5 using the example of anaesthesia.

Frequencies were presented by reporting the minimum and maximum frequencies observed across all comparisons, regardless of statistical significance. These ranges were based on both meta-analysis results and individual study results. For meta-analyses, the number of events in the intervention group was calculated based on the effect size and the number of events in the control group. For individual studies, the absolute number of events in both the intervention and control groups was used. Frequencies were presented using a standardised bar chart expressing natural frequencies, standardised per 1,000 people. The number of studies and the total number of participants contributing to the data were also presented underneath the bar chart. Where individual studies deviated substantially from the overall findings without a clear methodological justification for exclusion, such cases were noted separately in text form below the chart. For example, a statement such as “A study with 100 participants reported a higher rate of blood transfusions” was included. Uncertainty was described verbally where appropriate, and in some cases no frequency was reported.

Only randomised controlled trials (RCTs) were used to present the frequency of complications. Cohort studies were not used because they did not provide results that were meaningfully different from the included RCTs. To describe the revision rates, registry data were included in addition to randomised trials comparing different surgical options. However, we did not report frequencies based on the registry data, but only described which techniques were associated with higher revision rates.

The last section on complications in the informed consent form described complications that were either listed in the standard informed consent form or reported in the literature but for which no clear causal link with TKA could be established. No frequency was reported.

#### Presentation of benefits

**Fig. 2:**
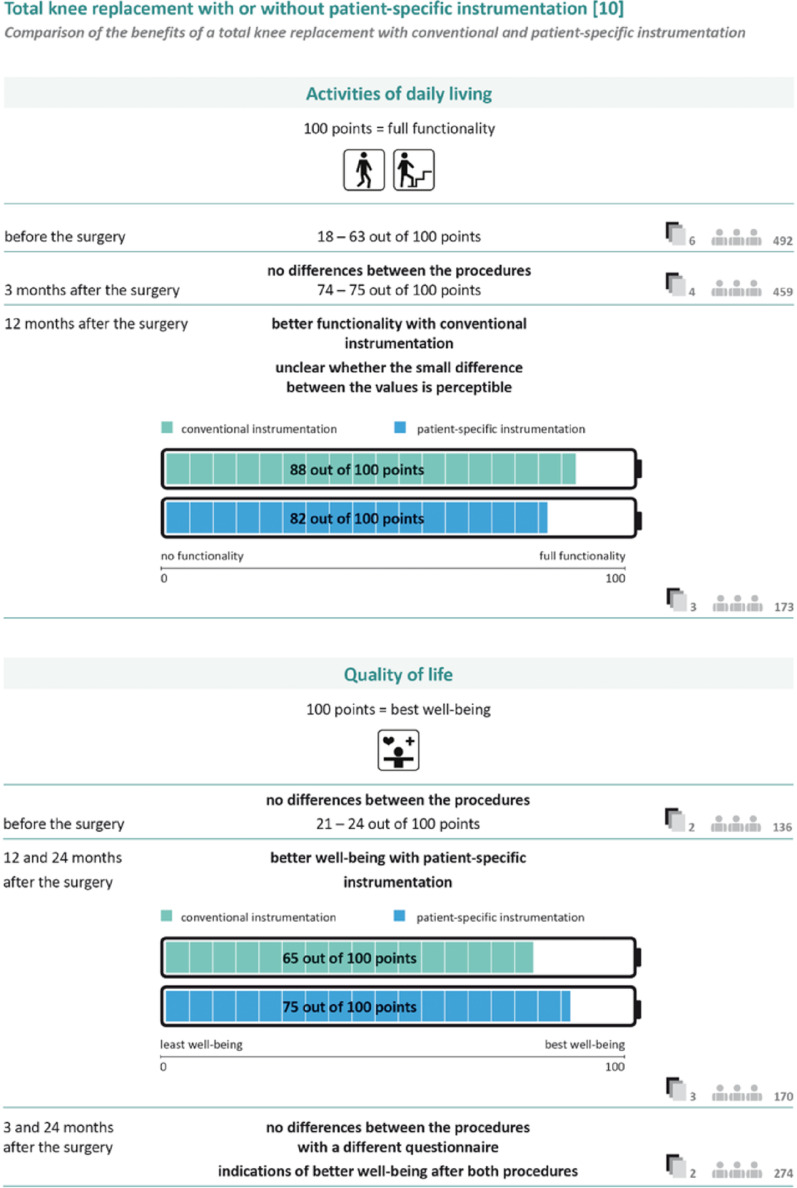
Revised representation of endpoints with and without difference between options

To illustrate the benefits, we selected validated patient-reported outcome measures (PROMs) (WOMAC, KSS, SF-36, VAS, OKS, KOOS, EQ-5D) [45–51] on different domains of life (activities of daily living, sport activities, pain and quality of life). The PROMS and subscales that could be combined due to similar or identical content were identified. All scales were converted to a scale of 0-100, where 100 represents full functioning, no pain or highest quality of life. Where the data allowed, meta-analyses were carried out and the differences at different points in time were presented in bar charts. The number of included studies and the number of study participants were reported with the bar charts. The presentation was divided into the different domains of life, represented by pictograms of activities measured with the PROM. Where possible, the subdomains of the PROMs were also reported. Where data were available, the minimum and maximum unweighted baseline values were presented, followed by the available results at different standardised time points (3 months, 6 months, 12 months and 24 + months). An example of how this was implemented in the final version of the informed consent form is shown in the results of the pilot in Fig. 2. If the results at different time points were identical, they were presented together. An algorithm was developed for the presentation. For meta-analyses with a statistically significant difference and clinical relevance, a clear verbal interpretation of the effect was provided and the weighted means were presented separately for the intervention and control groups using bar charts. For available meta-analyses with a statistically significant difference but without clinical relevance, the bar chart presentation was the same. The verbal interpretation indicated that it was unclear whether the difference between the values was noticeable. For meta-analyses with no statistically significant difference, a verbal description was provided and the range of weighted means across groups was presented in a single bar chart. If no meta-analyses were available, but a large individual study with low risk of bias was available for a relevant comparison, the same procedure was followed as for a meta-analysis. If no study results or only small studies were available, this was reported verbally.

### Informed consent form for anaesthesia

The systematic literature search identified 9,073 articles on anaesthesia. Of these, 64 were used to prepare the informed consent form.

The structure and the general content of the informed consent form is in large parts identical to the informed consent form for TKA. One difference is that the information on the development of the informed consent form, randomised controlled trials, stochastic uncertainty and the generalisability of numerical data to individual patients was shortened with a reference to the TKA material. In addition, a section on the design and interpretation of register studies has been added. Complications already described in the informed consent form for TKA were not listed in this document.

The presentation of complications followed the same procedure as described for TKA. One difference was that we also included data derived from non-randomised trials and registries due to a lack of relevant research. The limitations of the study designs were highlighted in the wording of the differences and in the description of the study design. Furthermore, the presentation was standardised to 10,000 people. No benefits were reported.

### Expert reviews

#### Legal review

Key findings of the legal review were summarised in internal briefing documents and during the development of the informed consent forms, the legal team worked closely with project partners to address specific questions, such as: the appropriate scope and depth of information provided; the inclusion and communication of rare complications and the necessity of including a field for non-consent. These consultations informed both the iterative refinement of the informed consent forms and the legal review process. The final evidence-based informed consent forms fulfil the legal requirements. Further insights into legal issues that arose, including the use and implementation of informed consent forms in the clinic, will be described in the publication on the accompanying process evaluation of the interrupted time series study.

#### Reviews of medical societies

Despite the submission of a comprehensive legal opinion confirming full compliance with all legal requirements, the specialist society that reviewed the informed consent form for TKA was sceptical about legal certainty. In addition, suggestions were made for changes to the content, some of which were adopted after consultation with the clinical expert, for example in the description of the knee joint. Other comments concerned the discussion of conservative treatment and criticism of the results on benefits and complications compared to TKA. Only minor changes were made here, but the comprehensive discussion of conservative treatment, including benefits and complications compared to TKA, was retained. A further comment concerned the scope and integration into the clinical routine. In terms of content, no changes could be made to shorten the scope without neglecting essential aspects of evidence-based health information. As the larger size of the informed consent form compared to the standard informed consent form was mainly due to the layout and the graphical representation of frequencies, we decided not to make any changes and to pilot this with patients. The specialist society that reviewed the informed consent form for anaesthetic procedures agreed with the content and accompanying legal opinion. No major changes were required.

#### Reviews of experts for health information

In response to the detailed feedback from the two experts for health information, we made several revisions. In particular, we simplified sentence structures and replaced long, compound constructions with shorter, more direct formulations. Selected terms that were considered too technical were replaced with more common alternatives. Section headings were changed to concise questions that reflect the questions patients might ask themselves when seeking information.

### Pilot study

Between December 2021 and May 2022, six think-aloud interviews and three focus group interviews (TKA 1 *n* = 4; TKA 2 *n* = 4; anaesthesia *n* = 3) were conducted with a total of 17 participants (Table 4). The think-aloud interviews lasted between 74 and 147 min, with a median duration of 89 min, while the focus group interviews lasted between 64 and 130 min, with a median duration of 130 min.

**Table 4:** Participants’ characteristics

	TKA (*n* = 13*)	Anaesthesia (*n* = 3)
Age, mean (R)	48 (33–59)	56 (48–60)
Women	8	0
Divers	0	0
Education		
Secondary school	1	2
Vocational training	6	0
University degree	6	1
Status		
TKA not yet scheduled	7	2
Scheduled for TKA	2	0
TKA carried out	3	1

*Data missing from one person; R = range

The piloting took place in an iterative process of interviews, analysis and revision. We used a deductive category system with three main categories and specific subcategories to identify areas for revision. The first category was “acceptance”, which included “relevance”. The second was “usability”, which included “structure” and “design”. The third was “comprehensibility”, which included “content understanding” and “text difficulty”. General minor revisions can be found in Table 5.

**Table 5:** Minor revisions

Barriers	Revision
The scope of the materials makes it difficult to gain an overview of the content and its relevance for the user	Some of the headings were reformulated as questions and a table of contents was created
Repetitive elements were found to be distracting: (1) references to stochastic uncertainty in each section where frequencies are reported, (2) references between materials to figures and numerical data.	(1) Information on stochastic uncertainty has been retained, as individual sections could be skipped if the materials are used individually. Notes have been shortened and their design adapted so that they take up less space (2) References between the materials have been reduced and placed at the beginning of a section
Sorting of the complications was considered confusing.	Complications with frequency data were sorted according to the difference between the procedures and the frequency of occurrence. Other complications and adverse events were sorted alphabetically
Texts contained unfamiliar or difficult terms and were generally perceived as too complex.	Unfamiliar terms were replaced or clarified and wording was simplified
The images of the knee are not labelled as to whether they are shown from the side or the front.	Labelling was carried out
General example of the RCT process was difficult to understand and led to confusion among users	The comparison between TKA and partial arthroplasty was chosen to explain an RCT
Expressed uncertainties about the frequency of complications led to insecurity, as the reason for the uncertainty is not clear.	The reasons for the uncertainty regarding the frequency were clarified directly in the complication or in the list of complications included at the beginning of the section

With regard to the acceptance of the materials, different aspects were identified. The relevance of individual content was rated subjectively and very differently. For example, some users rated the presentation of frequency data as extremely relevant, while others did not consider it relevant at all. The aim of the revision was to make it easier to adapt to individual user preferences (Table 5). There were also differences in expectations regarding the scope of the content and materials and their complexity. In the interviews, the high perceived relevance of individual content was partly contradictory to the expectation of reduced scope and lower complexity, and also contradictory to the requirements for evidence-based health information. The detailed graphical comparison of options with no difference was perceived as less relevant, which meant that the graphical presentation was reduced to endpoints with a difference between the options and all other comparisons were presented verbally and numerically (Fig. 2).

In terms of usability, there was a need for revision due to the conflict between individual information needs and the scope and complexity of the content. The division of the TKA content into the informed consent form and an information brochure created barriers in the structure, for example due to repetitions and references, which were perceived as irritating by the users. In addition, further barriers in the structure were identified and revised (Table 5).

Individual contents of the informed consent forms, such as the description of the target group of the materials by means of a diagnosis and the comparison of different surgical methods, require that patients have or receive information on their diagnoses and possible surgical methods. In the interviews, it was recognised that this information is sometimes not available, making it difficult to apply the materials to the patient’s individual situation, which sometimes led to uncertainty.

Regarding comprehensibility, barriers and facilitating factors were identified. Design decisions were identified as barriers, but revisions to the design also promoted comprehensibility. Overall, there are individual differences regarding the comprehensibility of individual elements.

**Fig. 3:**
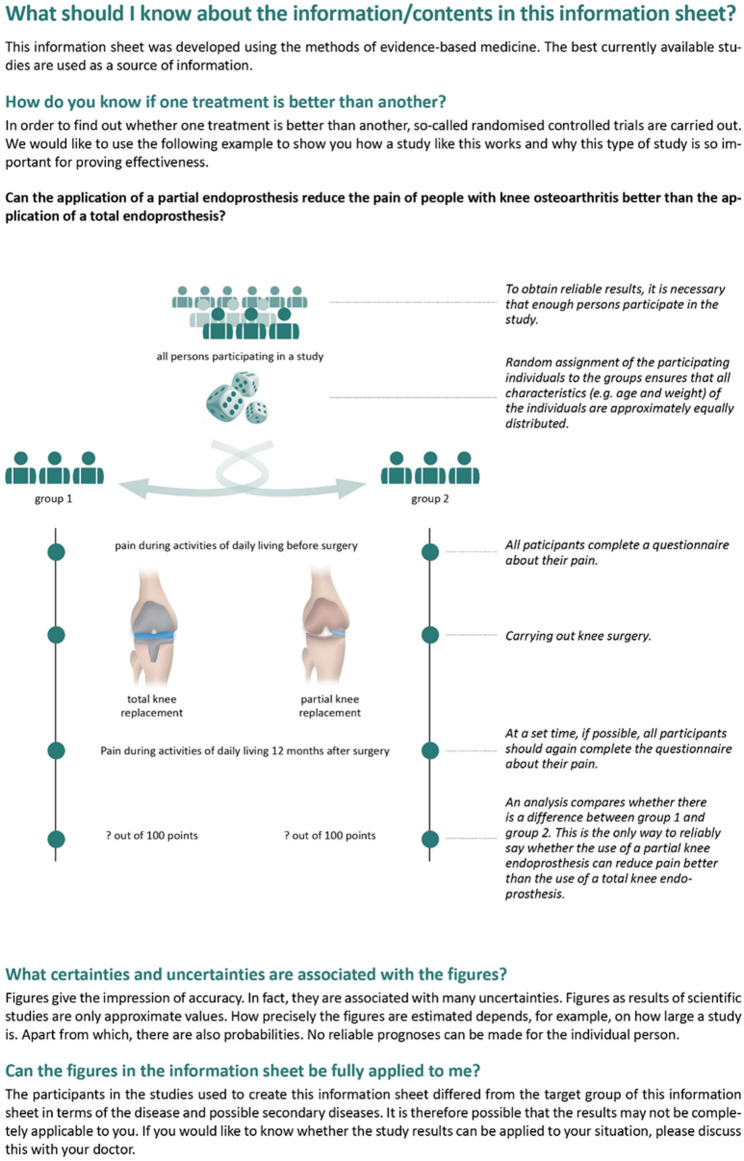
Revised section on the methodological background of the materials

In the informed consent forms, a section explains the methodological background of the materials and the uncertainties associated with the numerical information within the form. In the piloting it became clear that the users were not familiar with the concept of comparing different options, which could also have affected their understanding of the comparison of the benefits of different options. The description of an RCT and its necessity for the proof of efficacy in the form of a text was given less relevance in a first version of the materials and was only understood with difficulty. In the revision, the text was replaced by a simplified graphical representation of an RCT (Fig. 3). This led to an improved perception of the section, but the relevance and comprehension varied from interview to interview.

**Fig. 4:**
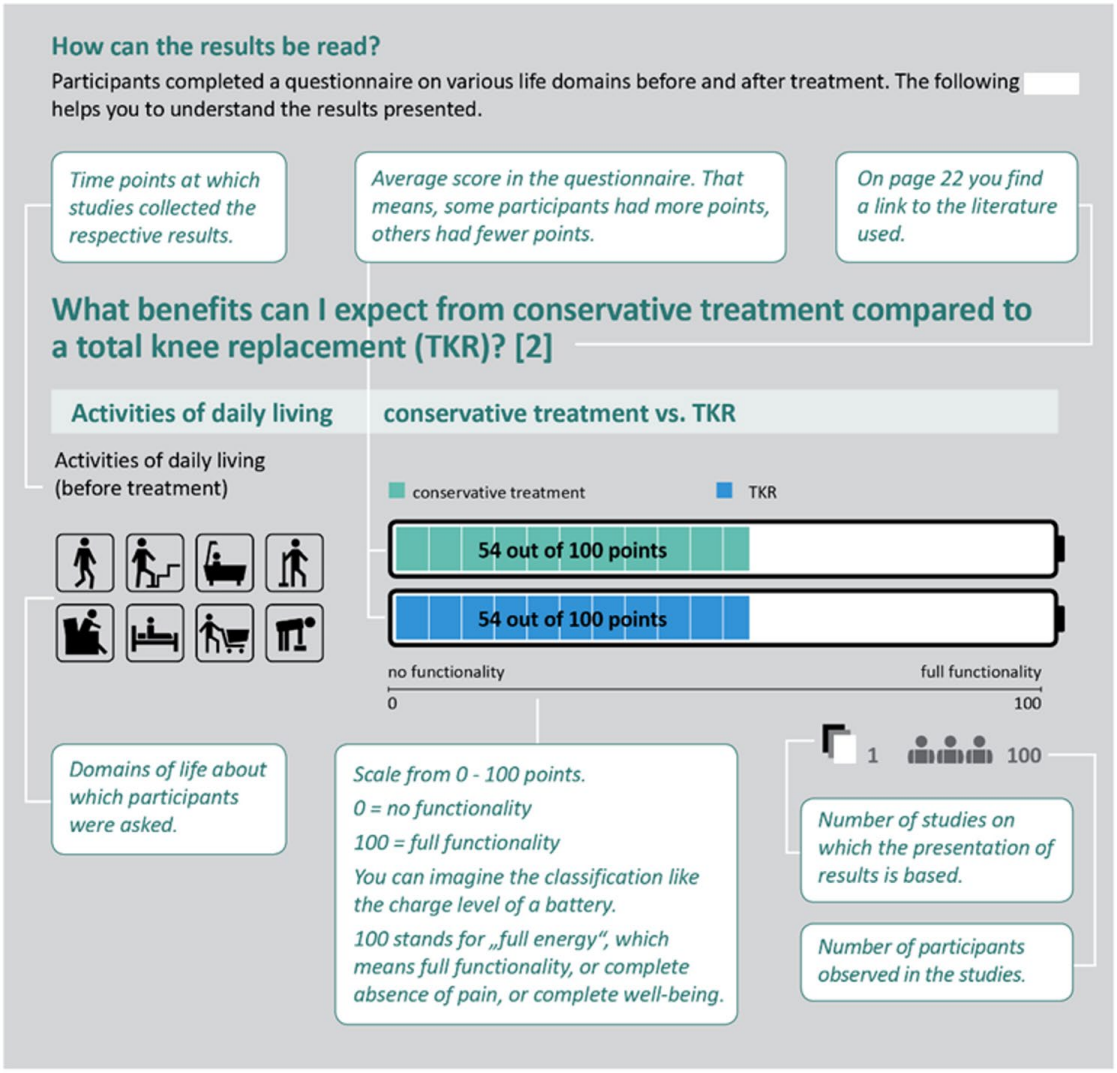
Revised explanation of the numerical representations and bar charts

In the first versions of the materials, the symbols used in the figures for the numerical representation of benefit and harm were listed and described on one page. Furthermore, the scales of the bar charts and their interpretation were explained. This type of explanation was not clear to all users or was sometimes not understood. In the revision, we therefore decided to label the use of the symbols in a specific application and to describe their meaning (Fig. 4). This led to a better perception of the legend and a better understanding.

**Fig. 5:**
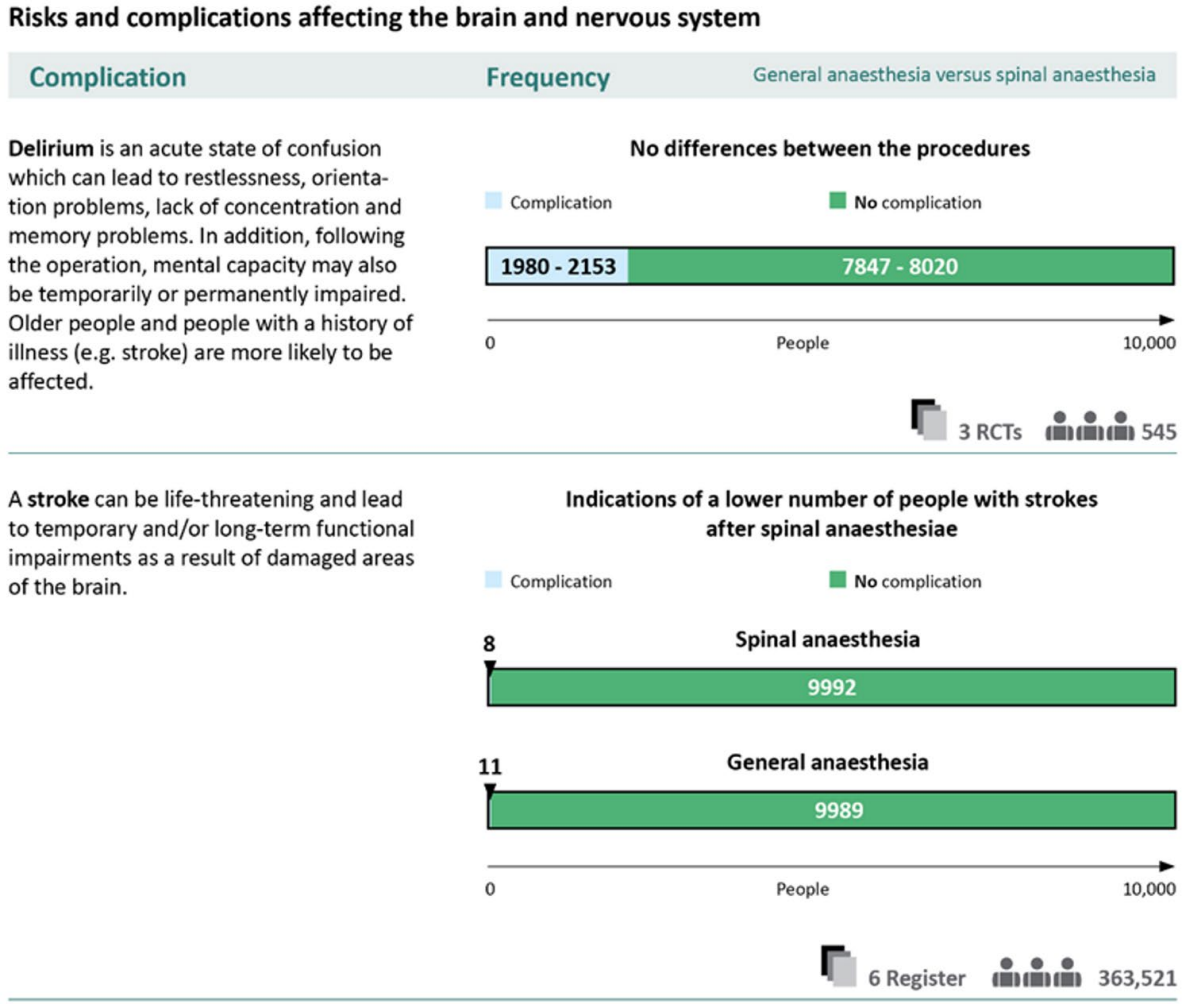
Revised labelling of graphical presentations

The presentation of numerical data on benefits and complications in the first versions of the materials sometimes caused major difficulties in terms of comprehensibility. In the presentation of complications, the reference to 1000 people, for example, was sometimes not apparent or was confused with the number of study participants. In the informed consent form for anaesthesia, the frequency of complications differed greatly in some cases, as a result of which the reference group was changed from 1000 to 10,000 in an initial version of the material. Despite clear labelling of the change in the reference group, some users did not notice this. In the revision, we therefore decided not to change the reference group within a material and to clarify the reference group in the labelling of the graphic. In addition, the information on the number of studies and participants in the studies to which the numerical data refers was coloured (greyed out) to avoid confusion (Fig. 5).

Users also reported problems understanding the ranges shown for the complications (Fig. 5). In order to improve comprehension, exemplary interpretation aids were created in text form, which describe in detail what information can be taken from the graphic.

**Fig. 6:**
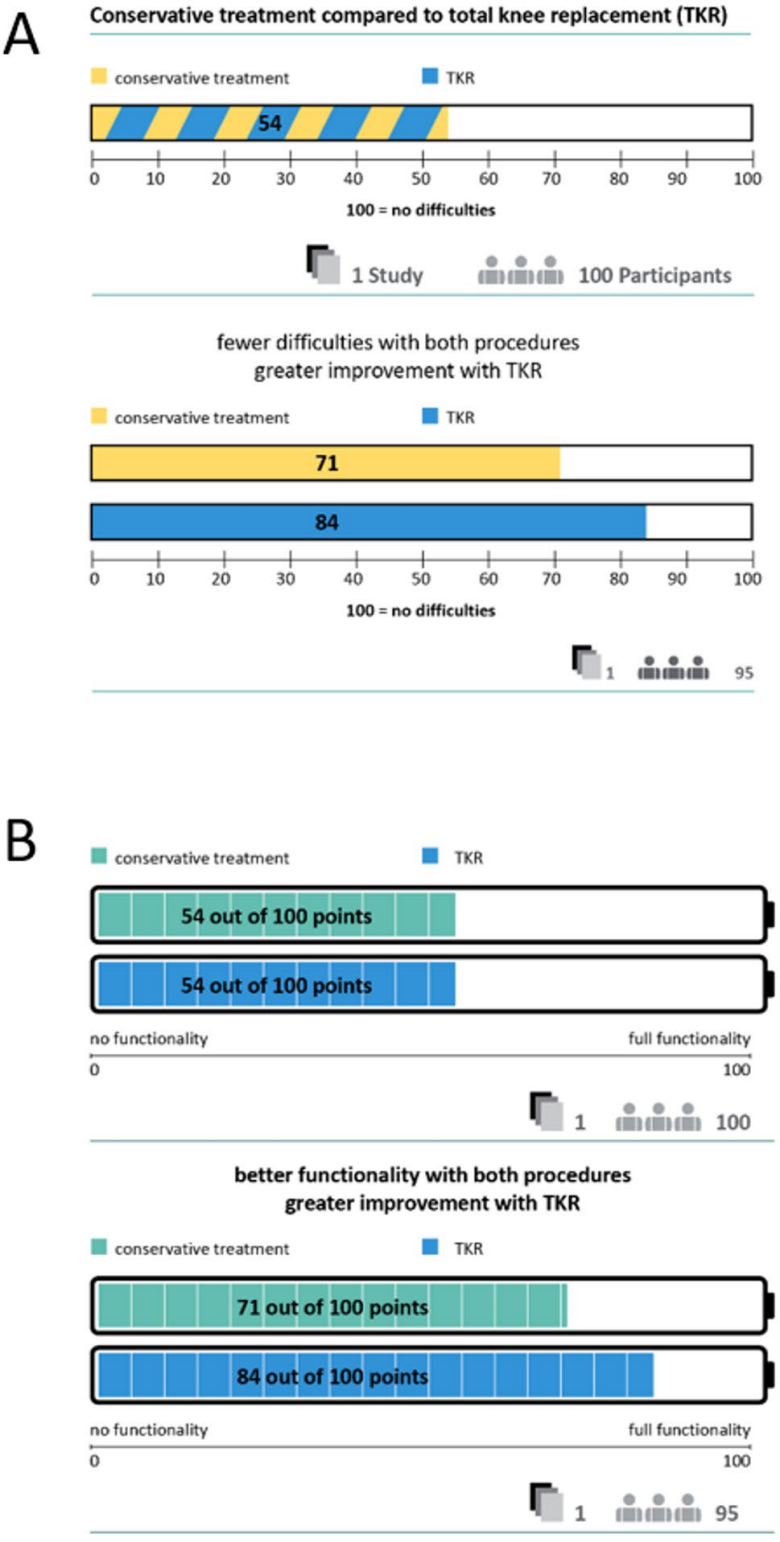
Progression of the revision of the bar charts from (A) first version to (B) final version

Another aspect that led to barriers to comprehensibility in the presentation of numerical data was the orientation of the scale for patient-relevant outcomes with regard to the benefit of treatment options. As the majority of the measurement instruments are ‘positive’, i.e. a higher score meant a better outcome, all outcomes were reported in this form. In some cases, it was not clear to users that numerical data referred to points in a questionnaire; instead, the number of people with difficulties was assumed. This was addressed in the revision by adding ‘points’ after the numerical data in the graphic and adding an exemplary interpretation of a bar chart. Furthermore, the orientation of the scales sometimes led to problems in comprehensibility. From experience with pain scales, where a high value means greater pain, this assumption was also transferred to the diagrams. In order to emphasise the ‘positive’ orientation of the scales, the labelling of the bar charts was adapted in the revision and the bar charts were designed in analogy to a battery charge level (Fig. 6). Subsequent interviews showed improved understanding as a result of these adjustments.

### Knowledge test

A questionnaire comprising 12 questions was developed, 8 questions on TKA and 4 questions on anaesthesia. The questions address the benefits and complications of surgery and anaesthesia in general, as well as explicitly requesting frequency information on benefits and complications (Additional file 5). Interviews were conducted with eight participants (6 female, 2 male) to assess comprehensibility. In an iterative revision process, some technical terms were exchanged or explained, answer options were revised, and the visualisation was adapted. As the test had to be developed before the informed consent forms were finalised so that it could be used in the pre-phase of the interrupted time series, some questions cannot be answered by the developed materials as planned.

### Training programme for the informed consent forms

An e-learning training programme was developed, covering information from a legal perspective, the structure and content of the informed consent forms, methods of evidence-based medicine, informed decision-making, risk communication and participation in medical decisions. An overview page provided participants with general information about the training programme, as well as introductions to the topic through two videos and access to the forum. The overview page also provided access to the learning module for the evidence-based informed consent forms. The training programme lasted between 90 and 180 min and was assigned 5 continuing medical education (CME) points. The duration could be adapted to the situation and the level of knowledge of the participants by skipping individual sections or pausing after individual chapters and continuing at a later time. The following learning objectives have been defined: At the end of the training the participants will be able to: (1) describe the structure and layout of the informed consent form, (2) state the basic principles of risk communication, (3) explain relative and absolute risk reduction (4) explain the five steps of evidence-based medicine, (5) explain the study designs RCT and systematic review, (6) explain the difference between patient-relevant outcomes and surrogate outcomes, (7) reflect on which outcomes can be considered as surrogate parameters in their field and how well their relationship with clinical outcomes has been studied, (8) describe the paternalistic and participatory models of decision making and (9) explain the concept of informed decision making.

## Discussion

The aim of this study was to develop and pilot test evidence-based informed consent forms for TKA and associated anaesthetic procedures. In addition, we prepared a knowledge test of patient understanding and risk perception and developed a training programme for healthcare professionals for clinical implementation in the interrupted time series pilot study. As a result of a systematic development process, we created an expanded informed consent form for TKA, consisting of an informed consent form and a supplementary information brochure, and a separate informed consent form for anaesthesia. These materials meet the criteria for evidence-based health information and, following extensive legal review, also satisfy the formal requirements for informed consent under German law. The content of the existing standard consent forms has been revised and substantially expanded based on patient´s information needs to provide patients with comprehensive information about surgery and anaesthesia, the alternatives available, and the potential benefits and harms of each option. In addition, the materials include evidence-based presentation techniques. In particular, benefits and complications are presented using natural frequencies and bar charts to aid comprehension. A key novelty compared with standard consent forms is the TKA information brochure, which introduces and provides a comparison of different surgical options and their expected outcomes. The materials were iteratively pilot tested with the target group and the results show that the materials were generally found to be understandable and helpful. At the same time, the results show that there are differences in information needs and expectations regarding the structure and scope of informed consent forms. This highlights the potential benefits of developing modular or adaptable materials that allow for tailored information provision. The introduction of web-based versions may be a viable solution for patients with sufficient digital literacy, allowing gradual access to content in line with individual preferences and readiness to make decisions.

Our approach to evidence-based informed consent forms differs from another “evidence-based” consent form described in the literature, where the term refers primarily to the legal aspects of consent [52]. In contrast, our materials go beyond providing legally sound consent by providing the information needed to make an informed decision. It can be argued that informed consent forms are only used to support verbal counselling and are therefore primarily for the legal protection of doctors, and that detailed information is provided at another, earlier stage. However, the literature and our exploration in the course of the project indicate that patients feel inadequately informed about their OAK, prognosis and treatment [26, 53, 54]. In addition, TKA is often perceived as an unavoidable treatment [26, 55–57] and conservative options are sometimes not offered or fully utilised [26, 54, 55, 58]. In addition, alternative sources of information, such as the internet, do not provide sufficient information for an informed decision [59]. Insufficient or inappropriate information can be a major obstacle to an informed decision [53]. A comprehensive informed consent form that provides information in a structured and easily understandable way, without bias, is essential in the information process and helps clinicians to meet legal requirements.

The results of our exploration of information processes show that informed consent forms are often presented just before the operation, when the patient’s decision has already been made [26]. In addition, a study from Turkey have shown considerable deficits in the use of informed consent forms for TKA in practice [60]. These findings point to the need for a paradigm shift in the use of informed consent forms in clinical care. Informed consent should not be seen as a single moment of signing, but rather as a dynamic process that begins as soon as the question of TKA arises. The materials we have developed, in particular the evidence-based informed consent forms and the accompanying information brochure, can be used to support this process and inform the early stages of outpatient decision making, provide structured support during the clinical consultation, and culminate in a well-informed signature. In this way, consent becomes a continuum, supporting patients throughout the decision-making process rather than simply documenting its completion.

A key strength of this project is the systematic, evidence-based development of the informed consent forms, which have been rigorously reviewed, iteratively optimised in consultation with clinical experts, and tested with target users. Bühn et al. conducted a systematic review of the design elements used in informed consent forms. They only included one study, published in 1998, which evaluated informed consent forms used in Germany. No studies have evaluated the current consent forms [61]. Nonetheless, several limitations must be acknowledged. Contrary to the original study protocol, the GRADE approach [32] was not applied to assess the quality of the evidence. The pilot testing took place during the COVID-19 pandemic and was conducted entirely online, which may have introduced selection bias in favour of digitally literate individuals. Recruitment via Facebook and eBay Classifieds, as well as the data collection methods, may also have resulted in a sample with specific characteristics, introducing further selection bias. The knowledge test was developed in parallel with the evidence synthesis and prior to the finalisation of the presentation formats, which limited the alignment of some items with the final materials. Furthermore, although the training programme for professionals was based on existing concepts, it was not itself formally piloted.

The evidence-based informed consent forms developed in this study were used as a template for the pilot study with an interrupted time series design [28], which was used to implement and evaluate evidence-based informed consent forms in routine clinical practice. After completion of the pilot study, the informed consent forms were no longer used at the hospital. As the systematic literature searches were conducted between 2020 and 2022, the searches should be updated, together with the consent forms if necessary. A complementary process evaluation explored facilitators and barriers to implementation from both patient and provider perspectives. The results are presented in separate publications, which also discuss the implications for future practice and implementation.

## Conclusion

This study demonstrates that the development of evidence-based and legally sound informed consent forms is complex and requires extensive resources, but is feasible, and that such materials are perceived by patients as helpful and understandable. At the same time, our results show that patients’ information needs and preferences for information delivery vary widely. This highlights the importance of individual, personal counselling to support informed decision-making, as well as investigating the possibilities and the feasibility of structuring informed consent forms modularly according to individual information needs. Structural and cultural changes in clinical practice are also emerging, which are necessary to realise the full potential of evidence-based consent forms. This includes the timing of informed consent and the scope of the information provided, including data on the frequency of benefits and complications. When used under the right conditions, the informed consent forms developed in this study can meaningfully support the process of informed decision-making.

## Supplementary Information

Below is the link to the electronic supplementary material.

Supplementary Material 1

Supplementary Material 2

Supplementary Material 3

Supplementary Material 4

Supplementary Material 5

## Data Availability

The datasets used and/or analysed during the current study are available from the corresponding author on reasonable request.

## Abbreviations

CReDECI: Criteria for reporting the development and evaluation of complex interventions in healthcare
CME: Continuing medical education
COREQ: Consolidated criteria for reporting qualitative research
NACOR: National Anaesthesia Clinical Outcomes Registry
OAK: Osteoarthritis of the knee
PROM: Patient-reported outcome measure
RCT: Randomised controlled trial
TKA: Total knee arthroplasty
