# Supplementary material for: Evidence-based informed consent forms for total knee arthroplasty and anaesthesia: development and pilot study
Source: J Orthop Surg Res. 2026 Feb 5;21:156. doi: 10.1186/s13018-026-06729-z (PMC12930710; doi:10.1186/s13018-026-06729-z)
Supplement: Supplementary file 1 — Supplementary Material 1 [file 13018_2026_6729_MOESM1_ESM.pdf]

Additional file 1: Details of the systematic evidence syntheses to obtain quantitative information for the consent forms

1. Extended informed consent form for TKA

Table 1 PICO scheme

**P**opulation	People with osteoarthritis of the knee
**I**ntervention	Conservative therapy (1); various types of prostheses (2-5); anchoring methods (6); surgical procedures (7-11) and treatment concepts (12)
**C**omparison	Standard types of prostheses; standard anchoring methods, standard surgical procedures and treatment concepts for the total knee arthroplasty (TKA)
**O**utcome	Patient-relevant outcomes (functionality, pain, quality of life, patient satisfaction, complications)

Table 2 PICO questions

No.	PICO question
1	Is total knee arthroplasty more effective than conservative treatment methods in people with osteoarthritis of the knee in terms of pain reduction, functional improvement, quality of life, patient satisfaction, and complications?
2	Is total knee arthroplasty more effective than unicondylar arthroplasty in people with osteoarthritis of the knee in terms of pain reduction, functional limitation, quality of life, patient satisfaction, and complications?
3	Is a non-constrained knee arthroplasty more effective than a semi-constrained (posterior stabilized) knee arthroplasty in people with osteoarthritis of the knee in terms of pain reduction, functional limitation, improvement in quality of life, patient satisfaction, and complications?
4	Is total knee arthroplasty with patellar resurfacing more effective than knee arthroplasty without patellar resurfacing in people with osteoarthritis of the knee in terms of pain reduction, reduction of functional impairment, improvement in quality of life, patient satisfaction, and complications?
5	Is total knee arthroplasty with mobile bearing more effective than knee arthroplasty with fixed bearing in people with osteoarthritis of the knee in terms of pain reduction, reduction of functional impairment, improvement in quality of life, patient satisfaction, and complications?
6	Is a cemented knee arthroplasty more effective than a cementless knee arthroplasty in people with osteoarthritis of the knee in terms of pain reduction, functional limitation, quality of life, patient satisfaction, and complications?
7	Is computer-assisted navigation for total knee arthroplasty in people with osteoarthritis of the knee more effective than the standard procedure for total knee arthroplasty in terms of pain reduction, reduction of functional impairment, improvement in quality of life, patient satisfaction, and complications?
8	Is the use of patient-specific instruments for total knee arthroplasty in people with osteoarthritis of the knee more effective than the standard procedure in terms of pain reduction, functional limitation, quality of life, patient satisfaction, and complications?
9	Is minimally invasive total knee arthroplasty more effective than conventional total knee arthroplasty in people with osteoarthritis of the knee in terms of pain reduction, functional improvement, quality of life, patient satisfaction, and complications?
10	Is robot-assisted navigation for total knee arthroplasty in people with osteoarthritis of the knee more effective than the standard procedure for total knee arthroplasty in terms of pain

	reduction, reduction of functional limitations, improvement in quality of life, patient satisfaction, and complications?
11	Is the use of patient-specific prostheses for total knee arthroplasty in people with osteoarthritis of the knee more effective than the standard procedure for total knee arthroplasty in terms of pain reduction, reduction of functional limitations, improvement in quality of life, patient satisfaction, and complications?
12	Is a fast track procedure for total knee arthroplasty in people with osteoarthritis of the knee more effective than the standard procedure in terms of pain reduction, reduction of functional limitations, improvement in quality of life, patient satisfaction, and complications?

Table 3 Inclusion and exclusion criteria

**Inclusion and exclusion criteria for title/abstract screening**	
Inclusion criteria:	
Population	-
Intervention	Conservative therapy
Comparison	Any form of total knee arthroplasty
Outcome	-
Study design	Systematic reviews, RCTs, prospective cohort studies, registry studies (for rare complications)
Language	German or English
**Overarching inclusion and exclusion criteria for full-text screening**	
Inclusion criteria:	
Population	People (≥ 18 years of age) with osteoarthritis of the knee
Intervention	Conservative therapy (1); various types of prostheses (2-5); anchoring methods (6); surgical procedures (7-11) and treatment concepts (12)
Comparison	Total knee arthroplasty
Outcome	Functionality, pain, quality of life, patient satisfaction, complications
Study design	Systematic reviews, RCTs, prospective cohort studies, registry studies
Language	German or English
Exclusion criteria:	
Population	People with rheumatoid arthritis or post-traumatic osteoarthritis of the knee (if these account for more than 20% of the study population)
Intervention	Revision surgery, conversion of a partial endoprosthesis to a total endoprosthesis, previous osteotomy on the affected leg, bilateral surgery (if performed in more than 20% of the study population)
Comparison	-

Clarification of inclusion and exclusion criteria based on the questions for full-text screening

Table 4 Inclusion and exclusion criteria for PICO question 1

Inclusion criteria:	
Intervention	Various kinds of physical exercises, various types of physiotherapy, weight reduction/diet therapy, orthopaedic technology, medication
Comparison	Any form of total knee arthroplasty
Exclusion criteria:	
Intervention	Holistic medicine, intra-articular injections
Comparison	-

Table 5 Inclusion and exclusion criteria for PICO question 2

Inclusion criteria:	
Intervention	Partial prosthesis (unicondylar prosthesis)
Comparison	Any form of total knee arthroplasty
Exclusion criteria:	
Intervention	Patellofemoral prosthesis, bicompartmental prosthesis
Comparison	-

Table 6 Inclusion and exclusion criteria for PICO question 3

Inclusion criteria:	
Intervention	Posterior stabilised total knee arthroplasty (any form)
Comparison	Any form of total knee arthroplasty retaining the posterior cruciate ligament
Exclusion criteria:	
Intervention	Special inlays (e.g. ultra-congruent inserts, medial pivot), changes to the anterior cruciate ligament
Comparison	Changes to the anterior cruciate ligament

Table 7 Inclusion and exclusion criteria for PICO question 4

Inclusion criteria:	
Intervention	Any form of total knee arthroplasty with substitution of the posterior of the patella
Comparison	Any form of total knee arthroplasty without substitution of the posterior of the patella
Exclusion criteria:	
Intervention	"Reshaping", electro-cauterization
Comparison	-

Table 8 Inclusion and exclusion criteria for PICO question 5

Inclusion criteria:	
Intervention	Any form of total knee arthroplasty with movable joint surface insert
Comparison	Any form of total knee arthroplasty with immovable joint surface insert
Exclusion criteria:	
Intervention	-
Comparison	-

Table 9 Inclusion and exclusion criteria for PICO question 6

Inclusion criteria:	
Intervention	Cemented prosthetic anchoring of both prosthesis components
Comparison	Cement-free prosthetic anchoring of both prosthesis components, "trabecular metal, hydroxyapatite coated, porous-coated"
Exclusion criteria:	
Intervention	Hybrid anchoring
Comparison	Hybrid anchoring

Table 10 Inclusion and exclusion criteria for PICO question 7

Inclusion criteria:	
Intervention	Any form of total knee arthroplasty with any form of computer-assisted navigation
Comparison	Any form of total knee arthroplasty with standard navigation
Exclusion criteria:	
Intervention	-
Comparison	-

Table 11 Inclusion and exclusion criteria for PICO question 8

Inclusion criteria:	
Intervention	Total knee arthroplasty with patient-specific cutting guides
Comparison	Any form of total knee arthroplasty with standard-cutting guides
Exclusion criteria:	
Intervention	-
Comparison	-

Table 12 Inclusion and exclusion criteria for PICO question 9

Inclusion criteria:	
Intervention	Any form of total knee arthroplasty with any type of minimally invasive access
Comparison	Any form of total knee arthroplasty with standard access
Exclusion criteria:	
Intervention	-
Comparison	-

Table 13 Inclusion and exclusion criteria for PICO question 10

Inclusion criteria:	
Intervention	Any form of total knee arthroplasty with any type of robot-assisted navigation
Comparison	Any form of total knee arthroplasty with standard navigation
Exclusion criteria:	
Intervention	
Comparison	-

Table 14 Inclusion and exclusion criteria for PICO question 11

Inclusion criteria:	
Intervention	Any form of total knee arthroplasty with prostheses made to fit the individual patient
Comparison	Any form of total knee arthroplasty with standard prostheses
Exclusion criteria:	
Intervention	Gender-specific types of prostheses without custom-made production
Comparison	-

Table 15 Inclusion and exclusion criteria for PICO question 12

Inclusion criteria:	
Intervention	Treatment pathways that combine pre-operative adjustments/offers (e.g., patient training), perioperative adjustments (e.g., spinal anaesthesia, minimally invasive access), and post-operative adjustments (e.g., intensive physical therapy, pain management)
Comparison	Standard treatment pathways
Exclusion criteria:	
Intervention	-
Comparison	-

Table 16 Search Strategy for MEDLINE, EMBASE and Epistemonikos

Database	Search strategy	Results
MEDLINE (via PubMed)	(("osteoarthritis, knee"[MeSH Terms] OR ("knee*"[Title/Abstract] AND ("osteoarthrosis"[Title/Abstract] OR "osteoarthritis"[Title/Abstract] OR "arthritis"[Title/Abstract] OR "arthrosis"[Title/Abstract])) OR "gonarthrosis"[Title/Abstract] OR "gonarthritis"[Title/Abstract]) AND (("knee*"[Title/Abstract] AND ("implant*"[Title/Abstract] OR "prosthe*"[Title/Abstract] OR "replacement*"[Title/Abstract] OR "arthroplasty"[Title/Abstract] OR "arthroplasties"[Title/Abstract])) OR "tka"[Title/Abstract] OR "tkr"[Title/Abstract] OR "arthroplasty, replacement, knee"[MeSH Terms]) AND ("MEDLINE"[Text Word] OR "systematic review"[Text Word] OR "meta-analysis"[Publication Type] OR "intervention*"[Title] OR (("randomized controlled trial"[Publication Type] OR "controlled clinical trial"[Publication Type] OR "randomized"[Title/Abstract] OR "randomised"[Title/Abstract] OR "placebo"[Title/Abstract] OR "clinical trials as topic"[MeSH Terms:noexp] OR "randomly"[Title/Abstract] OR "trial"[Title]) NOT ("animals"[MeSH Terms] NOT "humans"[MeSH Terms])) OR (("re-operation"[Title/Abstract] OR "Reoperation"[Title/Abstract] OR "re-admission"[Title/Abstract] OR "readmission"[Title/Abstract] OR "death"[Title/Abstract] OR "mortality"[Title/Abstract] OR "bleeding"[Title/Abstract] OR "loss of blood"[Title/Abstract] OR "blood loss"[Title/Abstract] OR "wound complication*"[Title/Abstract] OR "thromboembolic disease"[Title/Abstract] OR "thromboembolic event*"[Title/Abstract] OR "thrombosis"[Title/Abstract] OR "embolism"[Title/Abstract] OR "pneumonia"[Title/Abstract] OR "cardiac event"[Title/Abstract] OR "myocardial infarction"[Title/Abstract] OR "neural deficit"[Title/Abstract] OR "instability"[Title/Abstract] OR "malalignment"[Title/Abstract] OR "stiffness"[Title/Abstract] OR "disruption"[Title/Abstract] OR "dislocation"[Title/Abstract] OR "bearing surface wear"[Title/Abstract] OR "osteolysis"[Title/Abstract] OR "implant loosening"[Title/Abstract] OR "allergy"[Title/Abstract] OR "allergies"[Title/Abstract] OR "intolerance"[Title/Abstract] OR "infection*"[Title/Abstract] OR "sepsis"[Title/Abstract] OR "fracture*"[Title/Abstract] OR "injury"[Title/Abstract] OR "injuries"[Title/Abstract] OR "compartment syndrome"[Title/Abstract] OR "complication*"[Title/Abstract] OR "adverse event*"[Title/Abstract] OR "adverse reaction*"[Title/Abstract] OR "adverse effect*"[Title/Abstract] OR "harm*"[Title/Abstract] OR	7044

	("Intraoperative Complications"[MeSH Terms:noexp] OR "blood loss, surgical"[MeSH Terms:noexp] OR "Postoperative Hemorrhage"[MeSH Terms:noexp] OR "Prosthesis Failure"[MeSH Terms:noexp] OR "Prosthesis-Related Infections"[MeSH Terms:noexp] OR "Surgical Wound Infection"[MeSH Terms:noexp] OR "Reoperation"[MeSH Terms:noexp]) OR ("arthroplasty, replacement, knee/adverse effects"[MeSH Terms] OR "arthroplasty, replacement, knee/mortality"[MeSH Terms] OR "knee prosthesis/adverse effects"[MeSH Terms:noexp] OR "knee prosthesis/mortality"[MeSH Terms] OR "thromboembolism/complications"[MeSH Terms:noexp] OR "thromboembolism/mortality"[MeSH Terms:noexp] OR "myocardial infarction/complications"[MeSH Terms:noexp] OR "myocardial infarction/mortality"[MeSH Terms:noexp] OR "sepsis/complications"[MeSH Terms:noexp] OR "sepsis/mortality"[MeSH Terms:noexp] OR "compartment syndromes/complications"[MeSH Terms:noexp] OR "compartment syndromes/mortality"[MeSH Terms:noexp]) OR ("complication*"[Title/Abstract] OR "adverse effects"[MeSH Subheading] OR "safe*"[Title/Abstract] OR "complications"[MeSH Subheading] OR "Postoperative Complications"[MeSH Terms:noexp])) AND (("cohort studies"[MeSH Terms] OR ("cohort"[All Fields] AND "studies"[All Fields]) OR "cohort studies"[All Fields] OR "cohort"[All Fields] OR "cohort s"[All Fields] OR "cohorte"[All Fields] OR "cohorts"[All Fields] OR (("controling"[All Fields] OR "controllability"[All Fields] OR "controllable"[All Fields] OR "controllably"[All Fields] OR "controller"[All Fields] OR "controller s"[All Fields] OR "controllers"[All Fields] OR "controlling"[All Fields] OR "controls"[All Fields] OR "prevention and control"[MeSH Subheading] OR ("prevention"[All Fields] AND "control"[All Fields]) OR "prevention and control"[All Fields] OR "control"[All Fields] OR "control groups"[MeSH Terms] OR ("control"[All Fields] AND "groups"[All Fields]) OR "control groups"[All Fields]) AND ("studies"[All Fields] OR "study"[All Fields] OR "study s"[All Fields] OR "studying"[All Fields] OR "studys"[All Fields])) OR ("control"[Text Word] AND "group*"[Text Word]) OR "epidemiologic studies"[MeSH Terms] OR "program"[Text Word] OR "clinical trial"[Publication Type] OR "comparative stud*"[All Fields] OR ("evaluation study"[Publication Type] OR "evaluation studies as topic"[MeSH Terms] OR "evaluation studies"[All Fields]) OR "statistics as topic"[MeSH Terms] OR "survey*"[Text Word] OR "follow up*"[All Fields] OR ("time factors"[MeSH Terms] OR ("time"[All Fields] AND "factors"[All Fields]) OR "time factors"[All Fields]) OR "ci"[Text Word]) NOT (("animals"[MeSH Terms:noexp] NOT "humans"[MeSH Terms:noexp]) OR "comment"[Publication Type] OR "editorial"[Publication Type] OR "review"[Publication Type] OR "meta-analysis"[Publication Type] OR "case report"[Text Word] OR "consensus"[MeSH Terms] OR		

	"guideline"[Publication Type] OR "history"[MeSH Subheading]))))) NOT ("comment"[Publication Type] OR "letter"[Publication Type] OR "editorial"[Publication Type])	
EMBASE (via Elsevier)	('knee osteoarthritis'/de OR ((knee* NEAR/4 (osteoarthrosis OR osteoarthritis OR arthrosis OR arthritis)):ti,ab,kw) OR gonarthrosis:ti,ab,kw OR gonarthritis:ti,ab,kw) AND ('knee replacement'/de OR 'total knee arthroplasty'/de OR ((knee* NEAR/4 (replacement? OR arthroplasty OR arthroplasties OR implant* OR prosthes*)):ti,ab,kw) OR tka:ti,ab,kw OR tkr:ti,ab,kw) AND (random*:ti,ab,kw OR placebo*:de,ti,ab,kw OR ((double NEXT/1 blind*):ti,ab,kw) OR ((re?operation:ti,ab,kw OR re?admission:ti,ab,kw OR death:ti,ab,kw OR mortality:ti,ab,kw OR bleeding:ti,ab,kw OR 'loss of blood':ti,ab,kw OR 'blood loss':ti,ab,kw OR 'wound complication?':ti,ab,kw OR 'thromboembolic disease':ti,ab,kw OR 'thromboembolic event?':ti,ab,kw OR thrombosis:ti,ab,kw OR embolism:ti,ab,kw OR pneumonia:ti,ab,kw OR 'cardiac event?':ti,ab,kw OR 'myocardial infarction':ti,ab,kw OR 'neural deficit?':ti,ab,kw OR instability:ti,ab,kw OR malalignment:ti,ab,kw OR stiffness:ti,ab,kw OR disruption:ti,ab,kw OR dislocation:ti,ab,kw OR 'bearing surface wear':ti,ab,kw OR osteolysis:ti,ab,kw OR 'implant loosening':ti,ab,kw OR allergy:ti,ab,kw OR allergies:ti,ab,kw OR intolerance:ti,ab,kw OR infection?:ti,ab,kw OR sepsis:ti,ab,kw OR fracture*:ti,ab,kw OR injury:ti,ab,kw OR injuries:ti,ab,kw OR 'compartment syndrom':ti,ab,kw OR complication?:ti,ab,kw OR 'adverse event?':ti,ab,kw OR 'adverse reaction?':ti,ab,kw OR 'adverse effect?':ti,ab,kw OR harm?:ti,ab,kw OR 'postoperative nausea and vomiting'/de OR 'prosthesis complication'/exp OR 'knee osteoarthritis'/exp/dm_co,dm_si,dm_su OR 'blood vessel injury'/dm_co OR 'joint dislocation'/dm_co OR 'joint instability'/exp/dm_co OR 'joint stiffness'/dm_co OR 'ligament injury'/exp/dm_co OR 'postoperative hemorrhage'/dm_co OR 'postoperative infection'/dm_co OR 'prosthesis infection'/exp/dm_co OR 'surgical infection'/dm_co OR 'surgical injury'/dm_co OR 'thromboembolism'/dm_co OR 'wound complication'/dm_co OR 'reoperation'/de OR 'hospital readmission'/de OR 'operative blood loss'/dm_co OR 'heart infarction'/dm_co OR 'allergy'/dm_co OR 'sepsis'/dm_co OR 'compartment syndrome'/dm_co OR 'mortality'/de OR complication*:ti,ab,kw OR 'complication'/exp/dm_co OR safe*:ti,ab,kw OR 'adverse drug reaction'/exp/dm_co,dm_si OR 'post-operative morbidity':ti,ab,kw OR 'surgical risk'/de OR 'complication'/de OR 'postoperative complication'/de OR 'procedure related':ti,ab,kw) AND (cohort OR (cohort AND study) OR (control:ti,ab,kw,de,it AND group*:ti,ab,kw,de,it) OR 'epidemiology'/de OR 'retrospective study'/de OR 'prospective study'/de OR 'longitudinal study'/de OR 'cohort analysis'/de OR 'case control study'/de OR program:ti,ab,kw,de,it OR 'clinical trial':it OR 'comparative	497

	stud*' OR 'evaluation studies' OR 'statistics'/de OR survey*:ti,ab,kw,de,it OR 'follow-up' OR 'time factors' OR ci:ti,ab,kw,de,it) NOT ('animal'/de NOT 'human'/de OR comment:it OR editorial:it OR review:it OR 'meta analysis':it OR 'case report':ti,ab,kw,de,it OR 'consensus'/de OR guideline:it OR 'history'/de))) AND embase NOT (embase AND medline) AND ('article'/it OR 'article in press'/it)	
Epistemonikos	(advanced_title_en:(((knee* AND (osteoarthrosis OR osteoarthritis OR arthrosis OR arthritis)) OR gonarthrosis OR gonarthritis)) OR advanced_abstract_en:(((knee* AND (osteoarthrosis OR osteoarthritis OR arthrosis OR arthritis)) OR gonarthrosis OR gonarthritis))) AND (advanced_title_en:(((knee* AND (implant* OR prosthe* OR replacement* OR arthroplasty OR arthroplasties)) OR TKA OR TKR)) OR advanced_abstract_en:(((knee* AND (implant* OR prosthe* OR replacement* OR arthroplasty OR arthroplasties)) OR TKA OR TKR))) [Filters: classification=systematic-review, protocol=no]	444

Search date: 5 November 2020

Table 17 Search Strategy for CENTRAL

Database	#	Search strategy	Results
CENTRAL (via Cochrane Library)	#1	((osteoarthritis NEAR/4 knee*)):ti,ab,kw	10625
	#2	(osteoarthrosis NEAR/4 knee*):ti,ab,kw	125
	#3	(arthritis NEAR/4 knee*):ti,ab,kw	634
	#4	(arthrosis NEAR/4 knee*):ti,ab,kw	257
	#5	gonarthrosis:ti,ab,kw	471
	#6	gonarthritis:ti,ab,kw	48
	#7	#1 OR #2 OR #3 OR #4 OR #5 OR #6	11206
	#8	(replacement? NEAR/4 knee*):ti,ab,kw	4981
	#9	((arthroplasty OR arthroplasties) NEAR/4 knee*):ti,ab,kw	6781
	#10	(implant* NEAR/4 knee*):ti,ab,kw	351
	#11	(prosthesis NEAR/4 knee*):ti,ab,kw	1222
	#12	tka:ti,ab,kw	2766
	#13	tkr:ti,ab,kw	612
	#14	#8 OR #9 OR #10 OR #11 OR #12 OR #13	8480
	#15	#7 AND #14 in Trials	2493
	#16	Source: CINAHL	11

Search date: 5 November 2020

Table 18 Search strategy for the update search for systematic reviews

**Database**	**Search strategy**	**Results**
MEDLINE (via PubMed)	((("osteoarthritis, knee"[MeSH Terms] OR ("knee*"[Title/Abstract] AND ("osteoarthrosis"[Title/Abstract] OR "osteoarthritis"[Title/Abstract] OR "arthritis"[Title/Abstract] OR "arthrosis"[Title/Abstract])) OR "gonarthrosis"[Title/Abstract] OR "gonarthritis"[Title/Abstract]) AND (("knee*"[Title/Abstract] AND ("implant*"[Title/Abstract] OR "prosthe*"[Title/Abstract] OR "replacement*"[Title/Abstract] OR "arthroplasty"[Title/Abstract] OR "arthroplasties"[Title/Abstract])) OR "tka"[Title/Abstract] OR "tkr"[Title/Abstract] OR "arthroplasty, replacement, knee"[MeSH Terms]) AND ("MEDLINE"[Text Word] OR "systematic review"[Text Word] OR "meta-analysis"[Publication Type] OR "intervention*"[Title])) NOT ("comment"[Publication Type] OR "letter"[Publication Type] OR "editorial"[Publication Type])) AND 2020/11/05:3000/12/31[Date - Publication]	48
Epistemonikos	(title:(((knee* AND (osteoarthrosis OR osteoarthritis OR arthrosis OR arthritis)) OR gonarthrosis OR gonarthritis)) OR abstract:(((knee* AND (osteoarthrosis OR osteoarthritis OR arthrosis OR arthritis)) OR gonarthrosis OR gonarthritis))) AND<br>(title:(((knee* AND (implant* OR prosthe* OR replacement* OR arthroplasty OR arthroplasties)) OR TKA OR TKR)) OR abstract:(((knee* AND (implant* OR prosthe* OR replacement* OR arthroplasty OR arthroplasties)) OR TKA OR TKR)))<br>Filter: Systematic Reviews<br>Filter: Custom date: 2020/11/05-2021/03/30	34

Search date: 30 March 2021

2. Informed consent form for anaesthesia

2.1 Spinal anaesthesia compared to general anaesthesia:

Table 19 PICO scheme

**P**opulation	People undergoing elective knee or hip replacement surgery
**I**ntervention	Spinal anaesthesia (epidural anaesthesia for perioperative complications)
**C**omparison	General anaesthesia
**O**utcome	Perioperative and postoperative complications up to 30 days post-OP

Table 20 Inclusion and exclusion criteria

	**Inclusion criteria**
Population	Age ≥ 18 years, elective knee or hip replacement surgery (at least 80% of the study population)
Intervention	Spinal anaesthesia, combined spinal/epidural anaesthesia or epidural anaesthesia for perioperative outcomes, spinal anaesthesia for postoperative outcomes
Comparison	General anaesthesia
Outcome	All perioperative or postoperative complications, e.g.:<br>PONV, urinary retention, itching, delirium, mortality, cardiac complications, pulmonary complications, stroke, renal failure
Study design	Systematic reviews, RCTs, prospective cohort studies
Language	German or English
	**Exclusion criterial**
Intervention	Epidural anaesthesia for postoperative outcomes, combined spinal-/epidural anaesthesia for postoperative outcomes, other forms of local anaesthesia (e.g. lumbar plexus block, adductor canal block), data collection began before 1990

Table 21 Search strategy for systematic reviews for each database

Database	Search strategy	Results
MEDLINE (via PubMed)	((("knee*"[Title/Abstract] OR "hip"[Title/Abstract] OR "hips"[Title/Abstract]) AND ("implant*"[Title/Abstract] OR "prosthe*"[Title/Abstract] OR "replacement*"[Title/Abstract] OR "arthroplast*"[Title/Abstract])) OR ("tka"[Title/Abstract] OR "tkr"[Title/Abstract] OR "uka"[Title/Abstract] OR "ukr"[Title/Abstract] OR "thr"[Title/Abstract] OR "tha"[Title/Abstract] OR "arthroplasty, replacement, knee"[MeSH Terms] OR "arthroplasty, replacement, hip"[MeSH Terms])) AND ((("anesthesia"[Title/Abstract] OR "anaesthesia"[Title/Abstract]) AND ("general"[Title/Abstract] OR "spinal"[Title/Abstract] OR "intrathecal"[Title/Abstract] OR "regional"[Title/Abstract] OR "neuraxial"[Title/Abstract] OR "conduction"[Title/Abstract])) OR ("spinal block*"[Title/Abstract] OR "subarachnoid block*"[Title/Abstract] OR "intradural block*"[Title/Abstract] OR "intrathecal block*"[Title/Abstract]) OR ("anesthesia, general"[MeSH Terms] OR "anesthesia, conduction"[MeSH Terms] OR "anesthesia, spinal"[MeSH Terms])) AND ("meta analysis"[Publication Type] OR "meta analysis"[Title/Abstract] OR "meta analysis as topic"[MeSH Terms] OR "review"[Publication Type] OR "search*"[Title/Abstract])	315
Epistemonikos	(title:((((knee OR hips OR hip) AND (implant* OR prosthe* OR replacement* OR arthroplast*)) OR tka OR uka OR tkr OR ukr OR thr OR tha)) OR abstract:((((knee OR hips OR hip) AND (implant* OR prosthe* OR replacement* OR arthroplast*)) OR tka OR uka OR tkr OR ukr OR thr OR tha))) AND (title:((((anesthesia OR anaesthesia) AND (general OR spinal OR intratecal OR regional OR conduction OR neuraxial)) OR ((spinal OR subarachnoid OR intradural OR intrathecal) AND block*))) OR abstract:((((anesthesia OR anaesthesia) AND (general OR spinal OR intratecal OR regional OR conduction OR neuraxial)) OR ((spinal OR subarachnoid OR intradural OR intrathecal) AND block*))))<br><br>Filter: systematic review	52

Search date: 22 October 2021

Table 22 Search strategy for randomised controlled trials and controlled non-randomised trials for MEDLINE

Database	Search strategy	Results
MEDLINE (via PubMed)	((((("knee*"[Title/Abstract] OR "hip"[Title/Abstract] OR "hips"[Title/Abstract]) AND ("implant*"[Title/Abstract] OR "prosthe*"[Title/Abstract] OR "replacement*"[Title/Abstract] OR "arthroplast*"[Title/Abstract])) OR ("tka"[Title/Abstract] OR "tkr"[Title/Abstract] OR "uka"[Title/Abstract] OR "ukr"[Title/Abstract] OR "thr"[Title/Abstract] OR "tha"[Title/Abstract] OR "arthroplasty, replacement, knee"[MeSH Terms] OR "arthroplasty, replacement, hip"[MeSH Terms])) AND ((("anesthesia"[Title/Abstract] OR "anaesthesia"[Title/Abstract]) AND ("general"[Title/Abstract] OR "spinal"[Title/Abstract] OR "intrathecal"[Title/Abstract] OR "regional"[Title/Abstract] OR "neuraxial"[Title/Abstract] OR "conduction"[Title/Abstract])) OR ("spinal block*"[Title/Abstract] OR "subarachnoid block*"[Title/Abstract] OR "intradural block*"[Title/Abstract] OR "intrathecal block*"[Title/Abstract]) OR ("anesthesia, general"[MeSH Terms] OR "anesthesia, conduction"[MeSH Terms] OR "anesthesia, spinal"[MeSH Terms])) AND (("randomized controlled trial"[Publication Type] OR "controlled clinical trial"[Publication Type] OR "randomized"[Title/Abstract] OR "randomised"[Title/Abstract] OR "placebo"[Title/Abstract] OR "clinical trials as topic"[MeSH Terms:noexp] OR "randomly"[Title/Abstract] OR "trial"[Title]) NOT ("animals"[MeSH Terms] NOT "humans"[MeSH Terms])) AND 2018/05/01:3000/12/31[Date - Publication]) OR (((("knee*"[Title/Abstract] OR "hip"[Title/Abstract] OR "hips"[Title/Abstract]) AND ("implant*"[Title/Abstract] OR "prosthe*"[Title/Abstract] OR "replacement*"[Title/Abstract] OR "arthroplast*"[Title/Abstract])) OR ("tka"[Title/Abstract] OR "tkr"[Title/Abstract] OR "uka"[Title/Abstract] OR "ukr"[Title/Abstract] OR "thr"[Title/Abstract] OR "tha"[Title/Abstract] OR "arthroplasty, replacement, knee"[MeSH Terms] OR "arthroplasty, replacement, hip"[MeSH Terms])) AND ((("anesthesia"[Title/Abstract] OR "anaesthesia"[Title/Abstract]) AND ("general"[Title/Abstract] OR "spinal"[Title/Abstract] OR "intrathecal"[Title/Abstract] OR "regional"[Title/Abstract] OR "neuraxial"[Title/Abstract] OR "conduction"[Title/Abstract])) OR ("spinal block*"[Title/Abstract] OR "subarachnoid block*"[Title/Abstract] OR "intradural block*"[Title/Abstract] OR "intrathecal block*"[Title/Abstract]) OR ("anesthesia, general"[MeSH Terms] OR "anesthesia, conduction"[MeSH Terms] OR "anesthesia, spinal"[MeSH Terms])) AND (("cohort studies"[MeSH Terms] OR ("cohort"[All Fields] AND "studies"[All Fields]) OR "cohort studies"[All Fields] OR	1485

	"cohort"[All Fields] OR "cohort s"[All Fields] OR "cohorte"[All Fields] OR "cohorts"[All Fields] OR (("controling"[All Fields] OR "controllability"[All Fields] OR "controllable"[All Fields] OR "controllably"[All Fields] OR "controller"[All Fields] OR "controller s"[All Fields] OR "controllers"[All Fields] OR "controlling"[All Fields] OR "controls"[All Fields] OR "prevention and control"[MeSH Subheading] OR ("prevention"[All Fields] AND "control"[All Fields]) OR "prevention and control"[All Fields] OR "control"[All Fields] OR "control groups"[MeSH Terms] OR ("control"[All Fields] AND "groups"[All Fields]) OR "control groups"[All Fields]) AND ("studies"[All Fields] OR "study"[All Fields] OR "study s"[All Fields] OR "studying"[All Fields] OR "studys"[All Fields])) OR ("control"[Text Word] AND "group*"[Text Word]) OR "epidemiologic studies"[MeSH Terms] OR "program"[Text Word] OR "clinical trial"[Publication Type] OR "comparative stud*"[All Fields] OR ("evaluation study"[Publication Type] OR "evaluation studies as topic"[MeSH Terms] OR "evaluation studies"[All Fields]) OR "statistics as topic"[MeSH Terms] OR "survey*"[Text Word] OR "follow up*"[All Fields] OR ("time factors"[MeSH Terms] OR ("time"[All Fields] AND "factors"[All Fields]) OR "time factors"[All Fields]) OR "ci"[Text Word]) NOT (("animals"[MeSH Terms:noexp] NOT "humans"[MeSH Terms:noexp]) OR "comment"[Publication Type] OR "editorial"[Publication Type] OR "review"[Publication Type] OR "meta analysis"[Publication Type] OR "case report"[Text Word] OR "consensus"[MeSH Terms] OR "guideline"[Publication Type] OR "history"[MeSH Subheading])) AND ("complication*"[Title/Abstract] OR "adverse effects"[MeSH Subheading] OR "safe*"[Title/Abstract] OR "complications"[MeSH Subheading] OR "Postoperative Complications"[MeSH Terms:noexp] OR ("injur*"[Title/Abstract] OR "airway obstruction*"[Title/Abstract] OR "airway trauma"[Title/Abstract] OR "anaphyla*"[Title/Abstract] OR "arrhythmia*"[Title/Abstract] OR "aspiration*"[Title/Abstract] OR "aware*"[Title/Abstract] OR "bradycard*"[Title/Abstract] OR "cardiac arrest*"[Title/Abstract] OR "infection*"[Title/Abstract] OR "cerebrovasular accident*"[Title/Abstract] OR "stroke*"[Title/Abstract] OR "coma*"[Title/Abstract] OR "death"[Title/Abstract] OR "died"[Title/Abstract] OR "mortality"[Title/Abstract] OR "delir*"[Title/Abstract] OR "itching"[Title/Abstract] OR "puritus*"[Title/Abstract] OR "kidney failure*"[Title/Abstract] OR "renal failure*"[Title/Abstract] OR "organ failure*"[Title/Abstract] OR "myocardial infarction*"[Title/Abstract] OR "myocardial ischemia*"[Title/Abstract] OR "visual loss"[Title/Abstract] OR "neurologic deficit*"[Title/Abstract] OR "pneumonia"[Title/Abstract] OR "nausea"[Title/Abstract] OR "vomitting"[Title/Abstract] OR "ponv"[Title/Abstract] OR "pdnv"[Title/Abstract] OR "headache"[Title/Abstract] OR	

	"cognitive disfunction*"[Title/Abstract] OR "edema"[Title/Abstract] OR "emboli*"[Title/Abstract] OR "embolus"[Title/Abstract] OR "respiratory failure*"[Title/Abstract] OR "seizure*"[Title/Abstract] OR "sepsis"[Title/Abstract] OR "shivering"[Title/Abstract] OR "spinal hematom*"[Title/Abstract] OR "cardiac arrest*"[Title/Abstract] OR "meningitis"[Title/Abstract] OR "paralys*"[Title/Abstract] OR "allergy"[Title/Abstract] OR "allergic"[Title/Abstract]))))) NOT (((("knee*"[Title/Abstract] OR "hip"[Title/Abstract] OR "hips"[Title/Abstract]) AND ("implant*"[Title/Abstract] OR "prosthe*"[Title/Abstract] OR "replacement*"[Title/Abstract] OR "arthroplast*"[Title/Abstract])) OR ("tka"[Title/Abstract] OR "tkr"[Title/Abstract] OR "uka"[Title/Abstract] OR "ukr"[Title/Abstract] OR "thr"[Title/Abstract] OR "tha"[Title/Abstract] OR "arthroplasty, replacement, knee"[MeSH Terms] OR "arthroplasty, replacement, hip"[MeSH Terms])) AND ((("anesthesia"[Title/Abstract] OR "anaesthesia"[Title/Abstract]) AND ("general"[Title/Abstract] OR "spinal"[Title/Abstract] OR "intrathecal"[Title/Abstract] OR "regional"[Title/Abstract] OR "neuraxial"[Title/Abstract] OR "conduction"[Title/Abstract])) OR ("spinal block*"[Title/Abstract] OR "subarachnoid block*"[Title/Abstract] OR "intradural block*"[Title/Abstract] OR "intrathecal block*"[Title/Abstract]) OR ("anesthesia, general"[MeSH Terms] OR "anesthesia, conduction"[MeSH Terms] OR "anesthesia, spinal"[MeSH Terms])) AND ("meta analysis"[Publication Type] OR "meta analysis"[Title/Abstract] OR "meta analysis as topic"[MeSH Terms] OR "review"[Publication Type] OR "search*"[Title/Abstract]))	

Search date: 8 February 2022

Table 23 Search strategy for randomised controlled trials and controlled non-randomised trials for EMBASE and CENTRAL

Database	#	Search strategy	Results
EMBASE (via Elsevier)	#1	'knee replacement'/exp OR 'knee replacement' OR 'total knee arthroplasty'/exp OR 'total knee arthroplasty' OR 'hip replacement'/exp OR 'hip replacement' OR (((knee* OR hips OR hip) NEAR/4 (replacement? OR arthroplasty OR arthroplasties OR implant* OR prosthes*)):ti,ab,kw) OR tka:ti,ab,kw OR tkr:ti,ab,kw OR uka:ti,ab,kw OR ukr:ti,ab,kw OR thr:ti,ab,kw OR tha:ti,ab,kw	137103
	#2	'general anesthesia'/exp OR 'spinal anesthesia'/exp OR 'epidural anesthesia'/exp	150774
	#3	((general OR spinal OR intrathecal OR regional OR neuraxial OR conduction) NEXT/2 (anesthesia OR anaesthesia)):ti,ab,kw	111562
	#4	'spinal block*':ti,ab,kw OR 'subarachnoid block*':ti,ab,kw OR 'intradural block*':ti,ab,kw OR 'intrathecal block*':ti,ab,kw	2501
	#5	#2 OR #3 OR #4	191417
	#6	random*:ti,ab,kw OR placebo*:de,ti,ab,kw OR ((double NEXT/1 blind*):ti,ab,kw)	2030806
	#7	#1 AND #5 AND #6	1320
	#8	[1-5-2018]/sd	7685597
	#9	#7 AND #8	340
	#10	[embase]/lim	28382280
	#11	#9 AND #10	305
	#12	(cohort OR (cohort AND study) OR (control:ti,ab,kw,de,it AND group*:ti,ab,kw,de,it) OR 'epidemiology'/de OR 'retrospective study'/de OR 'prospective study'/de OR 'longitudinal study'/de OR 'cohort analysis'/de OR 'case control study'/de OR program:ti,ab,kw,de,it OR 'clinical trial':it OR 'comparative stud*' OR 'evaluation studies' OR 'statistics'/de OR survey*:ti,ab,kw,de,it OR 'follow-up' OR 'time factors' OR ci:ti,ab,kw,de,it) NOT ('animal'/de NOT 'human'/de OR comment:it OR editorial:it OR review:it OR 'meta analysis':it OR 'case report':ti,ab,kw,de,it OR 'consensus'/de OR guideline:it OR 'history'/de)	7973720
	#13	complication*:ti,ab,kw OR 'complication'/exp/dm_co OR safe*:ti,ab,kw OR 'adverse drug reaction'/exp/dm_co,dm_si OR 'post-operative morbidity':ti,ab,kw OR 'surgical risk'/de OR 'complication'/de OR 'postoperative complication'/de OR 'procedure related':ti,ab,kw	3392434
	#14	injur*:ti,ab,kw OR "airway obstruction$":ti,ab,kw OR 'airway trauma':ti,ab,kw OR anaphyla*:ti,ab,kw OR arrhythmia:ti,ab,kw OR aspiration$:ti,ab,kw OR aware*:ti,ab,kw OR bardicard*:ti,ab,kw OR 'infection*':ti,ab,kw OR "cerebrovascular accident$":ti,ab,kw OR "stroke$":ti,ab,kw OR coma*:ti,ab,kw OR death:ti,ab,kw OR died:ti,ab,kw OR mortality:ti,ab,kw OR delir*:ti,ab,kw OR itiching:ti,ab,kw OR puritus:ti,ab,kw OR "kidney failure$":ti,ab,kw OR "renal failure$":ti,ab,kw OR "organ failure$":ti,ab,kw OR "myocardial infarction$":ti,ab,kw OR	7206158

		'myocardial ischemia':ti,ab,kw OR 'visual loss':ti,ab,kw OR "neurologic deficit$":ti,ab,kw OR 'pneumonia':ti,ab,kw OR nausea:ti,ab,kw OR vomitting:ti,ab,kw OR ponv:ti,ab,kw OR pdnv:ti,ab,kw OR headache:ti,ab,kw OR 'cognitive disfunction*':ti,ab,kw OR edema:ti,ab,kw OR emboli*:ti,ab,kw OR embolus:ti,ab,kw OR "respiratory failure$":ti,ab,kw OR seizure$:ti,ab,kw OR sepsis:ti,ab,kw OR shivering:ti,ab,kw OR 'spinal hematom*':ti,ab,kw OR "cardiac arrest$":ti,ab,kw OR meningitis:ti,ab,kw OR paralys*:ti,ab,kw OR allergy:ti,ab,kw OR allergic*:ti,ab,kw	
	#15	#13 OR #14	9248228
	#16	#1 AND #5 AND #10 AND #12 AND #15	1098
	#17	#9 OR #16	1302
	#18	embase NOT (embase AND medline)	8678944
	#19	'article'/it OR 'article in press'/it OR 'review'/it	30590080
	#20	#17 AND #18	388
	#21	#20 NOT #19	147
CENTRAL (via Cochrane Library)	#1	((knee* OR hip OR hips) NEAR/4 (replacement? OR arthroplasty OR arthroplasties OR implant* OR prosthesis)):ti,ab,kw (Word variations have been searched)	14936
	#2	(tka OR tkr OR uka OR ukr OR thr OR tha):ti,ab,kw	6085
	#3	#1 OR #2	15778
	#4	((anesthesia OR anaesthesia) NEXT/2 (general OR spinal OR regional OR intrathecal OR neuraxial OR conduction)):ti,ab,kw	9080
	#5	("spinal block*" OR "subarachnoid block*" OR "intradural block*" OR "intrathecal block*"):ti,ab,kw	1109
	#6	#4 OR #5	9851
	#7	#3 AND #6 in Trials	474

Search date: 8 February 2022

2.2 General anaesthesia with laryngeal mask in comparison to general anaesthesia with intubation

Table 24 PICO scheme

**P**opulation	People undergoing elective surgery
**I**ntervention	Laryngeal mask
**C**omparison	Intubation
**O**utcome	Perioperative and postoperative complications

Table 25 Inclusion and exclusion criteria

	**Inclusion criteria**
Population	Age ≥ 18 years; elective surgery (at least 80% of the study population)
Intervention	General anaesthesia with respiratory support via a laryngeal mask
Comparison	General anaesthesia with respiratory support via intubation
Outcome	All perioperative or postoperative complications e.g.:<br>Hoarseness, sore throat, voice disorders, injuries in the mouth, injuries to the respiratory tract, aspiration
Study design	Systematic reviews, RCTs, prospective cohort studies
Language	German or English
	**Exclusion criteria**
Intervention	Data collection started before 1990

Table 26 Search strategy for systematic reviews for each database

**Database**	**Search strategy**	**Results**
MEDLINE (via PubMed)	("anesthesia, endotracheal"[MeSH Terms] OR "intubation, intratracheal"[MeSH Terms] OR "Laryngeal Masks"[MeSH Terms] OR "Noninvasive Ventilation"[MeSH Terms] OR (("tracheal"[Title/Abstract] OR "intratracheal"[Title/Abstract] OR "endotracheal"[Title/Abstract]) AND ("intubation*"[Title/Abstract] OR "tube*"[Title/Abstract])) OR ("laryngeal mask*"[Title/Abstract] OR "face mask*"[Title/Abstract] OR "airway management"[Title/Abstract] OR "airway device*"[Title/Abstract])) AND (("general"[Title/Abstract] AND ("anesthesia"[Title/Abstract] OR "anaesthesia"[Title/Abstract])) OR "anesthesia, general"[MeSH Terms]) AND ("meta analysis"[Publication Type] OR "meta analysis"[Title/Abstract] OR "meta analysis as topic"[MeSH Terms] OR "review"[Publication Type] OR "search*"[Title/Abstract])	813
Epistemonikos	(title:((((tracheal OR intratracheal OR endotracheal) AND (intubation* OR tube*)) OR "laryngeal mask" OR "laryngeal masks" OR "face mask" OR "face masks" OR "airway management" OR "airway device" OR "airway devices")) OR abstract:((((tracheal OR intratracheal OR endotracheal) AND (intubation* OR tube*)) OR "laryngeal mask" OR "laryngeal masks" OR "face mask" OR "face masks" OR "airway management" OR "airway device" OR "airway devices")))	81

	AND (title:((general AND (anesthesia OR anaesthesia))) OR abstract:((general AND (anesthesia OR anaesthesia)))) Filter: systematic review	

Search date: 22.10.2021

Table 27 Search strategy for randomised controlled trials for MEDLINE

Database	Search strategy	Results
MEDLINE (via PubMed)	((("anesthesia, endotracheal"[MeSH Terms] OR "intubation, intratracheal"[MeSH Terms] OR "laryngeal masks"[MeSH Terms] OR "noninvasive ventilation"[MeSH Terms] OR (("tracheal"[Title/Abstract] OR "intratracheal"[Title/Abstract] OR "endotracheal"[Title/Abstract]) AND ("tube*"[Title/Abstract] OR "intubation*"[Title/Abstract])) OR ("laryngeal mask*"[Title/Abstract] OR "supraglottic"[Title/Abstract] OR "face mask*"[Title/Abstract] OR "facemask*"[Title/Abstract] OR "airway management"[Title/Abstract] OR "airway device*"[Title/Abstract])) AND ("general anesthesia"[Title/Abstract] OR "general anaesthesia"[Title/Abstract] OR "general anaesthetic*"[Title/Abstract] OR "anesthesia, general"[MeSH Terms]) AND ("randomized controlled trial"[Publication Type] OR "controlled clinical trial"[Publication Type] OR "randomized"[Title/Abstract] OR "randomised"[Title/Abstract] OR "placebo"[Title/Abstract] OR "clinical trials as topic"[MeSH Terms:noexp] OR "randomly"[Title/Abstract] OR "trial"[Title])) NOT ("comment"[Publication Type] OR "letter"[Publication Type] OR "editorial"[Publication Type])) NOT ("animals"[MeSH Terms] NOT "humans"[MeSH Terms])	2816

Search date: 12.11.2021

Table 28 Search strategy for randomised controlled trials for EMBASE

**Database**	**#**	**Search strategy**	**Results**
EMBASE (via Elsevier)	#1	"general an$esthesia":ti,ab,kw OR "general an$esthetic*":ti,ab,kw	88,449
	#2	'general anesthesia'/exp	101,141
	#3	#1 OR #2	135,144
	#4	((tracheal OR endotracheal OR intratracheal) NEAR/3 (tube* OR intubation*)):ti,ab,kw	41,140
	#5	'laryngeal mask*':ti,ab,kw OR supraglottic:ti,ab,kw OR 'face$mask':ti,ab,kw OR 'airway management':ti,ab,kw OR 'airway device*':ti,ab,kw	28,620
	#6	'endotracheal anesthesia'/exp OR 'laryngeal mask'/exp OR 'supraglottic airway device'/exp OR 'mask ventilation'/exp	23,957
	#7	#4 OR #5 OR #6	79,042
	#8	#3 AND #7	10,853
	#9	random*:ti,ab,kw OR placebo*:de,ti,ab,kw OR ((double NEXT/1 blind*):ti,ab,kw)	1,999,776
	#10	#8 AND #9	2,531
	#11	embase NOT (embase AND medline)	8,625,692
	#12	#10 AND #11	836
	#13	'article'/it OR 'article in press'/it OR 'review'/it	30,274,042
	#14	#12 AND #13	658

Search date: 12.11.2021

Table 29 Search strategy for controlled non-randomised trials for each database

Database	#	Search strategy	Results
EMBASE (via Elsevier)	#1	'general an?esthesia':ti,ab,kw OR 'general an?esthetic*':ti,ab,kw	26,891
	#2	'general anesthesia'/exp	103,694
	#3	((tracheal OR endotracheal OR intratracheal) NEAR/3 (tube* OR intubation*)):ti,ab,kw	41,813
	#4	'laryngeal mask*':ti,ab,kw OR supraglottic:ti,ab,kw OR face$mask*:ti,ab,kw OR 'airway management':ti,ab,kw OR 'airway device*':ti,ab,kw	25,282
	#5	'endotracheal anesthesia'/exp OR 'laryngeal mask'/exp OR 'supraglottic airway device'/exp OR 'mask ventilation'/exp	24,305
	#6	#1 OR #2	114,624
	#7	#3 OR #4 OR #5	74,755
	#8	#6 AND #7	9,337
	#9	(cohort OR (cohort AND study) OR (control:ti,ab,kw,de,it AND group*:ti,ab,kw,de,it) OR 'epidemiology'/de OR 'retrospective study'/de OR 'prospective study'/de OR 'longitudinal study'/de OR 'cohort analysis'/de OR 'case control study'/de OR program:ti,ab,kw,de,it OR 'clinical trial':it OR 'comparative stud*' OR 'evaluation studies'/de OR 'statistics'/de OR survey*:ti,ab,kw,de,it OR 'follow-up' OR 'time factors' OR ci:ti,ab,kw,de,it) NOT ('animal'/de NOT 'human'/de OR comment:it OR editorial:it OR review:it OR 'meta analysis':it OR 'case report':ti,ab,kw,de,it OR 'consensus':de OR guideline:it OR 'history'/de)	8,018,056
	#10	complication*:ti,ab,kw OR 'complication'/exp/dm_co OR safe*:ti,ab,kw OR 'adverse drug reaction'/exp/dm_co,dm_si OR 'post-operative morbidity':ti,ab,kw OR 'surgical risk'/de OR 'complication'/de OR 'postoperative complication'/de OR 'procedure related':ti,ab,kw	3,411,771
	#11	aspiration*:ti,ab,kw OR injur*:ti,ab,kw OR ponv*:ti,ab,kw OR nausea*:ti,ab,kw OR vomit*:ti,ab,kw OR 'sore throat*':ti,ab,kw OR dysphagia:ti,ab,kw OR dysphonia:ti,ab,kw OR hoarseness:ti,ab,kw OR cough*:ti,ab,kw OR shivering:ti,ab,kw OR laryngospasm:ti,ab,kw OR bronchospasm:ti,ab,kw OR 'ventilation failure*':ti,ab,kw OR lip:ti,ab,kw OR lips:ti,ab,kw OR tooth:ti,ab,kw OR teeth:ti,ab,kw OR tongue:ti,ab,kw OR lesion*:ti,ab,kw OR vocal*:ti,ab,kw	3,103,928
	#12	'pulmonary aspiration'/exp OR 'postoperative nausea and vomiting'/exp	35,786
	#13	#10 OR #11 OR #12	5,991,286
	#14	#8 AND #9 AND #13	1,722
	#15	embase NOT (embase AND medline)	8,703,170
	#16	#14 AND #15	581
	#17	'article'/it OR 'article in press'/it OR 'review'/it	30,693,348
	#18	#16 AND #17	415

MEDLINE (via PubMed)	#1	"anesthesia, endotracheal"[MeSH Terms] OR "intubation, intratracheal"[MeSH Terms] OR "laryngeal masks"[MeSH Terms] OR "noninvasive ventilation"[MeSH Terms] OR (("tracheal"[Title/Abstract] OR "intratracheal"[Title/Abstract] OR "endotracheal"[Title/Abstract]) AND ("tube*"[Title/Abstract] OR "intubation*"[Title/Abstract])) OR ("laryngeal mask*"[Title/Abstract] OR "supraglottic"[Title/Abstract] OR "face mask*"[Title/Abstract] OR "facemask*"[Title/Abstract] OR "airway management"[Title/Abstract] OR "airway device*"[Title/Abstract])	76,207
	#2	"general anesthesia"[Title/Abstract] OR "general anaesthesia"[Title/Abstract] OR "general anaesthetic*"[Title/Abstract] OR "anesthesia, general"[MeSH Terms]	99,125
	#3	(cohort[all] OR (control[all] AND study[all]) OR (control[tw] AND group*[tw]) OR epidemiologic studies[mh] OR program[tw] OR clinical trial[pt] OR comparative stud*[all] OR evaluation studies[all] OR statistics as topic[mh] OR survey*[tw] OR follow-up*[all] OR time factors[all] OR ci[tw]) NOT ((animals[mh:noexp] NOT humans[mh:noexp]) OR comment[pt] OR editorial[pt] OR review[pt] OR meta analysis[pt] OR case report[tw] OR consensus[mh] OR guideline[pt] OR history[sh])	8,558,928
	#4	"complication*"[Title/Abstract] OR "adverse effects"[MeSH Subheading] OR "safe*"[Title/Abstract] OR "complications"[MeSH Subheading] OR "Postoperative Complications"[MeSH Terms:noexp]	5,768,941
	#5	"aspiration*"[Title/Abstract] OR "injur*"[Title/Abstract] OR "ponv"[Title/Abstract] OR "nausea"[Title/Abstract] OR "vomit*"[Title/Abstract] OR "sore throat*"[Title/Abstract] OR "dysphagia"[Title/Abstract] OR "dysphonia"[Title/Abstract] OR "hoarseness"[Title/Abstract] OR "cough*"[Title/Abstract] OR "shivering"[Title/Abstract] OR "laryngospasm"[Title/Abstract] OR "bronchospasm"[Title/Abstract] OR "ventilation failure*"[Title/Abstract] OR "lip"[Title/Abstract] OR "lips"[Title/Abstract] OR "teeth"[Title/Abstract] OR "tongue*"[Title/Abstract] OR "lesion*"[Title/Abstract] OR "vocal*"[Title/Abstract]	2,263,250
	#6	"Respiratory Aspiration"[Mesh] OR "Pneumonia, Aspiration"[Mesh] OR "Postoperative Nausea and Vomiting"[Mesh]	11,999
	#7	#4 OR #5 OR #6	7,271,255
	#8	#3 AND #7	2,608,656
	#9	#1 AND #2 AND #8	3,269
	#10	(("anesthesia, endotracheal"[MeSH Terms] OR "intubation, intratracheal"[MeSH Terms] OR "laryngeal	2,931

		masks"[MeSH Terms] OR "noninvasive ventilation"[MeSH Terms] OR (("tracheal"[Title/Abstract] OR "intratracheal"[Title/Abstract] OR "endotracheal"[Title/Abstract]) AND ("tube*"[Title/Abstract] OR "intubation*"[Title/Abstract])) OR ("laryngeal mask*"[Title/Abstract] OR "supraglottic"[Title/Abstract] OR "face mask*"[Title/Abstract] OR "facemask*"[Title/Abstract] OR "airway management"[Title/Abstract] OR "airway device*"[Title/Abstract])) AND ("general anesthesia"[Title/Abstract] OR "general anaesthesia"[Title/Abstract] OR "general anaesthetic*"[Title/Abstract] OR "anesthesia, general"[MeSH Terms]) AND ("randomized controlled trial"[Publication Type] OR "controlled clinical trial"[Publication Type] OR "randomized"[Title/Abstract] OR "randomised"[Title/Abstract] OR "placebo"[Title/Abstract] OR "clinical trials as topic"[MeSH Terms:noexp] OR "randomly"[Title/Abstract] OR "trial"[Title])) NOT ("comment"[Publication Type] OR "letter"[Publication Type] OR "editorial"[Publication Type])	
	#11	#9 NOT #10	1,782
CENTRAL (via Cochrane Library)	#1	(tracheal OR intratracheal OR endotracheal):ti,ab,kw (Word variations have been searched)	15202
	#2	(tube* OR intubation*):ti,ab,kw	43692
	#3	#1 AND #2	13317
	#4	("laryngeal mask*" OR supraglottic OR "face mask*" OR facemask* OR "airway management" OR "airway device*"):ti,ab,kw	6544
	#5	#3 OR #4	17539
	#6	("general anaestheisa" OR "general anesthesia" OR "general anaesthetic*"):ti,ab,kw	22206
	#7	#5 AND #6	4626
	#8	(complication* OR safe* OR adverse OR aspiration* OR injur* OR ponv OR nausea OR vomit* OR "sore thorat*" OR dysphagia OR dysphonia OR hoarseness OR cough OR shivering OR laryngospasm OR bronchospasm OR "ventilation failure*" OR lip OR lips OR tongue OR teeth OR tooth OR lesion* OR vocal*):ti,ab,kw	724125
	#9	#7 AND #8 in Trials	2966
	Source:	CINAHL	1066

Search date: 03.03.2022

2.3 Neuraxial anaesthesia with sedation compared to neuraxial anaesthesia with placebo sedation or a form of distraction

Table 30 PICO scheme

**P**opulation	People with elective orthopaedic or plastic surgery on the upper or lower extremities
**I**ntervention	Neuraxial anaesthesia with a single dose and/or continuous administration of an intravenous sedative regardless of the dosage
**C**omparison	Neuraxial anaesthesia with a placebo sedative or a form of distraction (music, video, virtual reality)
**O**utcome	Perioperative and postoperative complications

Table 31 Inclusion and exclusion criteria

	**Inclusion criteria**
Population	Age ≥ 18, elective orthopaedic or plastic surgery on the upper or lower extremities
Intervention	Neuraxial anaesthesia with a single dose and/or continuous administration of an intravenous sedative (e.g. Midazolam, Propofol, Dexmedetomidin, Ketamin, Clonidin (by continuous administration only) regardless of the dosage
Comparison	Neuraxial anaesthesia with a placebo sedative or a form of distraction (e.g. music, video, virtual reality)
Outcome	All occurring perioperative and postoperative complications e.g.:<br>PONV, urinary retention, bradycardia, tachycardia, hypotension, hypertension, itching, delirium, mortality, cardiac complications, pulmonary complications
Study design	RCTs, prospective cohort studies
Language	German or English
	**Exclusion criteria**
Intervention/ Comparison	Intravenous premedication with a sedative (e.g. Midazolam)
Outcome	Postoperative complications with a epidural catheter or PCA

Table 32 Search strategy for randomised controlled trials and controlled non-randomised trials for each database

Database	#	Search strategy	Results
EMBASE (via Elsevier)	#1	(an$esthesia NEXT/2 (regional OR neuraxial OR spinal OR epidural OR intrathecal OR conduction OR 'brachial plexus')):ti,ab,kw	4265
	#2	'spinal block?':ti,ab,kw OR 'subarachnoid block?':ti,ab,kw OR 'intradural block?':ti,ab,kw OR 'intrathecal block?':ti,ab,kw OR 'peripheral nerve block?':ti,ab,kw OR 'supraclavicular block?':ti,ab,kw OR 'axillary block?':ti,ab,kw OR 'infraclavicular block?':ti,ab,kw OR 'interscalene block?':ti,ab,kw	2946
	#3	'spinal anesthesia'/de OR 'epidural anesthesia'/de OR 'brachial plexus anesthesia'/de	56750
	#4	#1 OR #2 OR #3	60485
	#5	hip:ti,ab,kw OR hips:ti,ab,kw OR knee*:ti,ab,kw OR leg:ti,ab,kw OR legs:ti,ab,kw OR femur:ti,ab,kw OR femoral:ti,ab,kw OR tibia:ti,ab,kw OR tibial:ti,ab,kw OR patella:ti,ab,kw OR foot:ti,ab,kw OR feet:ti,ab,kw OR ankle*:ti,ab,kw OR thigh*:ti,ab,kw OR hand*:ti,ab,kw OR arm:ti,ab,kw OR arms:ti,ab,kw OR wrist*:ti,ab,kw OR radius:ti,ab,kw OR radial:ti,ab,kw OR ulna:ti,ab,kw OR finger*:ti,ab,kw OR elbow*:ti,ab,kw OR forearm*:ti,ab,kw OR shoulder*:ti,ab,kw OR scapula:ti,ab,kw OR humerus:ti,ab,kw OR extremit*:ti,ab,kw OR limb*:ti,ab,kw	2784071
	#6	'orthopedic surgery'/exp	571374
	#7	#5 OR #6	3042569
	#8	sedation:ti,ab,kw OR sedative*:ti,ab,kw OR sedated:ti,ab,kw OR propofol:ti,ab,kw OR dexmedetomidine:ti,ab,kw OR midazolam:ti,ab,kw	135643
	#9	'sedation'/exp	82879
	#10	#8 OR #9	164786
	#11	#4 AND #7 AND #10	2084
	#12	random*:ti,ab,kw OR placebo*:de,ti,ab,kw OR ((double NEXT/1 blind*):ti,ab,kw)	2046708
	#13	(cohort OR (cohort AND study) OR (control:ti,ab,kw,de,it AND group*:ti,ab,kw,de,it) OR 'epidemiology'/de OR 'retrospective study'/de OR 'prospective study'/de OR 'longitudinal study'/de OR 'cohort analysis'/de OR 'case control study'/de OR program:ti,ab,kw,de,it OR 'clinical trial':it OR 'comparative stud*' OR 'evaluation studies' OR 'statistics'/de OR survey*:ti,ab,kw,de,it OR 'follow-up' OR 'time factors' OR ci:ti,ab,kw,de,it) NOT ('animal'/de NOT 'human'/de OR comment:it OR editorial:it OR review:it OR 'meta analysis':it OR 'case report':ti,ab,kw,de,it OR 'consensus'/de OR guideline:it OR 'history'/de)	8043455
	#14	#12 OR #13	9180574
	#15	#11 AND #14	1144
	#16	[embase]/lim	28542857
	#17	#15 AND #16	1084
	#18	embase NOT (embase AND medline)	8713682

	#19	#17 AND #18	521
	#20	'article'/it OR 'article in press'/it OR 'review'/it	30744452
	#21	#19 NOT #20	161
MEDLINE (via PubMed)	#1	"regional"[Title/Abstract] OR "neuraxial"[Title/Abstract] OR "spinal"[Title/Abstract] OR "epidural"[Title/Abstract] OR "intrathecal"[Title/Abstract] OR "conduction"[Title/Abstract] OR "brachial plexus"[Title/Abstract]	709,540
	#2	(anesthesia[Title/Abstract]) OR (anaesthesia[Title/Abstract])	241,022
	#3	#1 AND #2	45,485
	#4	((((("spinal block*"[Title/Abstract] OR "subarachnoid block*"[Title/Abstract] OR "intradural block*"[Title/Abstract] OR "intrathecal block*"[Title/Abstract]) OR ("peripheral nerve block*"[Title/Abstract])) OR ("supraclavicular block*"[Title/Abstract])) OR ("axillary block*"[Title/Abstract])) OR ("infraclavicular block*"[Title/Abstract])) OR ("interscalene block*"[Title/Abstract])	5,480
	#5	"anesthesia, spinal"[MeSH Terms] OR "anesthesia, conduction"[MeSH Terms] OR "Anesthesia, Epidural"[Mesh] OR "Brachial Plexus Block"[MeSH]	69,320
	#6	#3 OR #4 OR #5	91,837
	#7	"lower extremity/surgery"[MeSH Terms] OR "upper extremity/surgery"[MeSH Terms] OR "orthopedic procedures"[MeSH Terms] OR "hip"[Title/Abstract] OR "hips"[Title/Abstract] OR "knee*"[Title/Abstract] OR "leg"[Title/Abstract] OR "legs"[Title/Abstract] OR "femur"[Title/Abstract] OR "femoral"[Title/Abstract] OR "tibia"[Title/Abstract] OR "tibial"[Title/Abstract] OR "patella"[Title/Abstract] OR "foot"[Title/Abstract] OR "feet"[Title/Abstract] OR "ankle*"[Title/Abstract] OR "thigh*"[Title/Abstract] OR "hand*"[Title/Abstract] OR "arm"[Title/Abstract] OR "arms"[Title/Abstract] OR "wrist*"[Title/Abstract] OR "radius"[Title/Abstract] OR "radial"[Title/Abstract] OR "ulna"[Title/Abstract] OR "finger*"[Title/Abstract] OR "elbow*"[Title/Abstract] OR "forearm*"[Title/Abstract] OR "shoulder*"[Title/Abstract] OR "scapula"[Title/Abstract] OR "humerus"[Title/Abstract] OR "extremit*"[Title/Abstract] OR "limb*"[Title/Abstract]	2,248,202
	#8	"sedation"[Title/Abstract] OR "sedative*"[Title/Abstract] OR "sedated"[Title/Abstract] OR "propofol"[Title/Abstract] OR "dexmedetomidine"[Title/Abstract] OR "midozolam"[Title/Abstract] OR "deep sedation"[MeSH Terms] OR "conscious sedation"[MeSH Terms] OR "propofol"[MeSH Terms] OR "dexmedetomidine"[MeSH Terms] OR "Midazolam"[Mesh]	92,873
	#9	#6 AND #7 AND #8	1,858
	#10	(randomized controlled trial[pt] OR controlled clinical trial[pt] OR randomized[tiab] OR randomised[tiab] OR placebo[tiab] OR clinical trials as topic[mesh:noexp] OR randomly[tiab] OR trial[ti]) NOT (animals[mh] NOT humans [mh])	1,372,431
	#11	(cohort[all] OR (control[all] AND study[all]) OR (control[tw] AND group*[tw]) OR epidemiologic studies[mh] OR program[tw] OR clinical trial[pt] OR comparative stud*[all] OR evaluation	8,582,068

		studies[all] OR statistics as topic[mh] OR survey*[tw] OR follow-up*[all] OR time factors[all] OR ci[tw]) NOT ((animals[mh:noexp] NOT humans[mh:noexp]) OR comment[pt] OR editorial[pt] OR review[pt] OR meta analysis[pt] OR case report[tw] OR consensus[mh] OR guideline[pt] OR history[sh])	
	#12	#9 AND (#10 OR #11)	1,176
CENTRAL (via Cochrane Library)	#1	((anesthesia OR anaesthesia) NEXT/2 (spinal OR regional OR intrathecal OR neuraxial OR conduction OR epidural OR "brachial plexus")):ti,ab,kw	5769
	#2	("spinal block*" OR "subarachnoid block*" OR "intradural block*" OR "intrathecal block*" OR "peripheral nerve block*" OR "supraclavicular block*" OR "axillary block*" OR "infraclavicular block*" OR "interscalene block*"):ti,ab,kw	2555
	#3	#1 OR #2	7909
	#4	(hip OR hips OR knee* OR leg OR legs OR femur OR femoral OR tibia OR tibial OR patella OR foot OR feet OR ankle* OR thigh* OR hand* OR arm OR arms OR wrist* OR radius OR radial OR ulna OR finger* OR elbow* OR forearm* OR shoulder* OR scapula OR humerus OR extremit* OR limb*):ti,ab,kw	310824
	#5	(sedation OR sedative* OR sedated OR propofol OR dexmedetomidine OR midozolam):ti,ab,kw	38738
	#6	#3 AND #4 AND #5 in Trials	659
	#7	Source: CINAHL	3

Search date: 18.03.2022
