# Supplementary material for: Evidence-based informed consent forms for total knee arthroplasty and anaesthesia: development and pilot study
Source: J Orthop Surg Res. 2026 Feb 5;21:156. doi: 10.1186/s13018-026-06729-z (PMC12930710; doi:10.1186/s13018-026-06729-z)
Supplement: Supplementary file 2 — Supplementary Material 2 [file 13018_2026_6729_MOESM2_ESM.pdf]

Additional file 2: Standardised think-aloud procedure and probing questions

1. Think-Aloud Procedure

Introductory question
What are your expectations of an informed consent form?

Instructions for the Think-Aloud
Please read through the informed consent form in front of you. While doing so, try to think aloud, just as you might when working through something on your own.
It is important that you verbalise everything that comes to mind from the very beginning, no matter how irrelevant it may seem.
Please speak your thoughts as they occur to you.
If necessary, I will remind you to continue thinking aloud.
Thank you for taking part.

2. Follow-up questions after the Think-Aloud

General impressions
What are your overall thoughts on the informed consent form?

Scope and content
How do you assess the length and amount of information in the form?
Did you feel any information was missing? [If yes, which?]
Did you feel there was too much information? [If yes, which parts?]

Structure and clarity
How would you assess the structure of the informed consent form?
Would you change the order of any sections? [If yes, what and why?]
How clear and easy to follow did you find the form overall?

3. In-Depth discussion of specific sections

(These questions are asked when participants raise issues, visible confusion is observed, or difficulties are anticipated. Adjust the specific questions according to the content of the section.)

3a Understanding uncertainty
How would you explain the content of this section to a friend?
What would be your personal takeaway from this part?

3b Benefit tables
What do you think is being presented in the section on [“Conservative treatment”]?

- If answered generally or briefly: What do you think Table 1 is showing?

Did anything in this section cause you difficulty? [If yes, what?]
How would you explain the content of the [highlight box] to a friend?

How did you perceive the clarity of the illustration [“Everyday life before surgery”]?
What do you think of the colour scheme?
Was it easy to read?

How would you explain the message of the illustration [“Everyday life before surgery”] to a friend?
[If the shaded area is not understood: What do you think the shading in the legend represents?]

[If no explanation is possible: What does the number 54 mean to you?]

Let’s now take a look at the section on pain. Please focus on the first two rows in this section.
How would you explain the message of the [“Pain”] section to a friend?

[Understanding of “before and after surgery” comparison: What do you think is the relationship between the first and second column?]
[Understanding of scale where 100 = no pain: Which of the two groups had more pain after surgery? What does the graph in the left column show you?]
[Understanding number of studies and participants: What do the numbers at the bottom right represent?]

[If the table addresses perceptibility of differences: Show Table 10 with adapted instruments)
How would you describe the pain results after 12 and 24 months to a friend?]

[Refer to quality of life at 12 and 24 months: When you compare the text and numbers for quality of life with those for pain, can you identify any differences? How would you explain those differences?]

3c Harm Tables – Conservative Treatment

What do you think Table 2 is showing?

Did you find anything in this section difficult to understand? [If yes, what?]

How would you explain the illustration [“Serious complications”] to a friend?
[If no comment about number of people: Based on your impression, which treatment do you think has a higher risk of serious complications?]
[If no interpretation of numerical values: What does the number 980 mean to you?]

Can you explain why you think no numbers are shown for thrombosis/embolism?

3d Harm Tables – General

What do you think the section [“Conservative treatment”] is about?
If answered generally or briefly: What do you think is the difference between Table 3 and Table 4?

What do you think Table 4 is showing?

Did you find anything in this section difficult to understand? [If yes, what?]

How would you explain the graphic [“Wound complications”] to a friend?
[If no reference to meaning of the numbers: How many people, in your view, experienced no wound complications?]

What does the note “up to 15 years after surgery” mean to you?

3e Understanding of Specific Terms – Dislocation

How would you explain this complication to a friend?
What do you understand by the term “dislocation” or “implant shifting”?

3f Other Possible Complications and Adverse Events

What do you think is being presented in the section [“Other possible complications and adverse events”]?
If answered generally or briefly: How do you think this section differs from the previous one?

4. Final Questions

Thinking about the informed consent form as a whole:

What were your initial expectations? [Refer back to the first question]
Were these expectations met? [If not, why not?]

How would you describe your overall satisfaction with the informed consent form?
