# Supplementary material for: Evidence-based informed consent forms for total knee arthroplasty and anaesthesia: development and pilot study
Source: J Orthop Surg Res. 2026 Feb 5;21:156. doi: 10.1186/s13018-026-06729-z (PMC12930710; doi:10.1186/s13018-026-06729-z)
Supplement: Supplementary file 3 — Supplementary Material 3 [file 13018_2026_6729_MOESM3_ESM.pdf]

Additional file 3: Guiding questions for semi-structured focus group interviews

1. Introduction

You have received two documents from us: an informed consent form about total knee arthroplasty and an accompanying information brochure on the same topic.

- To start with, did you have the opportunity to read through the materials?
- Thinking back to these two documents, what are your initial thoughts?
- What do you think is the main intention behind these materials?
- When you think of an informed consent form, did these documents meet your expectations?

2. Discussion of specific sections

<u>Page 1 – "Dear Patient…"</u>

We are now on the first page, beginning with "Dear Patient…".

- Did anything in particular stand out to you on this first page?

<u>Page 2 – "Progression of knee osteoarthritis"</u>

We are now on page 2, titled "What do I need to know about knee osteoarthritis?"

- What do you think of the two illustrations on this page?
- Further down the page, the progression of knee osteoarthritis is described. What do you take away from this section?
  Or: How would you explain the progression of knee osteoarthritis to a friend?

<u>Page 3 – "About this informed consent form"</u>

On page 3, the section "What should I know about the information in this consent form?" provides background on how the information was developed.

- If a friend asked how this form was developed, what would you tell them?
- If nothing is mentioned: Could you describe what the diagram illustrates in your own words?
  Or: How were the figures presented in the form generated?

<u>Page 4 – "Are there alternatives to surgery?"</u>

We are now on page 4: "Are there alternatives to surgery for treating knee osteoarthritis?"

- What do you think is the purpose of this section?
  If unclear: What treatment options are being compared here?

There is also a grey information box on this page titled "How should the results be interpreted?"

- How would you assess the clarity and layout of the legend?
- Was there anything you didn't understand or found difficult to interpret?

Pages 5–8 – Comparing conservative therapy to TKA (benefits)

We are now looking at the table on page 5 comparing conservative therapy and TKA in terms of everyday activities.

- Which daily activities do you think are being assessed here?
- How would you explain to a friend what the green and blue bars represent—looking at the bars for "Everyday activities before treatment" and those for "12 months after treatment"?
- If not already mentioned: What does the scale below the bars represent? (Expected answer: 100 = full function, no limitation, not percent or people)
- Looking at the second row for "Everyday activities 12 months after treatment": Which treatment appears to offer more benefit?
- How many studies and participants are these results based on?

Follow-up (if anything was unclear in the legend):

- Thinking back to the grey box on page 4, is there anything you now feel was missing or unclear?
- If a friend asked how to interpret these numbers in relation to themselves, how would you respond?
  If uncertain: What are the limitations in transferring these results to individuals?
- If nothing is said: Let's look back at the bottom of page 3—could you explain it again with those sections in mind?

On page 5, there is a text box at the bottom: "Interpretation of the bar graphs"

- Did you find this text box helpful for interpreting the figures?
- Did anything else catch your attention here?

Page 9 – Comparing conservative therapy to TKA (complications)

Now let's look at the table on page 9, showing complications.

- How did you feel reading about the risks and complications?
- What was easy or difficult to understand?
- Why do you think bar graphs were only used for some complications?

Now thinking about both benefits and complications of conservative therapy versus TKA:

- What is the significance of this section for you personally?

Page 10 – "Surgical options for treating knee osteoarthritis"

We are now on page 10, where different surgical procedures and prosthesis types are described.

- Did anything on this page stand out to you?
- Do you find this information useful for preparing for surgery?

Pages 13–18 – “Complications and risks”

Now we turn to pages 13 to 18, which detail possible complications and risks.

- Did anything in the descriptions stand out to you?
- Were there any parts you found difficult to understand?

Page 14 – “Dislocation of the kneecap”

On the bottom of page 14, we see the section on “Dislocation of the kneecap”.

- If a friend asked about the risk of this complication, what would you tell them?
    - How many people are affected by this complication?
    - What does the range 0–114 mean to you?
    - 0–114 out of how many?

If the durability of the prosthesis is personally important to you:

- Do you feel this section gives you sufficient information about that?

Page 20 – “Other possible complications and adverse events”

We are now on page 20.

- Did anything stand out to you in this section?

Page 21 – Before and after the operation

We are now on page 21, covering information on pre- and post-operative care.

- Did anything stand out in this section?
- What about the following page, “Where can I find more information?”—did anything catch your eye?

3. General feedback on the informed consent form

Now we would like to ask you a few general questions about the informed consent form and its structure.

- How would you assess the overall clarity of the document?
- How do you feel about the font size, typeface, and colour?
- What do you think of the colour scheme used throughout?
- We used green-highlighted info boxes on several pages [e.g. page 13 or 21]—what is your impression of those?

5. Final question before moving to the information brochure:

- Before we move on to the information brochure: Is there anything else you would like to mention about the informed consent form? Did you make any notes or have thoughts that have not yet been addressed?

6. Discussion of the information brochure

Now we would like to focus on the information brochure.

- What comes to mind when you think about the brochure?
- In your view, what is the overall purpose or value of the brochure?
- Which pieces of information were particularly helpful to you?
- Were there any pieces of information you felt were missing? What else would you have liked to see included?

<u>Page 11 – "Advantages and disadvantages of different surgical Options"</u>

We are now on page 11.

- Did anything on this page stand out to you?

Let's now look at the grey box titled "How should the results be interpreted?"

- How do you rate the clarity and layout of the legend?
- Was there anything that you found difficult to understand?

<u>Pages 18–21 – Mobile vs fixed-bearing prostheses</u>

Let's now turn to the section comparing mobile and fixed-bearing prostheses on pages 18 to 21. Focusing on page 18 and the domain of everyday activities:

- How would you explain the information in this section to a friend?

<u>Page 22 – Cemented vs uncemented prosthesis</u>

We are now on page 22.

- What information do you take away from this section?

<u>Pages 31–32 – General information texts</u>

Now we'd like to discuss the general information texts on pages 31 and 32.

- Did anything in either of these sections stand out to you?

7. Final question

- Is there anything else you would like to mention or discuss that hasn't yet been addressed?
