# Supplementary material for: Evidence-based informed consent forms for total knee arthroplasty and anaesthesia: development and pilot study
Source: J Orthop Surg Res. 2026 Feb 5;21:156. doi: 10.1186/s13018-026-06729-z (PMC12930710; doi:10.1186/s13018-026-06729-z)
Supplement: Supplementary file 4 — Supplementary Material 4 [file 13018_2026_6729_MOESM4_ESM.pdf]

German version of the informed consent forms for TKA and anaesthesia

MARTIN-LUTHER-UNIVERSITÄT HALLE-WITTENBERG

universität Witten/Herdecke

Künstliches Kniegelenk

Aufklärungsbogen für den Einbau einer Knie-Total-Endoprothese (Knie-TEP)

Klinikdruck/Stempel

Patientendaten

Sehr geehrte Patientin, sehr geehrter Patient,

bei Ihnen wurde eine Kniearthrose festgestellt. Sie erwägen, den von der Arthrose betroffenen Teil des Knies durch eine Total-Endoprothese ersetzen zu lassen. Dieser Aufklärungsbogen soll Sie bei einer informierten Entscheidung unterstützen und auf das Aufklärungsgespräch vorbereiten. Bringen Sie diesen Aufklärungsbogen deshalb bitte auch zum Aufklärungsgespräch mit.

An wen richtet sich der Aufklärungsbogen?

Die folgenden Informationen richten sich an Patientinnen und Patienten mit Kniearthrose (Gonarthrose). Wenn Sie unter schweren Fehlstellungen oder Verletzungen nahe dem Kniegelenk leiden, oder bei Ihnen eine operative Korrektur von Fehlstellungen erfolgte, erhalten Sie vom Behandlungsteam Informationen zu Ihrer individuellen Situation. Diese Informationen gelten nicht, wenn bei Ihnen ein künstliches Kniegelenk ausgetauscht werden soll oder wenn Sie Rheuma (rheumatoide Arthritis) haben.

Inhalt

Was sollte ich zur Kniearthrose wissen?	2
Was sollte ich zu den Informationen in diesem Aufklärungsbogen wissen?	3
Gibt es außer einer Operation noch andere Möglichkeiten, die Kniearthrose zu behandeln?	4-9
Welche Möglichkeiten gibt es, die Kniearthrose mit einem künstlichen Gelenk zu behandeln?	10
Einbau einer Total-Endoprothese: Wie unterscheiden sich die verschiedenen Möglichkeiten?	11-12
Welche Komplikationen und Risiken können auftreten?	13-20
Was gibt es vor dem Eingriff zu beachten?	21
Was gibt es nach dem Eingriff zu beachten?	21
Wo finde ich weitere Informationen?	22
Wer hat den Aufklärungsbogen mit welchen Quellen, wie erstellt?	22
Wichtige Fragen zu Ihrem Gesundheitszustand	23-24
Anmerkungen zum Aufklärungsgespräch	25
Einwilligung	26

Was sollte ich zur Kniearthrose wissen? [1]

Um zu verstehen, was bei Arthrose passiert, ist es hilfreich, den Aufbau eines menschlichen Knies anzusehen. Der Oberschenkelknochen, das Schienbein und die Kniescheibe bilden zusammen das Kniegelenk. Die Knochen sind an den Flächen, die in Kontakt zu anderen Knochen stehen, mit einer Knorpelschicht überzogen. Die Knorpelschicht dient dem Schutz des Knochens. Zwischen den Knorpeln von Oberschenkel und Schienbein liegen die Menisken. Sie dienen als „Stoßdämpfer“. Außerdem gleichen sie durch ihre anpassungsfähige Struktur die natürlichen Unebenheiten der Knorpel aus und sorgen so für eine reibungsarme Bewegung. Das Außen- und Innenband sowie das vordere und hintere Kreuzband stabilisieren das Kniegelenk während der Bewegung.

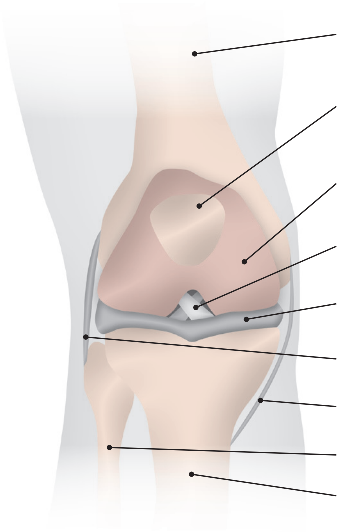

Abbildung 1: Gesundes Knie (frontal)

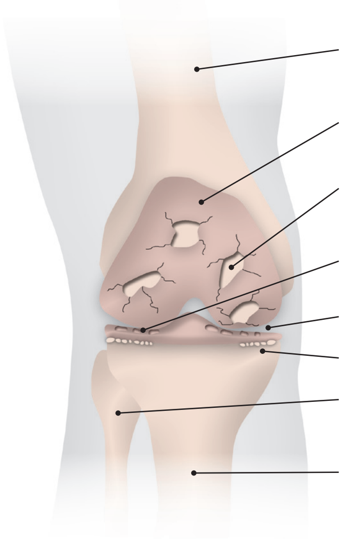

Abbildung 2: Knie mit Arthrose (frontal)

Bei einer Kniearthrose kommt es zu einem Abbau der Gelenkknorpel im Kniegelenk. Kniearthrose betrifft im Verlauf aber auch andere Strukturen des Kniegelenks: die Knochen, die Bänder, die Menisken und die an das Gelenk angrenzenden Muskeln. Die Knorpel können sich nur schlecht selbst mit Nährstoffen versorgen. Sie verfügen nicht über eine ausreichende Blutzufuhr. Eine wichtige Funktion bei der Versorgung des Knorpels übernehmen deshalb die Menisken, die Gelenkschleimhaut und die Gelenkflüssigkeit. Sie versorgen die Knorpel mit wichtigen Nährstoffen. Dies geschieht über Zug und Druck – also wenn Sie sich bewegen. Aus diesem Grund ist Bewegung auch so wichtig für das Kniegelenk.

Wie kann eine leichte bis mittelschwere Kniearthrose ohne Behandlung verlaufen?

Wie eine Kniearthrose bei einem Menschen verläuft, lässt sich nicht genau vorhersagen. Um den Nutzen möglicher Behandlungen beurteilen zu können, ist es hilfreich, den natürlichen Verlauf der Erkrankung ohne Behandlung zu betrachten. Eine Zusammenfassung von 6 Studien untersuchte, wie sich Schmerzen bei einer Kniearthrose über die Zeit entwickeln. An den Studien nahmen 7000 Menschen mit leichter bis mittelschwerer Kniearthrose teil. Die Teilnehmenden erhielten keine strukturierte Behandlung, nahmen aber vielleicht bei Bedarf Schmerzmittel ein. Über einen Zeitraum von **5 bis 8 Jahren** zeigte sich:

- **Bei etwa 85 von 100 Personen haben sich die Schmerzen in diesem Zeitraum weder verschlimmert noch verbessert.**
- **Bei etwa 7 von 100 Personen nahmen die Schmerzen in diesem Zeitraum zu.**
- **Bei etwa 8 von 100 Personen ließen die Schmerzen in diesem Zeitraum nach.**

Was sollte ich zu den Informationen in diesem Aufklärungsbogen wissen?

Dieser Aufklärungsbogen wurde mit den Methoden der evidenzbasierten Medizin entwickelt. Hierbei werden die derzeit besten verfügbaren Studien als Informationsquelle genutzt.

Woher weiß man, ob eine Behandlung besser ist als eine andere?

Um herauszufinden, ob eine Behandlung besser ist als eine andere, werden sogenannte randomisiert-kontrollierte Studien durchgeführt. Wie eine solche Studie funktioniert und warum diese Art von Studien zum Nachweis der Wirksamkeit so wichtig ist, möchten wir Ihnen am folgenden Beispiel zeigen.

Kann der Einsatz einer Teil-Endoprothese die Schmerzen von Personen mit Kniearthrose besser senken als der Einsatz einer Total-Endoprothese?

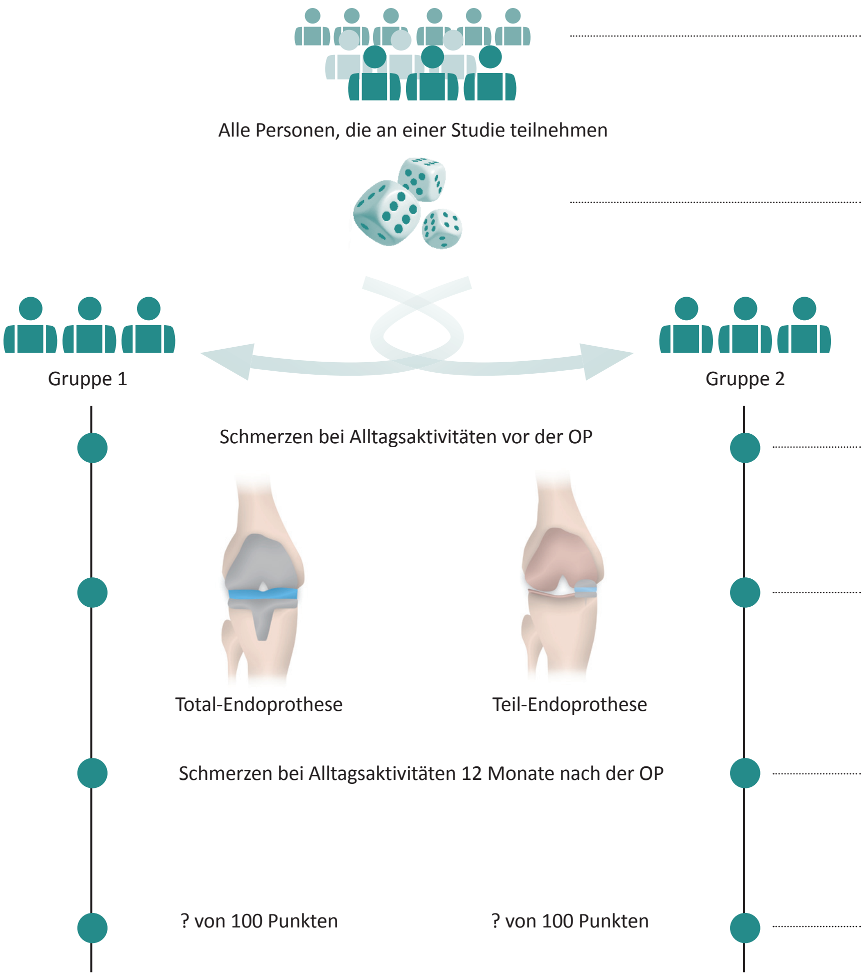

Für aussagekräftige Ergebnisse ist es nötig, dass ausreichend viele Personen an der Studie teilnehmen.

Eine zufällige Zuteilung der teilnehmenden Personen zu den Gruppen sorgt dafür, dass alle Eigenschaften (z.B. Alter und Gewicht) der Personen ungefähr gleich verteilt sind. Die Gruppen sind dadurch vergleichbar.

Alle Personen füllen einen Fragebogen zu ihren Schmerzen aus.

Durchführung der Operation

Zu einem festgelegten Zeitraum sollten möglichst alle Personen erneut den Fragebogen zu ihren Schmerzen ausfüllen.

Bei der Auswertung wird verglichen, ob es einen Unterschied zwischen Gruppe 1 und Gruppe 2 gibt. Nur so kann verlässlich gesagt werden, ob der Einsatz einer Teil-Endoprothese die Schmerzen besser senken kann als der Einsatz einer Total-Endoprothese.

Abbildung 3: Beispiel einer randomisiert-kontrollierten Studie

Welche Sicherheiten und Unsicherheiten sind mit den Zahlen verbunden?

Zahlen vermitteln den Eindruck von Genauigkeit. Tatsächlich sind sie mit vielen Unsicherheiten verbunden. Zahlen als Ergebnisse wissenschaftlicher Studien sind nur Schätzwerte. Wie genau die Zahlen geschätzt werden, hängt zum Beispiel davon ab, wie groß eine Studie ist. Außerdem handelt es sich um Wahrscheinlichkeiten. Für die einzelne Person lassen sich keine sicheren Vorhersagen treffen.

Sind die Zahlen im Aufklärungsbogen vollständig auf mich übertragbar?

An den Studien, die für die Erstellung des Aufklärungsbogens verwendet wurden, nahmen auch Menschen teil, die sich hinsichtlich der Erkrankung und möglicher Nebenerkrankungen von der Zielgruppe dieser Information unterscheiden. Es ist daher möglich, dass sich die Ergebnisse nicht vollständig auf Sie übertragen lassen. Wenn Sie wissen wollen, ob sich die Studienergebnisse auf Ihre Situation übertragen lassen, sprechen Sie bitte mit Ihrer Ärztin oder Ihrem Arzt darüber.

Gibt es außer einer Operation noch andere Möglichkeiten, die Kniearthrose zu behandeln?

Vielleicht fragen Sie sich, ob andere Behandlungsformen genauso gut helfen würden wie eine Operation, bei der Sie ein künstliches Kniegelenk erhalten. Im folgenden Abschnitt können Sie sich ein Bild davon machen, was eine konservative Behandlung im Vergleich zu einer Operation nützt und zu welchen Komplikationen es bei den Optionen kommen kann.

Die dargestellten Ergebnisse beruhen auf einer Studie mit 100 Personen. Diese verglich den Einbau einer Total-Endoprothese mit einem optimalen konservativen Behandlungsprogramm. Das konservative Programm bestand aus verschiedenen Einzelmaßnahmen. Dazu zählten Physiotherapie, Schulungen zum Umgang mit Arthrose, Gewichtsreduktion, orthopädische Hilfsmittel (z.B. Schuheinlagen) und Schmerzmittel.

> **i** Zahlen sind Schätzungen von Wahrscheinlichkeiten und lassen keine konkreten Vorhersagen für eine Person zu. Fragen Sie Ihr Behandlungsteam, wenn Sie sich unsicher sind, ob sich die Ergebnisse auf Ihre Situation übertragen lassen.

Welchen Nutzen kann ich von einer konservativen Behandlung im Vergleich zu einer Total-Endoprothese (Knie-TEP) erwarten? [2]

Wie können die Ergebnisse gelesen werden?

Die Teilnehmenden füllten vor und nach der Behandlung einen Fragebogen zu verschiedenen Lebensbereichen aus. Die folgende Abbildung hilft Ihnen, die dargestellten Ergebnisse zu verstehen.

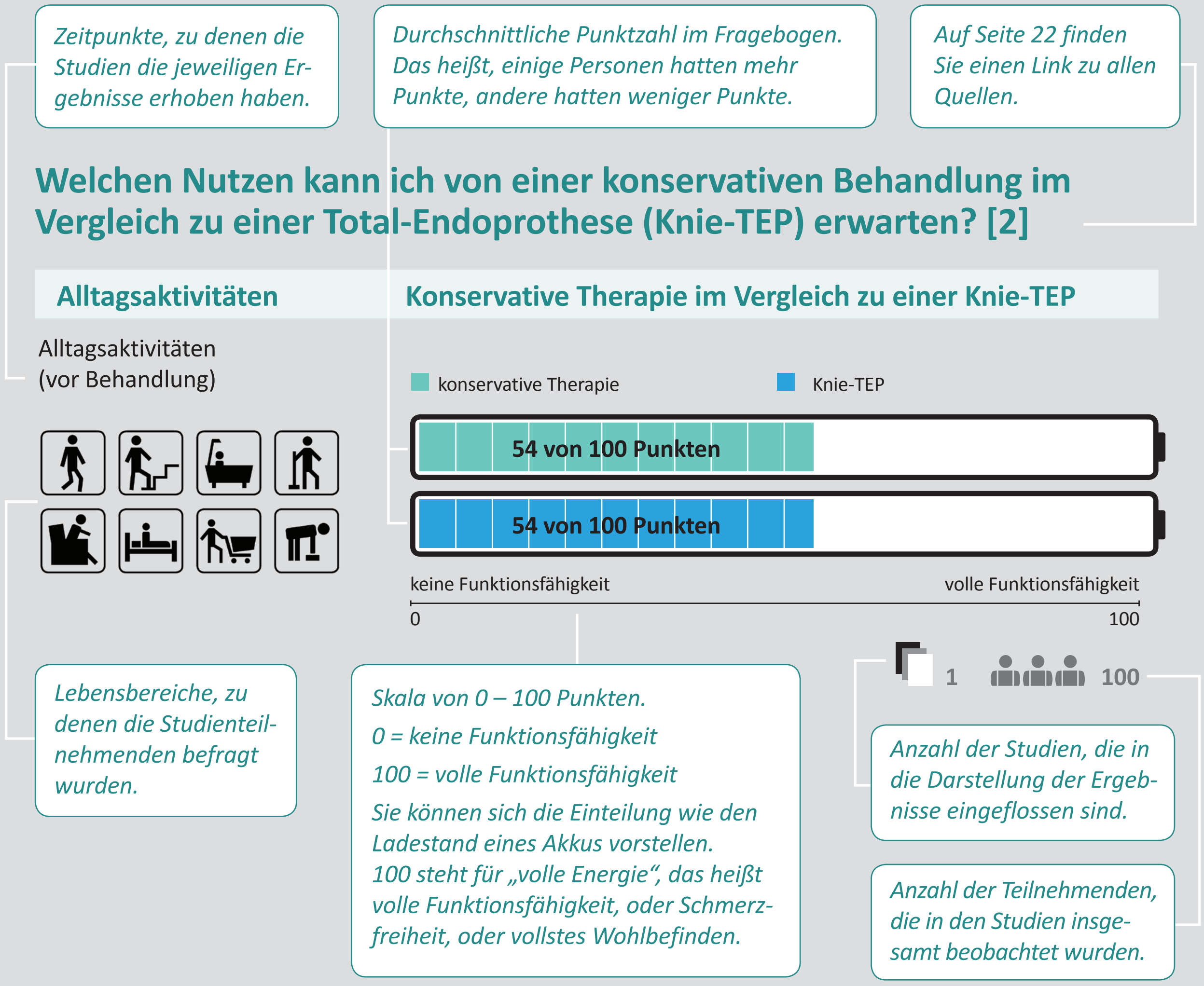

Alltagsaktivitäten

Konservative Therapie im Vergleich zu einer Knie-TEP

Alltagsaktivitäten
(vor Behandlung)

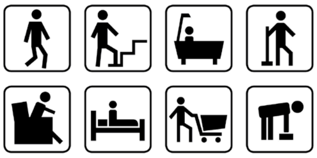

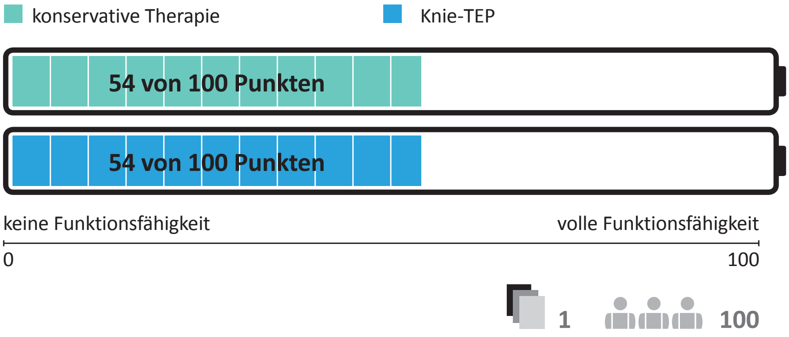

Alltagsaktivitäten
(12 Monate nach Behandlung)

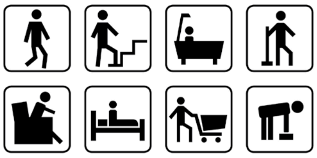

bessere Funktionsfähigkeit mit beiden Verfahren
größere Verbesserung mit Knie-TEP

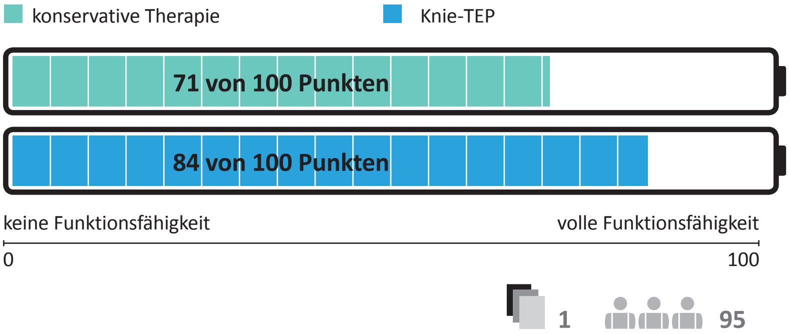

Interpretation der Balkendiagramme

Mithilfe eines Fragebogens wurde die Funktionsfähigkeit in verschiedenen Alltagsaktivitäten vor der Behandlung und 12 Monate nach der Behandlung erhoben.

Vor der Behandlung haben sowohl die Personen, die eine konservative Therapie erhalten werden, als auch die Personen, die eine Knie-TEP erhalten werden, ihre Funktionsfähigkeit in Alltagsaktivitäten durchschnittlich mit 54 von 100 Punkten bewertet. Dabei bedeuten 0 keine Funktionsfähigkeit und 100 volle Funktionsfähigkeit.

12 Monate nach der Behandlung bewerteten die Teilnehmenden, die eine konservative Therapie erhielten, ihre Funktionsfähigkeit in Alltagsaktivitäten durchschnittlich mit 71 von 100 Punkten. Teilnehmende, die eine Knie-TEP erhalten haben, bewerteten ihre Funktionsfähigkeit durchschnittlich mit 84 von 100 Punkten.

Im Vergleich zum Wert vor der Behandlung (54), gaben sowohl Teilnehmende mit konservativer Therapie (71) als auch Teilnehmende mit einer Knie-TEP (84) eine bessere Funktionsfähigkeit in Alltagsaktivitäten nach der Behandlung an. Bei Teilnehmenden mit einer Knie-TEP war die Verbesserung im Vergleich zu vor der Behandlung größer.

Diese Ergebnisse basieren auf 1 Studie mit 100 Teilnehmenden. Nach 12 Monaten haben noch 95 der ursprünglich 100 Teilnehmenden den Fragebogen ausgefüllt.

Schmerzen | Konservative Therapie im Vergleich zu einer Knie-TEP

Schmerzen
(vor Behandlung)

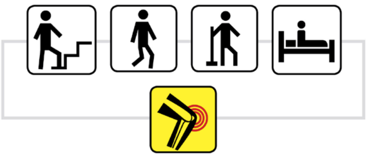

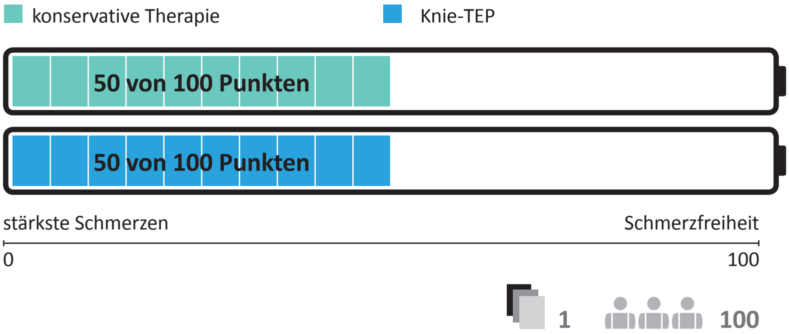

Schmerzen
(12 Monate nach Behandlung)

schmerzfreier mit beiden Therapieoptionen
größere Verbesserung mit Knie-TEP

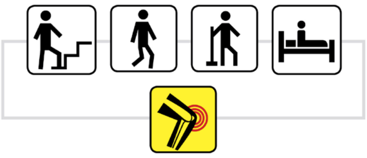

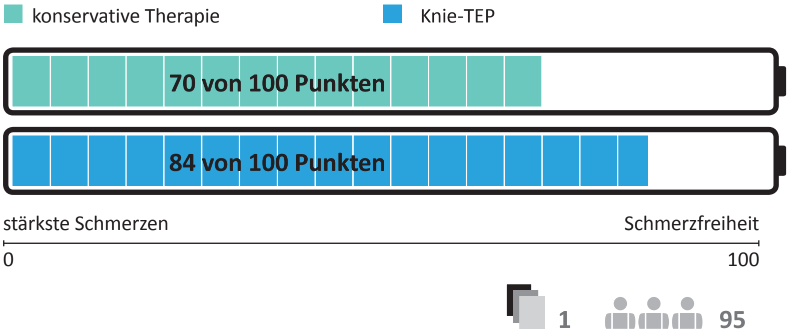

Lebensqualität | Konservative Therapie im Vergleich zu einer Knie-TEP

Lebensqualität
(vor Behandlung)

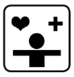

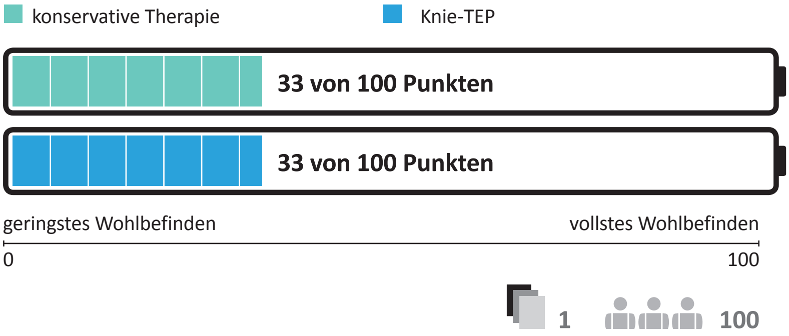

Lebensqualität
(12 Monate nach Behandlung)

höheres Wohlbefinden mit beiden Therapieoptionen
größere Verbesserung mit Knie-TEP

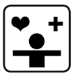

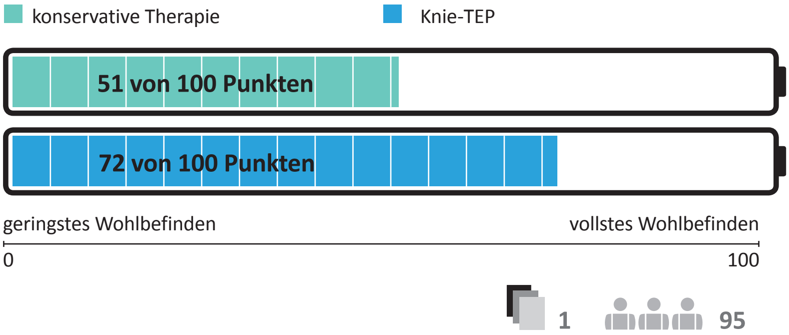

Nutzen	Konservative Therapie im Vergleich zu einer Knie-TEP

Sportliche Aktivität
(vor Behandlung)

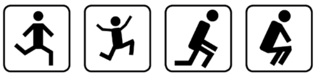

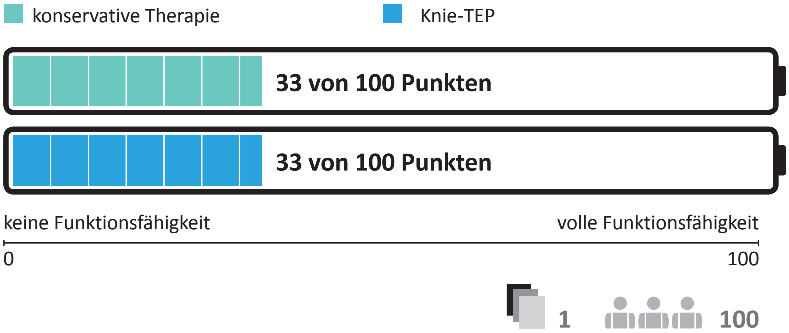

Sportliche Aktivität
(12 Monate nach Behandlung)

bessere Funktionsfähigkeit mit beiden Therapieoptionen
größere Verbesserung mit Knie-TEP

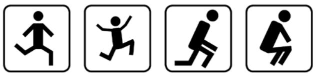

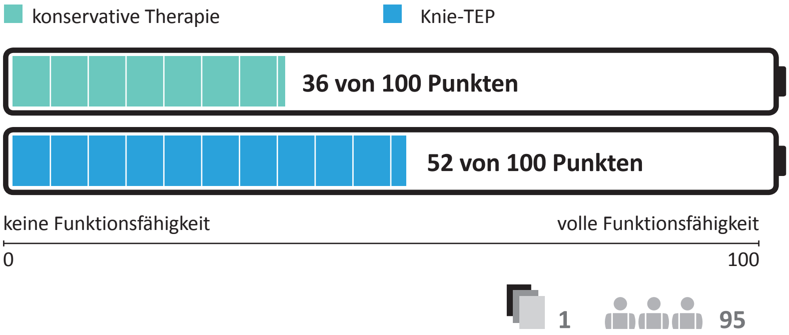

Ihre Fragen/Notizen

Welche Komplikationen können bei einer konservativen Behandlung im Vergleich zu einer Total-Endoprothese (Knie-TEP) auftreten? [2]

Wie können die Ergbnisse gelesen werden?

Die Studie berichtet alle aufgetretenen Komplikationen bei der konservativen Therapie und der Knie-TEP. Es nahmen wenige Menschen an der Studie teil. Deshalb sind nur für Komplikationen Zahlen angegeben, die eine weitere Behandlung zur Folge hatten. Die folgende Abbildung hilft Ihnen, die dargestellten Ergebnisse zu verstehen.

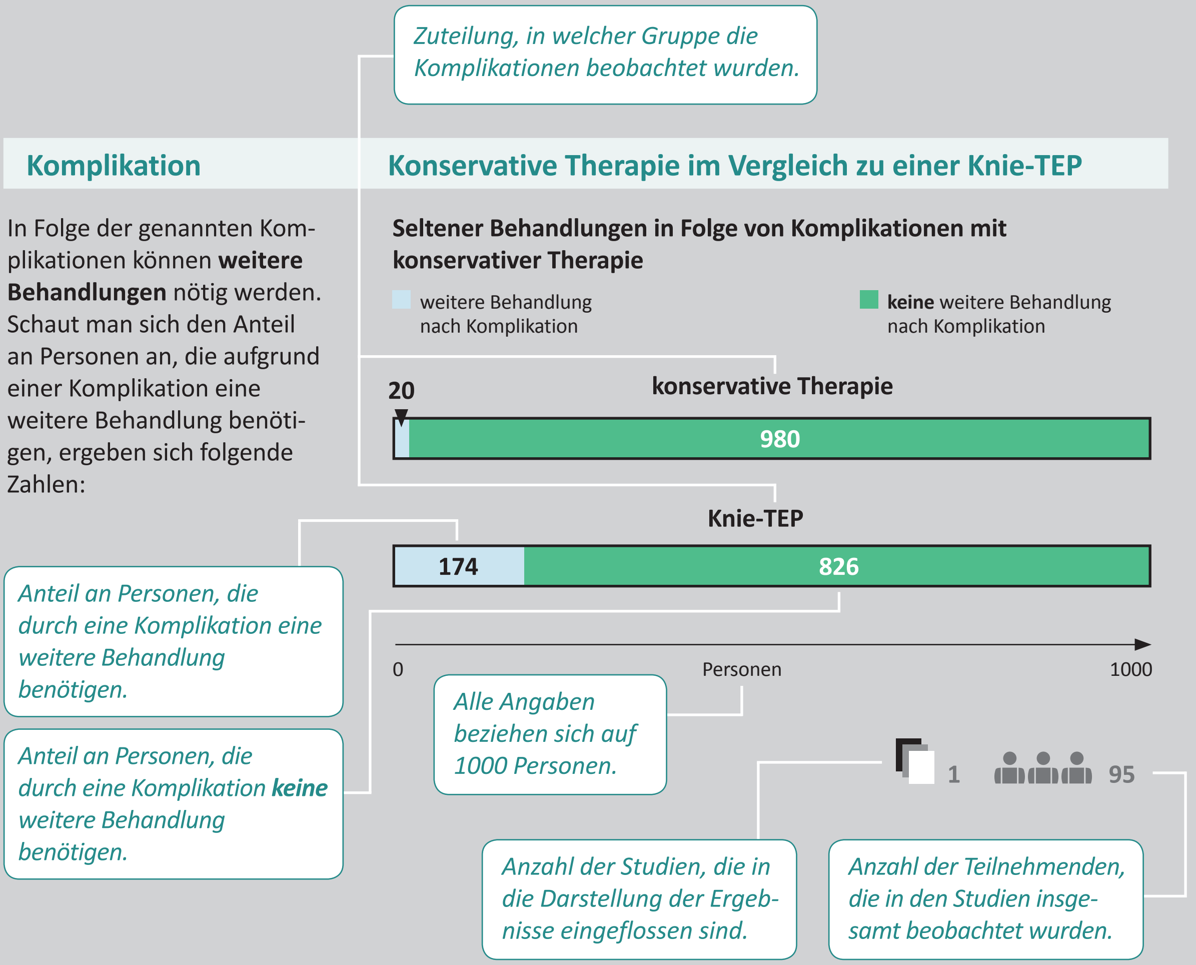

Komplikation	Konservative Therapie im Vergleich zu einer Knie-TEP
**Thrombose / Embolie**<br>Ein Blutgerinnsel kann den Blutstrom im Blutgefäß behindern und stoppen. Werden Blutgefäße verschlossen, kann das lebensgefährliche Folgen haben (z.B. Beinvenenthrombose, Lungenembolie, Herzinfarkt, Schlaganfall).<br>Durch das Einsetzen der Prothese können Fettpartikel in den Blutstrom gelangen und ähnliche Folgen hervorrufen.	**Hinweis auf kleineren Anteil an Thrombosen mit konservativer Therapie**<br>1 95
**Instabilität**<br>kann durch eine unausgeglichene Spannung des Bandapparates entstehen und äußert sich beispielsweise durch Bewegungseinschränkungen. Eine erneute Operation kann nötig werden.	**Hinweis auf größeren Anteil an Instabilitäten mit konservativer Therapie**<br>1 95
Ein **eingeschränkter Bewegungsumfang (Steifigkeit)** kann sowohl die Beugung als auch die Streckung des Knies betreffen und kann verschiedene Ursachen haben. Neben konservativen Behandlungsmöglichkeiten kann auch eine operative Behandlung notwendig werden.	**Hinweis auf kleineren Anteil an Steifigkeit mit konservativer Therapie**<br>1 95
**Knochenbrüche**<br>können den Oberschenkelknochen, das Schienbein oder die Kniescheibe betreffen.	**Hinweis auf kleineren Anteil an Knochenbrüchen mit konservativer Therapie**<br>1 95
**Gelenkinfektionen**<br>können schwerwiegende Folgen haben und sind mit erheblichen Bewegungseinschränkungen verbunden. Es können langwierige Behandlungen und ein Austausch der Prothese nötig werden.	**Hinweis auf kleineren Anteil an Gelenkinfektionen mit konservativer Therapie**<br>1 95
Infolge der genannten Komplikationen können **weitere Behandlungen** nötig werden. Schaut man sich den Anteil an Personen an, die aufgrund einer Komplikation eine weitere Behandlung benötigen, ergeben sich folgende Zahlen:	**seltener Behandlungen in Folge von Komplikationen mit konservativer Therapie**<br>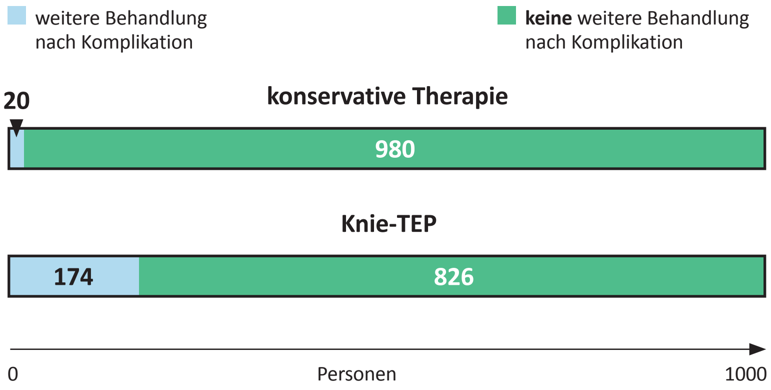<br>1 95

Wie kann mit einem künstlichen Kniegelenk eine Kniearthrose behandelt werden?

Nicht immer ist das gesamte Kniegelenk von Arthrose betroffen. Abhängig vom Ausmaß der Arthrose gibt es zwei Arten von Prothesen, die sich darin unterscheiden, welchen Anteil der Gelenkfläche sie ersetzen. Welche Prothese eingebaut werden kann, hängt von verschiedenen Faktoren ab.

i

Wenn Sie mehr über die Vor- und Nachteile wissen möchten, können Sie sich in der beiliegenden Informationsbroschüre auf den Seiten 11-30 dazu informieren.

Teil-Endoprothese [1]

Sind die Bänder des Knies intakt und ist nur ein Teil des Knies von Arthrose betroffen, kann eine Teil-Endoprothese eingesetzt werden. Sie wird auch Schlittenprothese genannt. Bei dieser Form der Endoprothese werden ausschließlich die betroffenen Gelenkflächen des Oberschenkelknochens sowie des Schienbeins entfernt und durch Metallkomponenten ersetzt. Der gesunde Bereich des Knies bleibt erhalten. Der Eingriff kann unter verschiedenen Anästhesieverfahren erfolgen. Die Aufklärung über die Anästhesie erfolgt gesondert.

Anders als bei einer Total-Endoprothese, gibt es für den Einbau einer Teil-Endoprothese keine erforderliche Mindestanzahl an Operationen. Sie können die Erfahrung Ihrer Ärztin oder Ihres Arztes mit Teilprothesen erfragen.

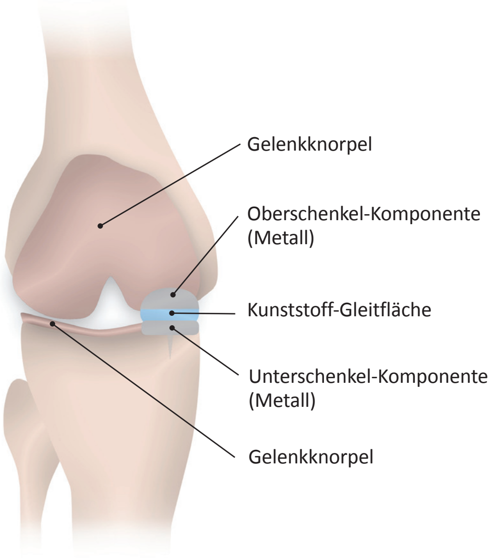

Abbildung 5: Teil-Endoprothese (frontal)

Total-Endoprothese [1]

Bei einer Total-Endoprothese werden die gesamten Gelenkflächen des Oberschenkelknochens und des Schienbeins ersetzt. Eine Total-Endoprothese besteht aus einer oberen und einer unteren Komponente aus Metall. Zwischen diesen Metallkomponenten wird bei den meisten Prothesen eine Gleitfläche aus Kunststoff gesetzt. Für die Total-Endoprothese stehen Prothesentypen mit verschiedenen Eigenschaften zur Verfügung. Im nächsten Abschnitt erhalten Sie Informationen dazu. Der Eingriff kann unter verschiedenen Anästhesieverfahren erfolgen. Die Aufklärung über die Anästhesie erfolgt gesondert.

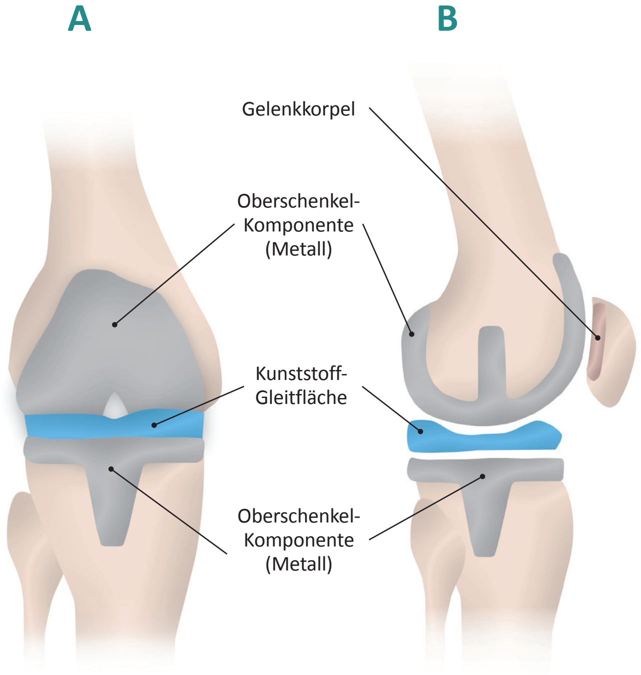

Abbildung 6:
(A) Total-Endoprothese (frontal)
(B) Total-Endoprothese (seitlich)

Einbau einer Total-Endoprothese: Wie unterscheiden sich die verschiedenen Möglichkeiten?

Vielleicht haben Sie schon von den verschiedenen Möglichkeiten gehört, die für den Einbau einer Total-Endoprothese angeboten werden. Es gibt verschiedene Möglichkeiten, sowohl was die Art der Prothese als auch was die Verankerungsmethode oder das Operationsverfahren betrifft. Im Folgenden erhalten Sie einen Überblick zu den verschiedenen Möglichkeiten. Welche für Sie individuell in Frage kommen kann, wird Ihr Behandlungsteam mit Ihnen im Detail besprechen. Es kann auch während der Operation notwendig werden, von der ursprünglich geplanten Vorgehensweise abzuweichen.

i

Wenn Sie mehr über die Vor- und Nachteile wissen möchten, können Sie sich in der beiliegenden Informationsbroschüre auf den Seiten 5-9 und 11-30 dazu informieren. Hier finden Sie auch vereinfachte Abbildungen zu den verschiedenen Möglichkeiten.

Welche verschiedenen Typen von Total-Endoprothesen gibt es?

Total-Endoprothese mit verschiedenen Kopplungsgraden [1]

Bei der Wahl der Prothesenart stehen verschiedene Kopplungsgrade zur Verfügung. Der Begriff „Kopplungsgrad“ steht dafür, in welchem Umfang die obere und untere Metallkomponente miteinander verbunden sind. Wie hoch der Grad der Kopplung sein muss, hängt von mehreren Faktoren ab. Beispielsweise davon, wie stabil die Bänder sind. Sind alle Bänder noch ausreichend intakt, kann eine ungekoppelte Prothese eingebaut werden. In diesem Fall wird das künstliche Kniegelenk durch den natürlichen Bandapparat gestützt. Bei unzureichender Stabilität der Bänder kann eine stabilisierte Prothese infrage kommen. Hierbei wird das hintere Kreuzband ersetzt und eine Kunststoffgleitfläche mit einem Zapfen verwendet, der das künstliche Kniegelenk stabilisiert. Bei schweren Bandschäden können Prothesen mit einem höheren Kopplungsgrad notwendig werden. Bei einer gekoppelten Prothese sind beide Metallkomponenten mit einer Art Scharnier verbunden.

Total-Endoprothese mit oder ohne Ersatz der Kniescheibenrückseite [1]

Die Rückseite der Kniescheibe ist ebenfalls von Knorpel überzogen. Auch dieser kann von der Arthrose betroffen sein. Wenn dies der Fall ist, kann er ebenfalls durch eine künstliche Gelenkfläche ersetzt werden.

Total-Endoprothese mit unbeweglicher oder beweglicher Kunststoffgleitfläche [1]

Bei der Art der Kunststoffgleitfläche zwischen den Metallkomponenten können zwei Methoden unterschieden werden. Bei der einen Methode wird die Gleitfläche unbeweglich an der unteren Metallkomponente fixiert. Bei der anderen wird die Gleitfläche beweglich an der unteren Metallkomponente befestigt. Damit soll die Roll-Gleitbewegung eines menschlichen Kniegelenks nachempfunden werden.

Welche verschiedenen Verankerungsmethoden gibt es?

Total-Endoprothese mit oder ohne zementierte Befestigung [1]

Es gibt verschiedene Methoden, wie die Prothese am Knochen befestigt werden kann. Zementfrei eingebaute Prothesenteile werden auf den Knochen gepresst und verwachsen durch ihre raue Oberfläche dauerhaft mit dem Knochen. Bei einer zementierten Prothese kommt spezieller Knochenzement zum Einsatz. Es können beide Teile oder nur ein Teil der Prothese so befestigt werden.

Welche Operationsverfahren gibt es?

Total-Endoprothese mit oder ohne computergestützter Navigation [1]

Durch eine computergestützte Navigation während der Operation soll das Ausrichten der Prothese entlang der Beinachse unterstützt werden. Anhand von Fixpunkten berechnet ein Computerprogramm die optimale Position der Prothesenteile. Die Operation selbst führt die Ärztin oder der Arzt durch.

Total-Endoprothese mit oder ohne patientenindividuelle Instrumente [1]

Um die Prothesenteile optimal an den Knochen anzupassen, werden die Knochenenden durch Zuschnitte vorbereitet. Dazu werden „Schnittblöcke“ an den Gelenkflächen befestigt. Diese dienen als Schablone für die Zuschnitte. Die Schnittblöcke können entweder eine Standardform haben oder auf Patientinnen und Patienten individuell angepasst werden. Dies geschieht anhand von CT- oder MRT-Aufnahmen des Kniegelenkes.

Total-Endoprothese mit oder ohne minimalinvasiven Zugang [1]

Um das Kniegelenk unter den Hautschichten freizulegen, gibt es verschiedene Methoden. Neben einem normalen Schnitt, kann auch ein minimalinvasiver Schnitt infrage kommen. Der Hautschnitt und die zurückbleibende Narbe sind hierbei kleiner. Allerdings ist die Übersicht innerhalb des Gelenks mit kleinerem Schnitt geringer.

Total-Endoprothese mit oder ohne robotergestützte Navigation [1]

Die robotergestützte Navigation wurde entwickelt, um Prothesenteile genauer an der Beinachse ausrichten zu können. Es werden verschiedene Robotersysteme zum Einbau künstlicher Kniegelenke eingesetzt. Es gibt Roboter-Arme, die unter Kontrolle eigenständig operieren oder vom Operierenden gesteuert werden. Außerdem gibt es Roboter, die nur Informationen liefern während der Operation. Ob robotergestützte Navigation in Ihrer Klinik eingesetzt wird, erfahren Sie von Ihrer behandelnden Ärztin oder ihrem behandelnden Arzt.

Disziplinübergreifendes Therapie- / Behandlungskonzept

Total-Endoprothese mit oder ohne „Fast Track“ [1]

Das „Fast-Track“-Verfahren ist ein Behandlungs- und Therapiekonzept, bei dem verschiedene medizinische Berufe eng zusammenarbeiten. Ziel ist es, die Ergebnisse einer Operation für Patientinnen und Patienten zu verbessern.. Was genau in dem Konzept eingesetzt wird, kann sich von Klinik zu Klinik unterscheiden. Bisher gibt es dazu in Deutschland keinen Standard. Aus diesem Grund kann der erwartbare Nutzen solcher Konzepte nicht eingeschätzt werden.

Welche weiteren Maßnahmen können Bestandteil der Operation sein? [1]

Je nach Ihrer individuellen Situation kann es sein, dass vor, während oder nach dem Einbau der Total-Endoprothese weitere Maßnahmen durchgeführt werden. Das können zum Beispiel Röntgenaufnahmen vor und nach der Operation sein. Weiterhin kann Ihnen während der Operation ein Medikament (Tranexamsäure) verabreicht werden, das den Blutverlust verringern soll. Kurzfristiges Ablösen von Knochenstrukturen kann zum Beispiel die Sichtbarkeit während der Operation erhöhen. Eine lokale Infiltrationsanästhesie (LIA) kann durchgeführt werden. Hierzu werden Schmerzmittel während der Operation in das Gewebe um das Knie herum gespritzt. Zudem ist es möglich, dass während der Operation Drainagen gelegt werden. Hierbei handelt es sich um kleine Kunststoffschläuche, die Blut oder andere Körperflüssigkeiten vom Knie in kleine tragbare Behälter ableiten. Drainagen können nach der Operation noch mehrere Tage im Körper verbleiben und werden dann wieder entfernt. Besprechen Sie mit Ihrem Behandlungsteam, welche dieser Maßnahmen auf Sie zutreffen können.

Ihre Fragen/Notizen

Welche Komplikationen und Risiken können auftreten? [3]

Der Verlauf einer Komplikation kann nicht konkret vorhergesagt werden. Komplikationen können verschiedene Schweregrade haben. Folgen können vorübergehend oder bleibend sein und zu keinen bis stärksten Einschränkungen, einschließlich Tod, führen. Die Folgen von Komplikationen können weitere Behandlungen und ein verlängerter oder erneuter Krankenhausaufenthalt sein. Vor- und Begleiterkrankungen sowie individuelle Besonderheiten können die Häufigkeit von Komplikationen wesentlich beeinflussen.

Wie können die Ergebnisse gelesen werden?

Für die Komplikationen wurden mehrere Studienergebnisse zusammengefasst. Auf Seite 8 finden Sie eine Anleitung wie die Abbildung zum Vergleich von zwei Gruppen gelesen werden kann. Die folgende Abbildung hilft Ihnen, die Häufigkeit der Komplikationen zu verstehen.

Auf Seite 22 finden Sie einen Link zu allen Quellen.

Welche Komplikationen und Risiken können auftreten? [3]

Komplikation | **Häufigkeit**

Blutergüsse

können durch Nachblutungen im Bindegewebe um das Kniegelenk herum entstehen und zu Bewegungseinschränkungen, Schmerzen und Missempfindungen führen. Punktionen oder erneute operative Eingriffe können notwendig werden.

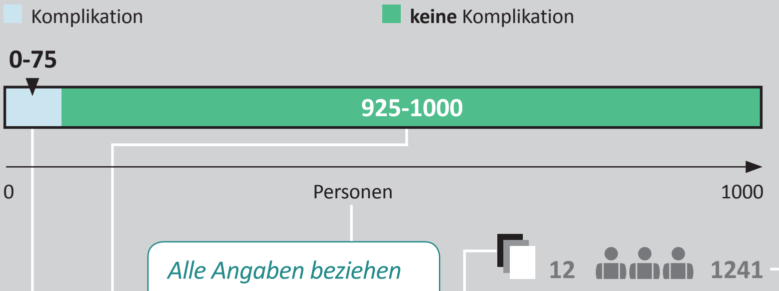

Alle Angaben beziehen sich auf 1000 Personen.

Anzahl der Studien, die in die Darstellung der Ergebnisse eingeflossen sind.

Anzahl der Teilnehmenden, die in den Studien insgesamt beobachtet wurden.

Minimaler und maximaler Anteil an Personen, die nach der OP einen Bluterguss haben.

„Der Anteil an Personen mit einem Bluterguss liegt zwischen 0 und 75 von 1000 Personen (0% - 7,5%).“

*Minimaler und maximaler Anteil an Personen, die nach der OP **keinen** Bluterguss haben.*

*„Der Anteil an Personen mit **keinem** Bluterguss liegt zwischen 925 und 1000 von 1000 Personen (92,5% - 100%).“*

Zahlen sind Schätzungen von Wahrscheinlichkeiten und lassen keine konkreten Vorhersagen für eine Person zu. Fragen Sie Ihr Behandlungsteam, wenn Sie sich unsicher sind, ob sich die Ergebnisse auf Ihre Situation übertragen lassen.

Komplikationen bei verschiedenen Prothesentypen, Verankerungsmethoden oder Operationsverfahren [3, 7]

In randomisiert-kontrollierten Studien wurde nur zum Vergleich einer unbeweglichen mit einer beweglichen Kunststoffgleitfläche bei einer Total-Endoprothese ein Unterschied gefunden. Hier kann eine Einschätzung gegeben werden, wie häufig die Komplikation im Vergleich auftritt.

Komplikation	Total-Endoprothese mit unbeweglicher und beweglicher Kunststoffgleitfläche im Vergleich

Gelenkinfektionen

können schwerwiegende Folgen haben und sind mit erheblichen Bewegungseinschränkungen verbunden. Es können langwierige Behandlungen und ein Austausch der Prothese nötig werden.

weniger Gelenkinfektionen mit unbeweglicher Kunststoffgleitfläche

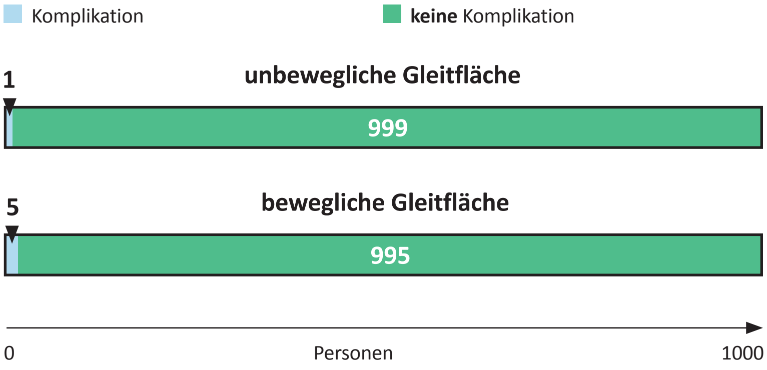

8 1378

Für andere Vergleiche zwischen verschiedenen Prothesentypen, Verankerungsmethoden oder Operationsverfahren konnten in den Studien keine Unterschiede festgestellt werden. Anhand der folgenden Abbildungen erhalten Sie einen Eindruck, wie häufig eine Komplikation unabhängig von den verschiedenen Verfahren auftreten kann.

Komplikation	Häufigkeit

Bluttransfusionen

können infolge von Blutungen nötig werden. Über das Risiko einer Infektion bei Fremdblutübertragung werden Sie bei Bedarf gesondert aufgeklärt.

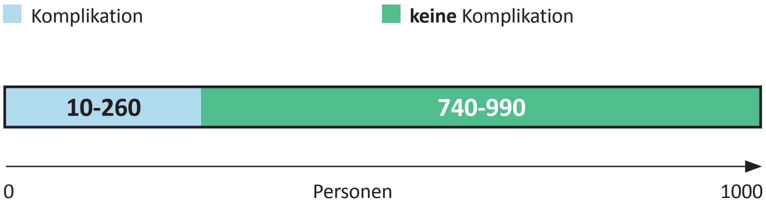

In einer Studie mit insgesamt 100 Teilnehmenden wurde ein höherer Anteil an Bluttransfusionen beobachtet.

13 1667

Ein **Verrutschen der Kniescheibe** kann mit starken Schmerzen verbunden sein und auch zur Schwächung des Streckapparates beitragen. Dies kann eine weitere Operation erfordern.

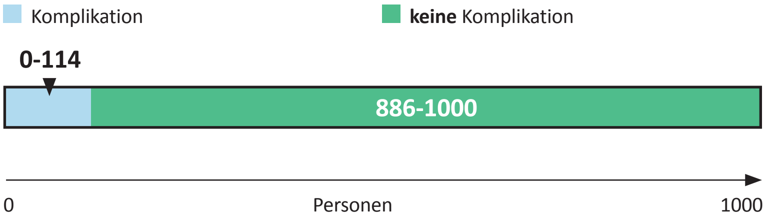

11 850

Komplikation	Häufigkeit

Ein **eingeschränkter Bewegungsumfang (Steifigkeit)** kann sowohl die Beugung als auch das Streckung des Knies betreffen und kann verschiedene Ursachen haben. Neben konservativen Behandlungsmöglichkeiten kann auch eine operative Behandlung notwendig werden.

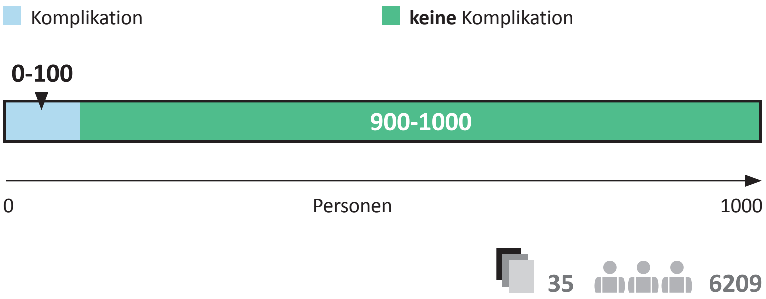

Wundkomplikationen

können in Form von Wundinfektionen, verzögerter Wundheilung, Wundsekretion, Nahtinsuffizienzen, Blasenbildung oder Gewebeschäden auftreten.

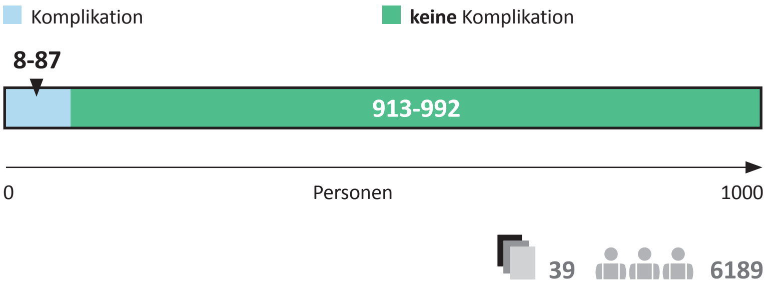

Blutergüsse

können durch Nachblutungen im Bindegewebe um das Kniegelenk herum entstehen und zu Bewegungseinschränkungen, Schmerzen und Missempfindungen führen. Punktionen oder erneute operative Eingriffe können notwendig werden.

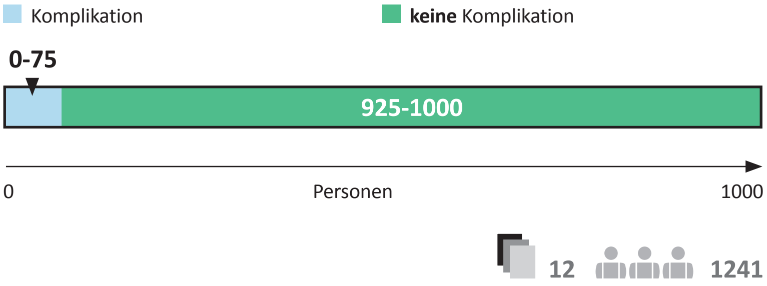

Knochenbrüche

können während oder nach der Operation auftreten und können den Oberschenkelknochen, das Schienbein oder die Kniescheibe betreffen.

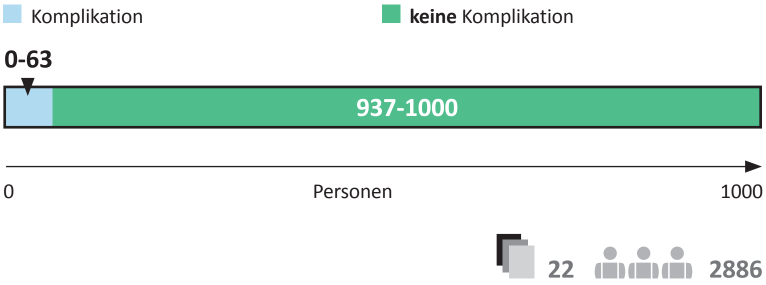

Thrombose / Embolie

Infolge der Operation kann ein Blutgerinnsel den Blutstrom in den Blutgefäßen behindern und stoppen. Werden Blutgefäße verschlossen, kann das lebensgefährliche Folgen haben (z.B. Beinvenenthrombose, Lungenembolie, Herzinfarkt, Schlaganfall).

Durch das Einsetzen der Prothese können Fettpartikel in den Blutstrom gelangen und ähnliche Folgen hervorrufen.

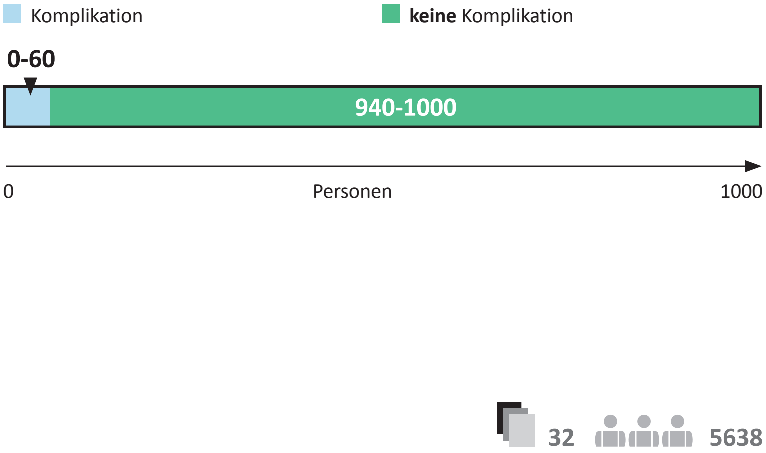

Komplikation	Häufigkeit

Gelenkinfektionen
können schwerwiegende Folgen haben und mit erheblichen Bewegungseinschränkungen und Schmerzen verbunden sein. Es können langwierige Behandlungen und Folgeoperationen mit einem Austausch der Prothese nötig werden. Die Einnahme von Antibiotika gehört ebenfalls zur Therapie.

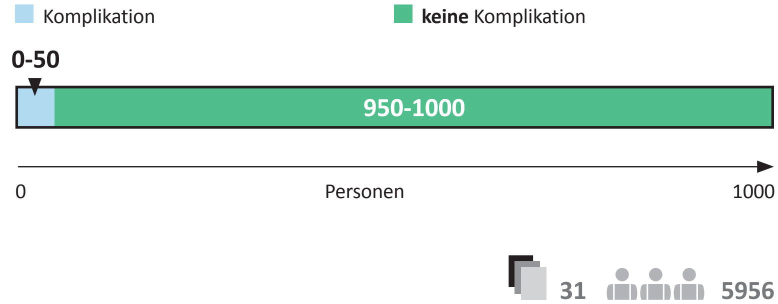

Beeinträchtigungen des Nervensystems
können durch verschiedene Ursachen in Form von Missempfindungen, Bewegungsstörungen oder Lähmungen auftreten und vorübergehend oder bleibend sein.

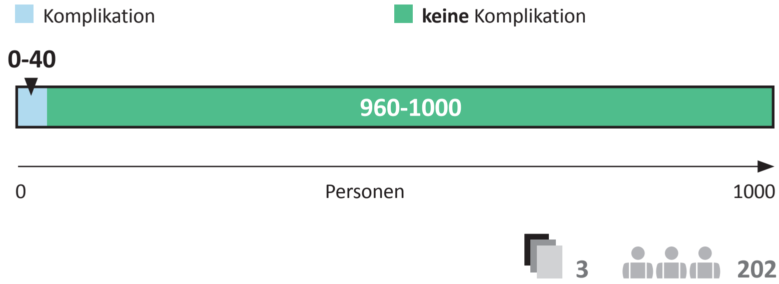

Eine **Verletzung des Streckapparates** des Knies führt zu Bewegungseinschränkungen und Schwäche des Unterschenkels beim Strecken. Dies kann zur Gangunsicherheit führen und eine chirurgische Versorgung erfordern.

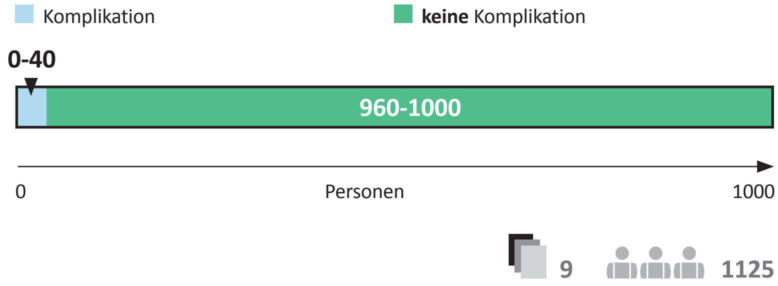

Eine **Lockerung der Prothese** hat verschiedene Ursachen und führt zum Austausch der lockeren Komponente.

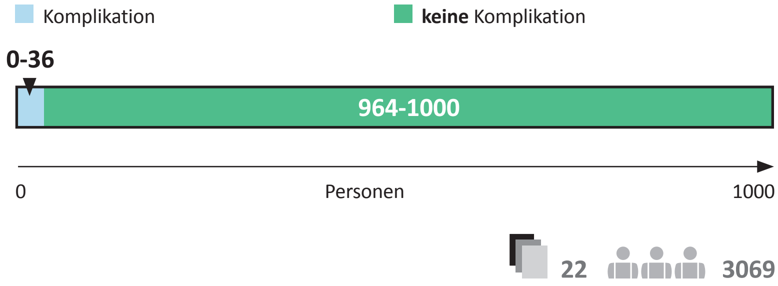

Schwellungen
können als normale Begleiterscheinung einer Operation entstehen. Sie können aber auch zu Bewegungseinschränkungen, Schmerzen und Missempfindungen führen.

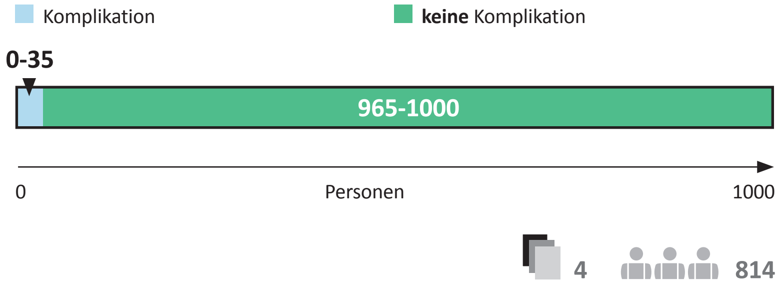

Abweichungen in der Ausrichtung
des künstlichen Gelenks können entstehen, wenn die Positionierung der Prothese nicht optimal gelungen ist und können zu Schmerzen, Bewegungseinschränkungen und langfristig zur früheren Lockerung der Prothese führen.

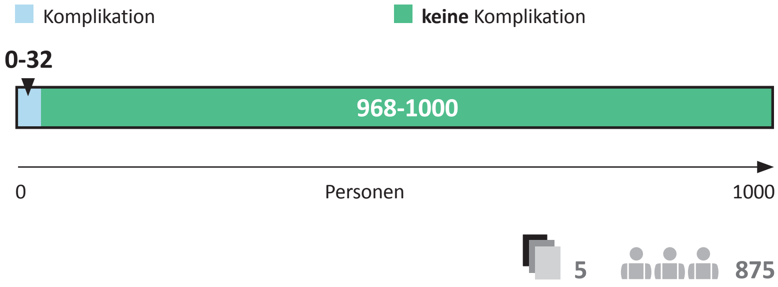

Komplikation	Häufigkeit

Verletzungen des Innenbandes

können während der Operation entstehen und zu Änderungen der Prothesenart führen und nach der Operation Grund für eine erneute Operation sein.

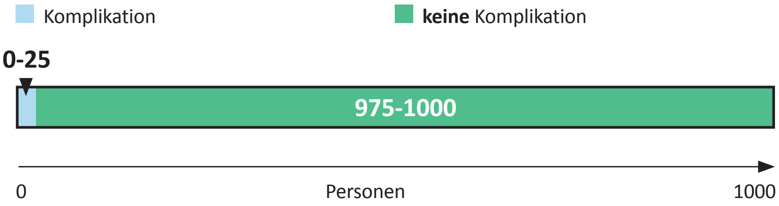

4 395

Instabilität

des Kniegelenks kann durch eine unausgeglichene Spannung des Bandapparates entstehen und äußert sich beispielsweise durch Bewegungseinschränkungen oder Instabilitätsgefühle. Eine Korrekturoperation kann nötig werden

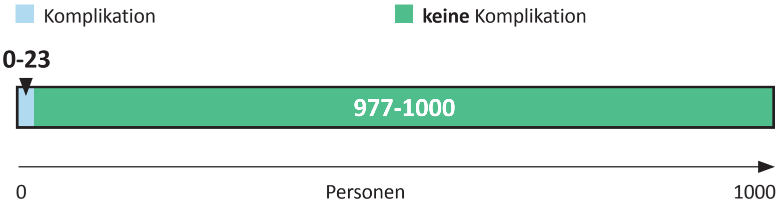

11 2071

Die **Abnutzung der Kunststoffgleitfläche** zwischen den Prothesenkomponenten kann einen operativen Wechsel der Gleitfläche erfordern.

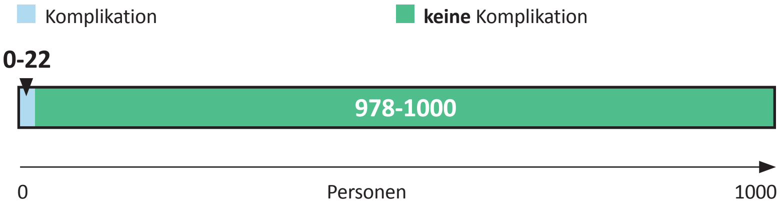

2 194

Mechanische Kniegeräusche

können in verschiedenen Formen während der Bewegung auftreten.

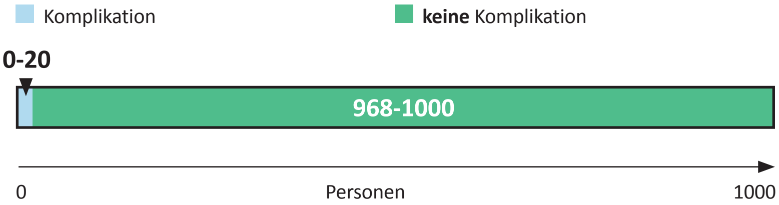

In einer Studie mit insgesamt 60 Teilnehmenden wurde ein höherer Anteil an Kniegeräuschen beobachtet. Dies könnte möglicherweise mit dem verwendeten teilgekoppelten Prothesentyp zusammenhängen.

4 857

Das **Ablösen der Kunststoffgleitfläche** von der unteren Metallkomponente kann bei ungekoppelten Prothesen auftreten und zu Bewegungseinschränkungen führen. Eine erneute Operation mit Austausch der Gleitfläche kann notwendig werden.

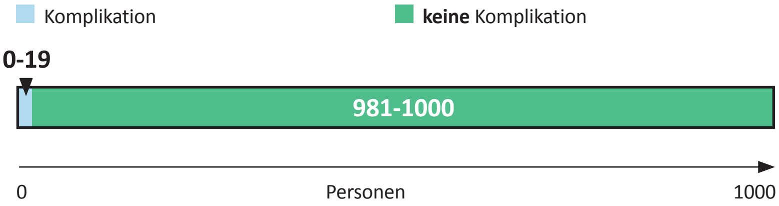

Die angegebenen Zahlen wurden nur aus 3 der 9 Studien berechnet. In den 6 übrigen Studien ist die Komplikation nicht aufgetreten. Ob die angegebene Spanne realistisch ist, ist daher unklar.

9 1296

Komplikation	Häufigkeit
In wenigen Studien wurden **Verrenkungen ohne nähere Bezeichnung** beobachtet. Es ist unklar, ob ein Verrutschen der Kniescheibe oder ein Ausrenken des Kniegelenks beobachtet wurden.	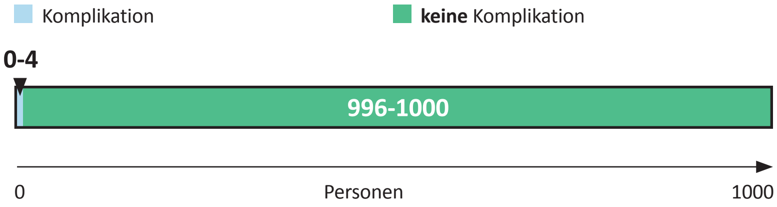 2 2222
Ein **Ausrenken des Kniegelenks** ist mit starken Schmerzen verbunden und birgt die Gefahr einer Gefäß- und Nervenverletzung. Eine weitere Operation mit Wechsel der Prothese kann nötig werden.	Diese Komplikation wurde in 3 Studien betrachtet, ist jedoch nur in einer Studie überhaupt aufgetreten. Es kann daher über die Häufigkeit keine Angabe gemacht werden.<br>3 258
**Gefäßverletzungen**<br>können während oder nach der Operation auftreten und Blutungen verursachen, die chirurgisch versorgt werden müssen und weitere Folgen mit sich bringen können. Es können Bluttransfusionen notwendig werden.	Es ist bekannt, dass Gefäßverletzungen in der Vergangenheit beobachtet wurden. In den zugrundeliegenden Studien wurden keine Gefäßverletzungen berichtet. Eine Aussage zur Häufigkeit ist daher nicht möglich.
Eine **Schwächung oder Auflösung der Knochenstruktur (Osteolyse)** kann zu Lockerungen der Prothese führen und eine Wiederherstellungsoperation nötig machen.	Diese Komplikation wurde in 2 Studien betrachtet, ist jedoch nur in einer Studie überhaupt aufgetreten. Es kann daher über die Häufigkeit keine Angaben gemacht werden.<br>2 383

Wie hoch ist das Risiko, dass Prothesenteile ausgetauscht werden müssen? [3-10]

Vielleicht fragen Sie sich, wie lange die Prothese im Körper verbleiben kann - wie lange diese haltbar ist. Durch verschiedene Ursachen kann es nötig werden, ein oder mehrere Prothesenteile auszutauschen. Mögliche Ursachen sind zum Beispiel: Gelenkinfektionen, Instabilitäten oder die Lockerung der Prothese.

In randomisiert-kontrollierten Studien, die den Nachweis erbringen können, ob eine Behandlung besser als eine andere ist (siehe Seite 3), zeigten sich keine Unterschiede zwischen den verschiedenen Prothesentypen, Operationsverfahren und Verankerungsmethoden.

Unabhängig von Prothesentyp, Operationsverfahren und Verankerungsmethode liegt der Anteil der Personen, bei denen Prothesenteile ausgetauscht werden müssen, zwischen 0 und 97 von 1000. Die Personen wurden zwischen 3 Monaten und 15 Jahren beobachtet.

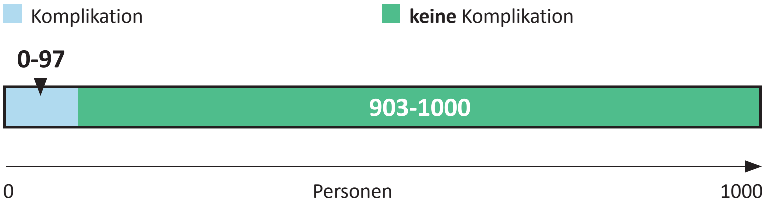

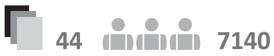

Zusätzlich haben wir Daten aus sogenannten Registerstudien angeschaut.

In manchen Ländern werden große Datenbanken zu den eingebauten Knie-Endoprothesen angelegt. Dabei wird neben den Details zur Operation auch erfasst, ob Prothesenteile ausgetauscht werden mussten.

Die Ergebnisse aus Registerstudien beruhen auf den Daten von sehr vielen Personen. Sie erfüllen aber nicht die methodischen Standards von randomisiert-kontrollierten Studien. Es kann daher nicht sicher gesagt werden, ob ein Unterschied in den Ergebnissen tatsächlich auf eine bestimmte Behandlung oder unterschiedliche Ausgangsvoraussetzungen zurückzuführen ist.

Für den Vergleich zwischen Standard- und minimalinvasivem Zugang sowie Standard- und robotergestützter Navigation sind keine Registerdaten verfügbar.

Bei den Vergleichen zu einer Total-Endoprothese mit oder ohne zementierter Befestigung, Standard- oder computergestützten Navigation und Standard- oder patientenindividuellen Instrumenten konnten auch in den Registerstudien keine Unterschiede gefunden werden.

Total-Endoprothese verglichen mit Teil-Endoprothese: In Daten von 176.569 Operationen konnten Hinweise gefunden werden, dass mit einer Total-Endoprothese ein geringerer Anteil an Prothesen ausgetauscht werden musste, als mit einer Teil-Endoprothese. Die Personen wurden zwischen 5 und 8 Jahren beobachtet.

Ungekoppelte Total-Endoprothese verglichen mit einer stabilisierter Total-Endorothese: In Daten von 639.139 Operationen konnten Hinweise gefunden werden, dass bei ungekoppelten Total-Endoprothesen ein geringerer Anteil an Prothesen ausgetauscht werden musste, als bei stabilisierten Prothesen. Die Personen wurden 2 Jahre beobachtet.

Total-Endoprothese ohne den Ersatz der Kniescheibenrückseite verglichen mit dem Ersatz der Kniescheibenrückseite: In Daten von 898.032 Operationen konnten Hinweise gefunden werden, dass bei Total-Endoprothesen ohne Ersatz der Kniescheibenrückseite ein größerer Anteil an Prothesen ausgetauscht werden musste, als bei Prothesen mit Ersatz der Kniescheibenrückseite. Die Personen wurden zwischen 2 und 5 Jahren beobachtet.

Total-Endoprothese mit einer unbeweglichen Kunststoffgleitfläche verglichen mit einer beweglichen Kunststoffgleitfläche: In Daten von 958.843 Operationen konnten Hinweise gefunden werden, dass bei Total-Endoprothesen mit unbeweglicher Gleitfläche ein geringerer Anteil an Prothesen ausgetauscht werden musste, als bei Prothesen mit beweglicher Gleitfläche. Die Personen wurden zwischen 2 und 10 Jahren beobachtet.

Welche weiteren Komplikationen und unerwünschte Ereignisse können auftreten? [3]

Es ist bekannt, dass die folgenden Ereignisse und Komplikationen in der Vergangenheit aufgetreten sind. Wie häufig diese aber tatsächlich auftreten, kann aus den vorliegenden Studien nicht ermittelt werden.

Allergische Reaktionen und Unverträglichkeiten

Allergische Reaktionen und Unverträglichkeiten zum Beispiel auf Latex, Medikamente, Desinfektionsmittel können zu einem akuten Kreislaufschock führen. Als Folge kann es zu lebensbedrohlichen Umständen und intensivmedizinischer Betreuung kommen.

Unverträglichkeiten gegenüber den Prothesenkomponenten oder Fixierungsmittel (z.B. Knochenzement) können zur Lockerung sowie Wundinfektionen und Hautreizungen führen. Als Folge kann ein Austausch des künstlichen Gelenks notwendig werden.

Amputationen

Infolge von Komplikationen kann die Amputation des betreffenden Beins notwendig werden.

Beinlängendifferenz

Infolge der Operation kann es zu unterschiedlichen Beinlängen kommen. Bei kleineren Unterschieden können Einlagen und bei größeren Unterschieden Absatzerhöhungen verwendet werden.

Gelenkversteifung

Infolge von Komplikationen kann die vollständige Versteifung des Kniegelenks notwendig werden.

Materialbruch

Prothesen und weitere verwendete Materialien sowie Instrumente können zum Beispiel aufgrund von Materialermüdung oder Materialschäden brechen.

Unerwünschte Ereignisse

Die folgenden Ereignisse traten in der Vergangenheit zusammen mit dem Einsetzen einer Total-Endoprothese auf. Ob diese Ereignisse auf die Total-Endoprothese zurückzuführen sind oder ob diese andere Ursachen hatten, ist unklar.

- Dekubitus (Druckgeschwüre)
- Gelenkverformung
- Gicht (Stoffwechselstörung)
- Lymphödem (Flüssigkeitsansammlung unter der Haut)
- Pseudoaneurysma (Hämatom einer Arterie)
- Rückenschmerzen
- Sehbeeinträchtigung (kurzzeitige) oder Erblindung
- Sturz
- Veränderung der oberflächlichen Hautschichten

Was gibt es vor dem Eingriff zu beachten? [1]

Was sollte ich beachten, wenn ich Medikamente einnehme?

Informieren Sie das Behandlungsteam über alle Medikamente (auch pflanzliche oder rezeptfreie), die Sie derzeit einnehmen - insbesondere blutgerinnungshemmende Medikamente (z.B. Heparin, Marcumar©, ASS [Aspirin©] etc.). Beachten Sie, dass Medikamente nur in Absprache mit dem Behandlungsteam eingenommen oder abgesetzt werden sollten.

Geben Sie bitte wichtige Unterlagen wie zum Beispiel Ausweise (Allergie, Röntgen, Implantat, Marcumar©), Befunde oder Röntgenbilder an das Behandlungsteam weiter.

Sollte ich vor der Operation aufhören zu rauchen?

Wenn Sie vor der Operation das Rauchen einstellen, kann sich dies positiv auf den Heilungsprozess auswirken. Sie können damit das Auftreten bestimmter Komplikationen (z.B. Wundheilungsstörungen) reduzieren.

Vorbereitungen für die Entlassung

i

Informationen zur Vorbereitung für die Entlassung erhalten Sie in der Informationsbroschüre zum Kniegelenksersatz auf der Seite 31.

Was gibt es nach dem Eingriff zu beachten? [1]

Auf welche Warnzeichen muss ich nach der Operation achten?

Falls Sie eines der folgenden Anzeichen bei sich nach der Operation feststellen, informieren Sie bitte unverzüglich Ihr Behandlungsteam: starke Schmerzen, Missempfindungen, Störungen der Bewegungsfähigkeit, Verfärbungen der Haut, Übelkeit, Luftnot oder Fieber.

Mit welchen Einschränkungen meiner Mobilität sollte ich nach der Operation rechnen?

Ihre Mobilität wird in der ersten Zeit nach dem Eingriff eingeschränkt sein. Sie erhalten Informationen vom Behandlungsteam, wie lange mit Einschränkungen zu rechnen sein wird. Außerdem erhalten Sie Hilfsmittel, die Ihnen das Gehen erleichtern. Das können zum Beispiel Gehstützen oder ein Gehwagen sein. In der Zeit nach der Operation kann die Sturzgefahr höher sein. Möglicherweise dürfen Sie das operierte Bein nicht oder nur teilweise belasten. Beachten Sie hier bitte die Informationen des Behandlungsteams. Es kann notwendig sein, körperliche und sportliche Aktivitäten auch über die Akutphase hinaus anzupassen. Informieren Sie sich bei Ihrem Behandlungsteam über individuelle Möglichkeiten.

Erhalte ich nach der Operation bestimmte Medikamente?

In der Regel erhalten Sie nach der Operation blutverdünnende Medikamente, um einer Thrombose vorzubeugen. Es kann dadurch zu vermehrten Blutungen allgemein sowie zu Nachblutungen kommen. Informieren Sie vor weiteren Eingriffen, zum Beispiel beim Zahnarzt, das Behandlungsteam darüber.

Wie erfolgt die Nachbehandlung/Rehabilitation?

Nachdem Sie aus der Klinik entlassen werden, wird Ihnen eine anschließende Nachbehandlung angeboten. Hierzu zählen die Anschlussrehabilitation, Kontrolluntersuchungen und Physiotherapie. Die Anschlussrehabilitation kann sowohl stationär als auch ambulant stattfinden. Ihr Behandlungsteam wird Sie über beide Varianten informieren.

Hinweise für die Zeit nach der Operation

i

Informationen für die Zeit nach der Operation, zum Beispiel zur Arbeits- oder Fahrtüchtigkeit, erhalten Sie in der Informationsbroschüre zum Kniegelenksersatz auf der Seite 32.

Wo finde ich weitere Informationen?

Weitere Informationen zur Kniearthrose und deren Behandlungsmöglichkeiten finden Sie auf der Internetseite des **Instituts für Qualität und Wirtschaftlichkeit im Gesundheitswesen** (IQWiG).

www.gesundheitsinformation.de/kniearthrose-gonarthrose.html

Informationen und Möglichkeiten zur Selbsthilfe finden Sie außerdem auf folgenden Internetseiten:

Deutsche Arthrose Stiftung

www.deutsche-arthrose-stiftung.de

Deutsche Arthrose-Hilfe e.V.

www.arthrose.de

Wer hat den Aufklärungsbogen mit welchen Quellen, wie erstellt?

Auf dieser Webseite sind alle verwendeten Quellen sowie alle beteiligten Personen aufgelistet.
Außerdem finden Sie hier den Methodenreport.

https://evab-pilot.leitlinie-gesundheitsinformation.de

Wichtige Fragen zu Ihrem Gesundheitszustand [1]

Das Risiko ärztlicher Eingriffe kann durch Ihre körperliche Verfassung und Vorschäden beeinflusst werden. Wir bitten Sie daher, folgende Fragen zu beantworten. Anhand dieser Informationen kann das Behandlungsteam Risiken in Ihrem speziellen Fall besser abschätzen und ggf. Maßnahmen ergreifen, um Komplikationen zu vermeiden.

Alter (Jahre):	Größe (cm):	Gewicht (kg):
Geschlecht: ☐ weiblich	☐ männlich	☐ divers
Könnten Sie schwanger sein?	☐ ja	☐ nein

1. Nehmen Sie **Medikamente** ein (z.B. gerinnungshemmende Mittel wie bspw. Marcumar® oder Aspirin ®, Schmerzmittel, insbesondere metforminhaltige Antidiabetika, Herz-/Kreislauf-Medikamente, Hormonpräparate, Schlaf- oder Beruhigungsmittel, blutdrucksenkende Mittel)? ☐ ja ☐ nein
Wenn ja, welche?

2. Besteht eine **Allergie** (z.B. auf Betäubungsmittel, Röntgenkontrastmittel, Latex, Desinfektionsmittel, Jod, Pflaster, Kunststoffe oder Medikamente wie z.B. Antibiotika, Novalgin oder Paracetamol)? ☐ ja ☐ nein
Wenn ja, welche?

3. Besteht eine **Allergie oder Überempfindlichkeit** gegenüber Metallen (z.B. Nickel, Kobalt, Chrom, Molybdän) oder dem Wirkstoff Benzoylperoxid? ☐ ja ☐ nein
Wenn ja, welche?

4. Besteht bei Ihnen eine **erhöhte Blutungsneigung?** Haben Sie z.B. häufig Nasen- oder Zahnfleischbluten, blaue Flecken oder nach einer Operation nachgeblutet? ☐ ja ☐ nein

5. Besteht in Ihrer **Blutsverwandtschaft** eine erhöhte **Blutungsneigung**? ☐ ja ☐ nein

6. Ist bei Ihnen schon einmal eine **Übertragung von Blut oder Blutbestandteilen** (Transfusion) erfolgt ☐ ja ☐ nein

7. Besteht oder bestand bei Ihnen eine **Infektionskrankheit** (z.B. Hepatitis, HIV/AIDS, Hirnhautentzündung, Tuberkulose)? ☐ ja ☐ nein
Wenn ja, welche?

8. Besteht derzeit oder bestand in den letzten 6 Monaten eine **Entzündung** (z.B. an einem Zahn)? ☐ ja ☐ nein
Wenn ja, wo?

9. Besteht bei Ihnen eine **Stoffwechselerkrankung** (z.B. Zuckerkrankheit, Gicht)? ☐ ja ☐ nein
Wenn ja, welche?

10. Besteht oder bestand bei Ihnen eine **Herz-Kreislauf-Erkrankung** (z.B. koronare Herzkrankheit, Bluthochdruck, Rhythmusstörungen, Schlaganfall, Herzinfarkt, Angina pectoris, Herzmuskelentzündung, Klappenfehler)?

☐ ja ☐ nein

Wenn ja, welche?

---

11. Besteht oder bestand bei Ihnen eine **Atemwegs- oder Lungenerkrankung** (z.B. chronische Bronchitis, Lungenentzündung, Asthma bronchiale, Lungenblähung, angeborene Fehlbildung)?

☐ ja ☐ nein

Wenn ja, welche?

---

12. Besteht oder bestand bei Ihnen eine **Erkrankung der Nieren oder Harnorgane** (z.B. Nierenfunktionsstörung, Nierensteine, chronischer Harnwegsinfekt, Nierenentzündung, angeborene Fehlbildung wie bspw. eine Doppelniere, Blasenentleerungsstörung)?

☐ ja ☐ nein

Wenn ja, welche?

---

13. Besteht oder bestand bei Ihnen eine **Muskel- oder Skeletterkrankung** (z.B. Muskelschwäche, Gelenkerkrankung, Osteoporose, Osteomalazie)?

☐ ja ☐ nein

Wenn ja, welche?

---

14. Besteht oder bestand bei Ihnen eine **Erkrankung des Nervensystems** (z.B. Gehstörungen, Lähmungen, Krampfleiden (Epilepsie), Parkinson, Gefühlsstörungen, Polyneuropathie, Schmerzen)?

☐ ja ☐ nein

Wenn ja, welche?

---

15. Bestehen **weitere Erkrankungen?**

☐ ja ☐ nein

Wenn ja, welche?

---

16. Kam es bei Ihnen schon einmal zu einem **Gefäßverschluss** durch Blutgerinnsel (Thrombose / Embolie)? ☐ ja ☐ nein

17. Neigen Sie zu **Wundheilungsstörungen?** ☐ ja ☐ nein

18. Kam es schon einmal zu einer **Narbenwucherung** (z.B. Keloid)? ☐ ja ☐ nein

19. **Rauchen Sie?** ☐ ja ☐ nein

Anmerkungen zum Aufklärungsgespräch *(durch das Behandlungsteam auszufüllen)*

(z.B. individuelle Risiken und mögliche Komplikationen, Fragen der Patientin/des Patienten, mögliche Folge- oder Nebeneingriffe, möglicher Verlauf bei Verschiebung oder Ablehnung des Eingriffs, Gründe der Patientin/des Patienten für eine Verschiebung/Ablehnung, Einschränkungen im Umfang der Einwilligung, gesetzliche Vertretung der Patientin/des Patienten)

Der Gelenkersatz erfolgt am:

- ☐ rechten Knie
- ☐ linken Knie

Prothesenarten:

- ☐ ungekoppelte Prothese
- ☐ stabilisierte Prothese
- ☐ gekoppelte Prothese

Verankerungsmethode:

- ☐ mit Zement
- ☐ ohne Zement
- ☐ teilweise zementiert

Vorgesehener Eingriff:

- ☐ Teil-Endoprothese
- ☐ Total-Endoprothese

Operationsverfahren:

- ☐ mit computergestützter Navigation
- ☐ ohne computergestützter Navigation

Folgende Optionen sind außerdem für Sie angedacht. Während der Operation können sich hier jedoch noch Änderungen ergeben, da sich eine endgültige Einschätzung erst währenddessen treffen lässt:

Prothesenarten:

- ☐ Ersatz der Kniescheibenrückfläche
- ☐ kein Ersatz der Kniescheibenrückfläche
- ☐ bewegliche Kunststoffgleitfläche
- ☐ unbewegliche Kunststoffgleitfläche

Operationsverfahren:

- ☐ mit patientenindividuellen Instrumenten
- ☐ ohne patientenindividuellen Instrumenten
- ☐ mit minimalinvasivem Zugang
- ☐ ohne minimalinvasivem Zugang
- ☐ mit robotergestützter Navigation
- ☐ ohne robotergestützter Navigation

Ihre Fragen/Notizen

Einwilligung

Mit der Einwilligung bestätigen Sie, dass Sie die für Ihre Entscheidung notwendigen Informationen zur geplanten Operation, einschließlich der Art und Bedeutung, zu erwartenden Nutzen, Behandlungsalternativen, Risiken, mögliche Komplikationen, mögliche Neben- oder Folgeeingriffe und mögliche Abweichungen vom geplanten Eingriff in einer für Sie verständlichen Art und Weise erhalten haben.

Sie bestätigen, dass alle für Sie wichtigen Fragen verständlich geklärt werden konnten und keine weiteren Fragen bestehen.

Sie bestätigen, dass Sie mit medizinisch erforderlichen Änderungen und Erweiterungen der Operation sowie Neben- oder Folgeeingriffen, die direkt während der geplanten Operation stattfinden müssen, ebenso einverstanden sind.

☐ Ich willige in die vorgeschlagene Operation ein.

Ort, Datum , Uhrzeit

Patientin/Patient oder gesetzliche(r) Vertreter(in)

Ärztin/Arzt

☐ Ich habe eine Kopie des Aufklärungsbogens erhalten

☐ Ich möchte keine Kopie des Aufklärungsbogens erhalten.

Ort, Datum , Uhrzeit

Patientin/Patient oder gesetzliche(r) Vertreter(in)

MARTIN-LUTHER-UNIVERSITÄT
HALLE-WITTENBERG

universität Witten/Herdecke

Aktualität: 2020
Nächste geplante Aktualisierung: 2024

Impressum

Erstellung durch das EvAb-Pilot-Projektteam

Kontakt:

Prof. Dr. phil. Anke Steckelberg

Institut für Gesundheits- und Pflegewissenschaft

Martin-Luther-Universität Halle-Wittenberg

Anke.Steckelberg@medizin.uni-halle.de

Das Projekt, in dem dieses Informationsmaterial entstanden ist, wurde mit Mitteln des Innovationsausschusses beim Gemeinsamen Bundesausschuss unter dem Förderkennzeichen 01VSF19025 gefördert.

Illustration und Layout durch Martin Siegmund (Siegmund und Fischer Grafik)

Künstliches Kniegelenk

Informationen zum Nutzen verschiedener Operationsmöglichkeiten und zur Vorbereitung auf die Operation

MARTIN-LUTHER-UNIVERSITÄT
HALLE-WITTENBERG

universität Witten/Herdecke

MARTIN-LUTHER-UNIVERSITÄT
HALLE-WITTENBERG

universität Witten/Herdecke

Aktualität: **2020**
Nächste geplante Aktualisierung: **2024**

Impressum

Erstellung durch das EvAb-Pilot-Projektteam

Kontakt:
Prof. Dr. phil. Anke Steckelberg
Institut für Gesundheits- und Pflegewissenschaft
Martin-Luther-Universität Halle-Wittenberg
Anke.Steckelberg@medizin.uni-halle.de

Das Projekt, in dem dieses Informationsmaterial entstanden ist, wurde mit Mitteln des Innovationsausschusses beim Gemeinsamen Bundesausschuss unter dem Förderkennzeichen 01VSF19025 gefördert.

Illustration und Layout durch Martin Siegmund (Siegmund und Fischer Grafik)

Künstliches Kniegelenk

Informationen zum Nutzen verschiedener Operationsmöglichkeiten und zur Vorbereitung auf die Operation

Sehr geehrte Patientin, sehr geehrter Patient,

bei Ihnen wurde eine Kniearthrose festgestellt. Sie erwägen, den von der Arthrose betroffenen Teil des Knies durch eine Total-Endoprothese ersetzen zu lassen. In dieser Broschüre stellen wir Ihnen die verschiedenen Operationsmöglichkeiten und deren Vor- und Nachteile vor. Damit möchten wir Sie bei dem Gespräch mit Ihrer Ärztin oder Ihrem Arzt unterstützen und eine informierte Entscheidung ermöglichen. Weiterhin erhalten Sie Informationen, wie Sie sich auf die Zeit nach der Operation vorbereiten können.

An wen richtet sich die Information?

Die folgenden Informationen richten sich an Patientinnen und Patienten mit Kniearthrose (Gonarthrose). Wenn Sie unter schweren Fehlstellungen oder Verletzungen nahe dem Kniegelenk leiden, oder bei Ihnen eine operative Korrektur von Fehlstellungen erfolgte, erhalten Sie vom Behandlungsteam Informationen zu Ihrer individuellen Situation. Diese Informationen gelten nicht, wenn bei Ihnen ein künstliches Kniegelenk ausgetauscht werden soll oder wenn Sie Rheuma (rheumatoide Arthritis) haben.

Inhalt

Welche Möglichkeiten gibt es, die Kniearthrose mit einem künstlichen Gelenk zu behandeln? 4
Einbau einer Total-Endoprothese: Wie unterscheiden sich die verschiedenen Möglichkeiten? 5-9
Was sollte ich zu den Informationen in dieser Broschüre wissen? 10
Welche Vor- und Nachteile haben die verschiedenen Operationsmöglichkeiten? 11-30
Was kann ich für die Entlassung vorbereiten? 31
Hinweise für die Zeit nach der Operation 32
Wo finde ich weitere Informationen? 33
Wer hat die Informationsbroschüre mit welchen Quellen, wie erstellt? 33

Welche Möglichkeiten gibt es, die Kniearthrose mit einem künstlichen Gelenk zu behandeln?

Auf den Seiten 11-30 finden Sie Informationen zu den Vor- und Nachteilen der verschiedenen Möglichkeiten.

i

Teil-Endoprothese [1]

Sind die Bänder des Knies intakt und ist nur ein Teil des Knies von Arthrose betroffen, kann eine Teil-Endoprothese eingesetzt werden. Sie wird auch Schlittenprothese genannt. Bei dieser Form der Endoprothese werden ausschließlich die betroffenen Gelenkflächen des Oberschenkelknochens sowie des Schienbeins entfernt und durch Metallkomponenten ersetzt. Der gesunde Bereich des Knies bleibt erhalten. Der Eingriff kann unter verschiedenen Anästhesieverfahren erfolgen. Die Aufklärung über die Anästhesie erfolgt gesondert.

Die Qualität der Arbeit der Ärztin oder des Arztes kann beim Einbau einer Teil-Endoprothese von besonderer Bedeutung sein. Erfragen Sie daher die Erfahrung ihrer Ärztin oder Ihres Arztes mit Teilprothesen.

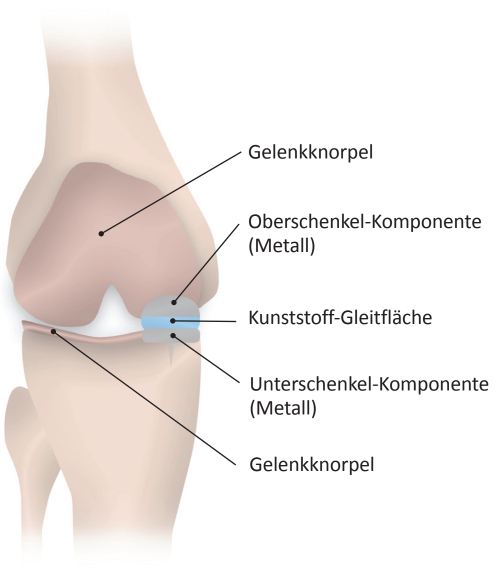

Abbildung 1:
Teil-Endoprothese (frontal)

Total-Endoprothese [1]

Bei einer Total-Endoprothese werden die gesamten Gelenkflächen des Oberschenkelknochens und des Schienbeins ersetzt. Eine Total-Endoprothese besteht aus einer oberen und einer unteren Komponente aus Metall. Zwischen diesen Metallkomponenten wird bei den meisten Prothesen eine Gleitfläche aus Kunststoff gesetzt. Für die Total-Endoprothese stehen Prothesentypen mit verschiedenen Eigenschaften zur Verfügung. Im nächsten Abschnitt erhalten Sie Informationen dazu. Der Eingriff kann unter verschiedenen Anästhesieverfahren erfolgen. Die Aufklärung über die Anästhesie erfolgt gesondert.

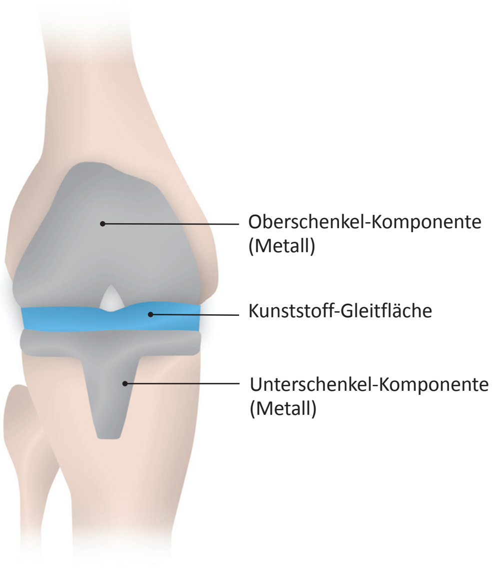

Abbildung 2:
Total-Endoprothese (frontal)

Einbau einer Total-Endoprothese:
Wie unterscheiden sich die verschiedenen Möglichkeiten?

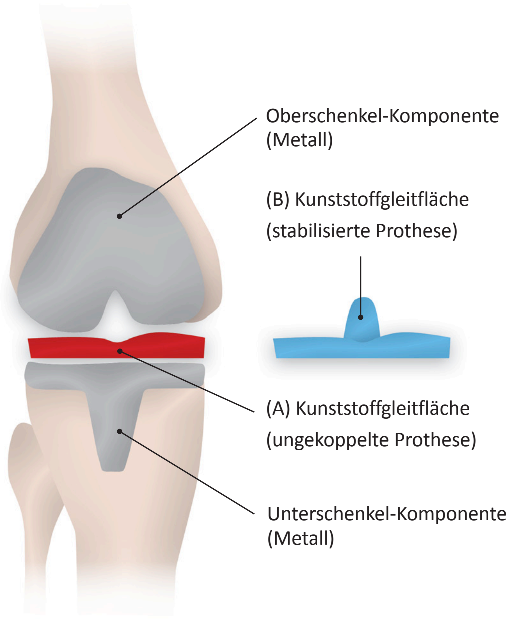

Abbildung 3:
Total-Endoprothese mit Kopplungsgraden (frontal)

Welche verschiedenen Typen von Total-Endoprothesen gibt es?

Total-Endoprothese mit verschiedenen Kopplungsgraden [1]

Bei der Wahl der Prothesenart stehen verschiedene Kopplungsgrade zur Auswahl. Der Begriff „Kopplungsgrad“ steht dafür, in welchem Umfang die obere und untere Metallkomponente miteinander verbunden sind. Wie hoch der Grad der Kopplung sein muss, hängt von mehreren Faktoren ab. Beispielsweise davon wie stabil die Bänder sind. Sind alle Bänder noch ausreichend intakt, kann eine ungekoppelte Prothese eingebaut werden. In diesen Fall wird das künstliche Kniegelenk durch den natürlichen Bandapparat gestützt. Bei unzureichender Stabilität der Bänder, kann eine stabilisierte Prothese in Frage kommen. Hierbei wird das hintere Kreuzband ersetzt und eine Kunststoffgleitfläche mit einem Zapfen verwendet, der das künstliche Kniegelenk stabilisiert. Bei schweren Bandschäden können Prothesen mit einem höheren Kopplungsgrad notwendig werden. Bei einer gekoppelten Prothese sind beide Metallkomponenten mit einer Art Scharnier verbunden.

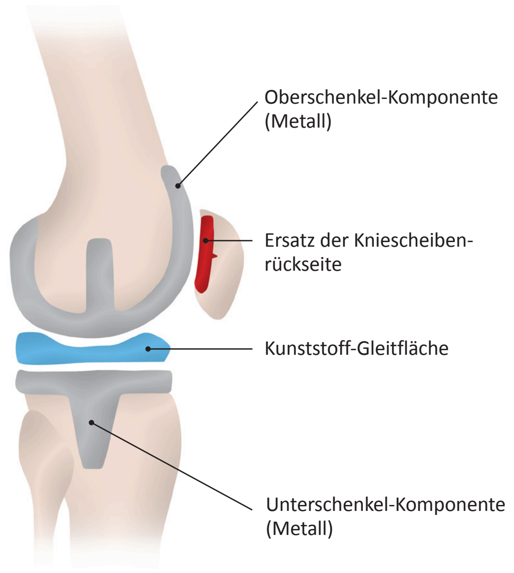

Abbildung 4:
Total-Endoprothese mit Ersatz der Kniescheibenrückseite (seitlich)

Total-Endoprothese mit oder ohne Ersatz der Kniescheibenrückseite [1]

Die Rückseite der Kniescheibe ist ebenfalls von Knorpel überzogen. Auch dieser kann von der Arthrose betroffen sein. Wenn dies der Fall ist, kann er ebenfalls durch eine künstliche Gelenkfläche ersetzt werden.

Total-Endoprothese mit unbeweglicher oder beweglicher Kunststoffgleitfläche [1]

Bei der Art der Kunststoffgleitfläche zwischen den Metallkomponenten können zwei Methoden unterschieden werden. Bei der einen Methode wird die Gleitfläche unbeweglich an der unteren Metallkomponente fixiert. Bei der anderen wird die Gleitfläche beweglich an der unteren Metallkomponente befestigt. Damit soll die Roll-Gleitbewegung eines menschlichen Kniegelenks nachempfunden werden.

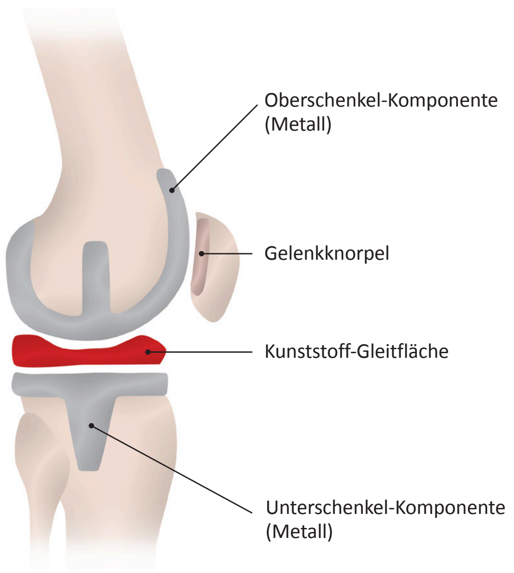

Abbildung 5: Total-Endoprothese mit oder ohne bewegliche Kunststoffgleitfläche (seitlich)

Welche verschiedenen Verankerungsmethoden gibt es?

Total-Endoprothese mit oder ohne zementierte Befestigung [1]

Es gibt verschiedene Methoden, wie die Prothese am Knochen befestigt werden kann. Zementfrei eingebaute Prothesenteile werden auf den Knochen gepresst und verwachsen durch ihre raue Oberfläche dauerhaft mit dem Knochen. Bei einer zementierten Prothese kommt spezieller Knochenzement zum Einsatz. Es können beide Teile oder nur ein Teil der Prothese so befestigt werden.

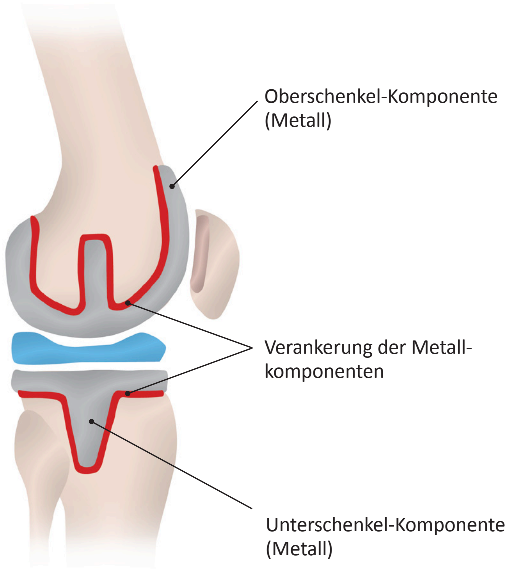

Abbildung 6: Total-Endoprothese mit Verankerungspunkten (seitlich)

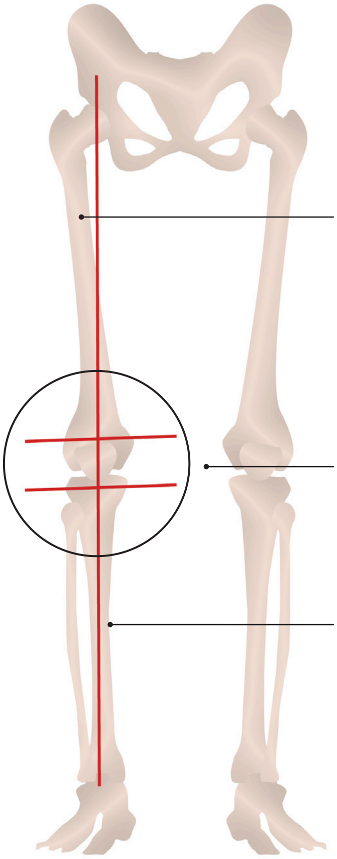

Abbildung 7:
Beinachse (frontal)

Welche Operationsverfahren gibt es?

Total-Endoprothese mit oder ohne computergestützter Navigation [1]

Durch eine computergestützte Navigation während der Operation soll das Ausrichten der Prothese entlang der Beinachse unterstützt werden. Anhand von Fixpunkten berechnet ein Computerprogramm die optimale Position der Prothesenteile. Die Operation selbst führt die Ärztin oder der Arzt durch.

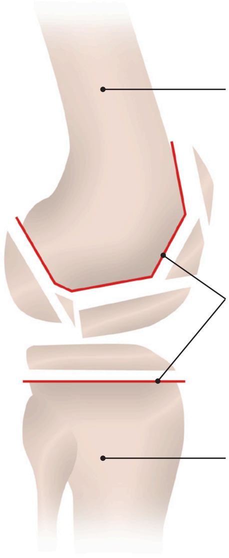

Abbildung 8:
Total-Endoprothese mit Zuschnitt (seitlich)

Total-Endoprothese mit oder ohne patientenindividuellen Instrumenten [1]

Um die Prothesenteile optimal an den Knochen anzupassen, werden die Knochenenden durch Zuschnitte vorbereitet. Dazu werden „Schnittblöcke" an den Gelenkflächen befestigt. Diese dienen als Schablone für die Zuschnitte. Die Schnittblöcke können entweder eine Standardform haben oder auf Patientinnen und Patienten individuell angepasst werden. Dies geschieht anhand von CT- oder MRT-Aufnahmen des Kniegelenkes.

Total-Endoprothese mit oder ohne minimalinvasiven Zugang [1]

Um das Kniegelenk unter den Hautschichten freizulegen, gibt es verschiedene Methoden. Neben einem normalen Schnitt, kann auch ein minimalinvasiver Schnitt in Frage kommen. Der Hautschnitt und die zurückbleibende Narbe sind hierbei kleiner. Allerdings ist die Übersicht innerhalb des Gelenks mit kleinerem Schnitt geringer.

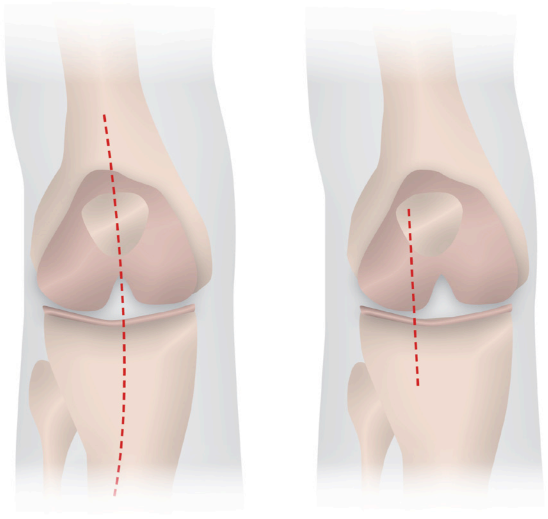

Abbildung 9:
Standard- und minimalinvasiver Zugang (frontal)

Total-Endoprothese mit oder ohne robotergestützte Navigation [1]

Die robotergestützte Navigation wurde entwickelt, um Prothesenteile genauer an der Beinachse ausrichten zu können. Es werden verschiedene Robotersysteme zum Einbau künstlicher Kniegelenke eingesetzt. Es gibt Roboter-Arme, die unter Kontrolle einer Ärztin oder eines Arztes eigenständig operieren oder vom Operierenden gesteuert werden. Außerdem gibt es Roboter, die nur Informationen liefern während der Operation. Ob robotergestützte Navigation in Ihrer Klinik eingesetzt wird, erfahren Sie von Ihrer behandelnden Ärztin oder ihrem behandelnden Arzt.

Disziplinübergreifendes Therapie- / Behandlungskonzept

Total-Endoprothese mit oder ohne „Fast Track" [1]

Das „Fast-Track"-Verfahren ist ein Behandlungs- und Therapiekonzept, bei dem verschiedene medizinische Berufe eng zusammenarbeiten. Ziel ist es, die Ergebnisse einer Operation für Patientinnen und Patienten zu verbessern.. Was genau in dem Konzept eingesetzt wird, kann sich von Klinik zu Klinik unterscheiden. Bisher gibt es dazu in Deutschland keinen Standard. Aus diesem Grund kann der erwartbare Nutzen solcher Konzepte nicht eingeschätzt werden.

Ihre Fragen/Notizen

Was sollte ich zu den Informationen in dieser Broschüre wissen?

Diese Broschüre wurde mit den Methoden der evidenzbasierten Medizin entwickelt. Hierbei werden die derzeit besten verfügbaren Studien als Informationsquelle genutzt.

Woher weiß man ob eine Behandlung besser ist als eine andere?

Um herauszufinden, ob eine Behandlung besser ist als eine andere, werden sogenannte randomisierte-kontrollierte Studien durchgeführt. Wie eine solche Studie funktioniert und warum diese Art von Studien zum Nachweis der Wirksamkeit so wichtig ist, möchten wir Ihnen am folgenden Beispiel zeigen.

Kann der Einsatz einer Teil-Endoprothese die Schmerzen von Personen mit Kniearthrose besser senken als der Einsatz einer Total-Endoprothese?

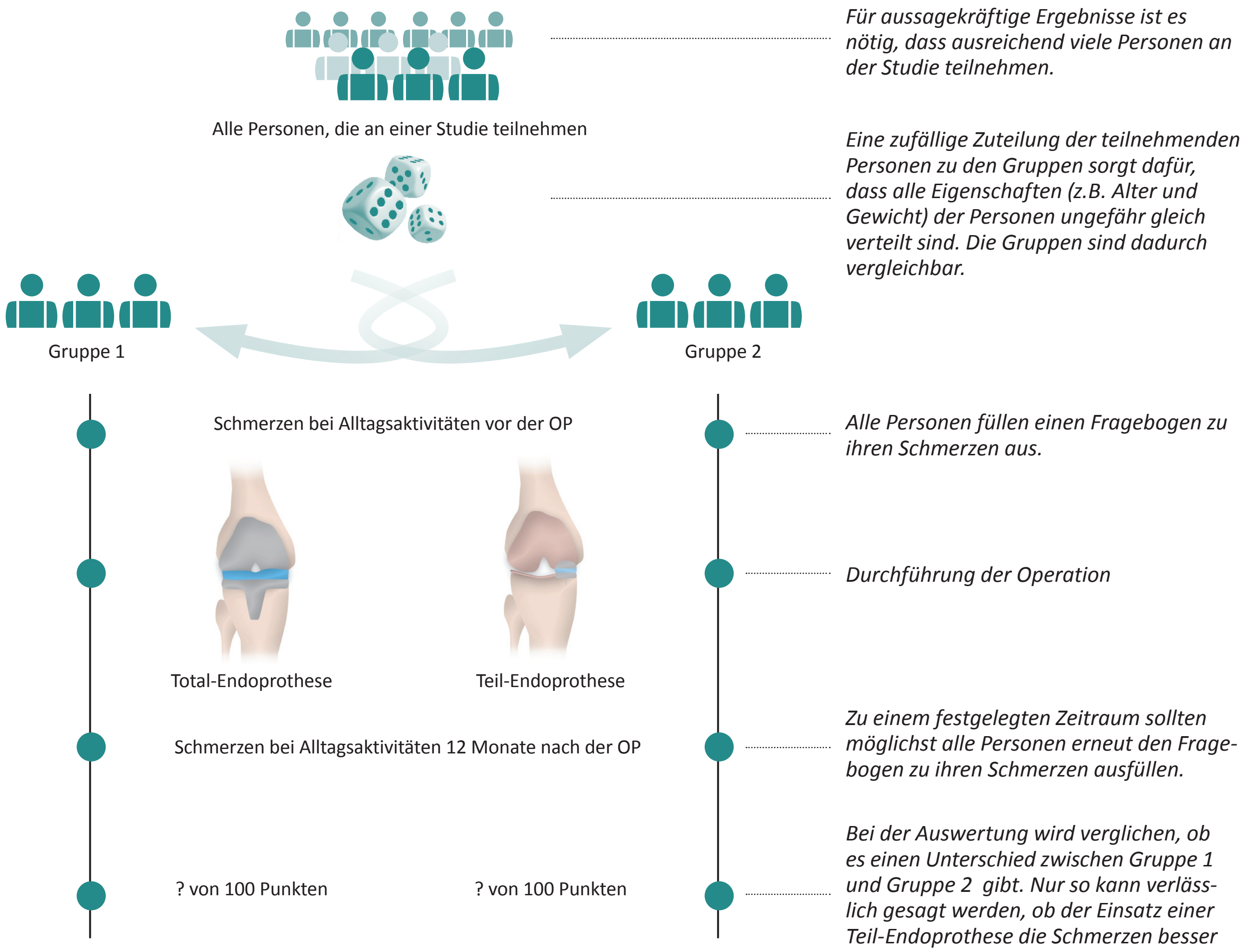

Abbildung 10: Beispiel einer randomisiert-kontrollierten Studie

Welche Sicherheiten und Unsicherheiten sind mit den Zahlen verbunden?

Zahlen vermitteln den Eindruck von Genauigkeit. Tatsächlich sind sie mit vielen Unsicherheiten verbunden. Zahlen als Ergebnisse wissenschaftlicher Studien sind nur Schätzwerte. Wie genau die Zahlen geschätzt werden, hängt zum Beispiel davon ab, wie groß eine Studie ist. Außerdem handelt es sich um Wahrscheinlichkeiten. Für die einzelne Person lassen sich keine sicheren Vorhersagen treffen.

Sind die Zahlen dieser Information vollständig auf mich übertragbar?

An den Studien, die für die Erstellung der Broschüre verwendeten wurden, nahmen auch Menschen teil, die sich hinsichtlich der Erkrankung und möglicher Nebenerkrankungen von der Zielgruppe dieser Information unterscheiden. Es ist daher möglich, dass sich die Ergebnisse nicht vollständig auf Sie übertragen lassen. Wenn Sie wissen wollen, ob sich die Studienergebnisse auf Ihre Situation übertragen lassen, sprechen Sie bitte mit Ihrer Ärztin oder Ihrem Arzt darüber.

Welche Vor- und Nachteile haben die verschiedenen Operationsmöglichkeiten?

Wie können die Ergbnisse gelesen werden?

Die Teilnehmenden füllten vor und nach der Behandlung einen Fragebogen zu verschiedenen Lebensbereichen aus. Anhand der Punkte können Sie sich ein Bild davon machen, welche Verbesserungen zum Beispiel beim Gehen und Treppensteigen einen Monat nach der Operation zu erwarten sind. Die folgende Abbildung hilft Ihnen, die dargestellten Ergebnisse zu verstehen.

Lebensbereiche, zu denen die Studienteilnehmenden befragt wurden.

Skala von 0 – 100 Punkten
0 = keine Funktionsfähigkeit 100 = volle Funktionsfähigkeit
Sie können sich die Einteilung wie den Ladestand eines Akkus vorstellen. 100 steht für „volle Energie", das heißt volle Funktionsfähigkeit, oder Schmerzfreiheit, oder vollstes Wohlbefinden.

Auf Seite 33 finden Sie einen Link zu allen Quellen.

Total-Endoprothese mit oder ohne minimalinvasiven Zugang [11]

Nutzen einer Total-Endoprothese mit Standard- und minimalinvasivem Zugang im Vergleich

Alltagsaktivitäten

100 Punkte = volle Funktionsfähigkeit

vor OP — 36-59 von 100 Punkten — 9 (Studien) — 738 (Teilnehmende)

Ausgangswert der Teilnehmenden vor der OP

1 Monat nach OP

bessere Funktionsfähigkeit mit minimalinvasivem Zugang

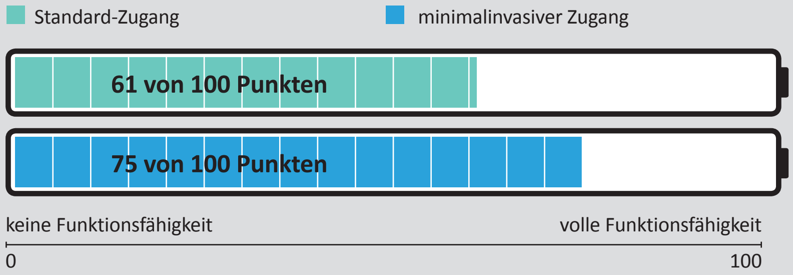

2 (Studien) — 150 (Teilnehmende)

keine Unterschiede zwischen den Verfahren

6 Wochen nach OP — 62 – 72 von 100 Punkten — 3 (Studien) — 301 (Teilnehmende)

__Unterschiede__ zwischen den Verfahren werden als Balkendiagramm abgebildet. Es ist die in einem Fragebogen durchschnittlich angegebene Punktzahl der Teilnehmenden abgebildet

__Keine__ Unterschiede zwischen den Verfahren werden als minimale und maximale durchschnittliche Punktzahl abgebildet.

Anzahl der Studien, die in die Darstellung der Ergebnisse eingeflossen sind.

Anzahl der Teilnehmenden, die in den Studien beobachtet wurden.

Die Operationsmöglichkeiten auf einen Blick

Teil-Endoprothese 12

Total-Endoprothese ...

mit verschiedenen Kopplungsgraden 14
mit oder ohne Ersatz der Kniescheibenrückseite 16
mit unbeweglicher oder beweglicher Kunststoffgleitfläche 18
mit oder ohne zementierte Befestigung 22
mit oder ohne computergestützter Navigation 23
mit oder ohne patientenindividuellen Instrumenten 25
mit oder ohne minimalinvasiven Zugang 28
mit oder ohne robotergestützte Navigation 30

Teil-Endoprothese [4]

Nutzen einer Total-Endoprothese (TEP) und Teil-Endoprothese im Vergleich

Zahlen sind Schätzungen von Wahrscheinlichkeiten und lassen keine konkreten Vorhersagen für eine Person zu. Fragen Sie Ihr Behandlungsteam, wenn Sie sich unsicher sind, ob sich die Ergebnisse auf Ihre Situation übertragen lassen.

i

Alltagsaktivitäten und Schmerzen

100 Punkte = volle Funktionsfähigkeit

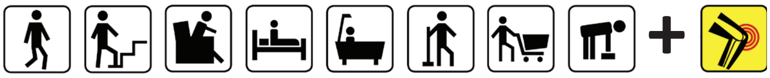

vor OP	19 von 100 Punkten	1	528
2 Monate nach OP	**keine Unterschiede zwischen den Verfahren**<br>29 – 32 von 100 Punkten	1	486
12, 24 Monate, 3, 4 und 5 Jahre nach OP	**keine Unterschiede zwischen den Verfahren**<br>35 – 39 von 100 Punkten	1	464

Interpretation der Ergebnisse

Mithilfe eines Fragebogens wurden die Funktionsfähigkeit und Schmerzen bei verschiedenen Alltagsaktivitäten vor der OP und 2 Monate sowie 12, 24 Monate, 3,4 und 5 Jahre nach der OP erhoben. Beispielhaft wird im Folgenden erklärt, wie die Ergebnisse vor der OP und zum Zeitpunkt nach 2 Monaten nach der OP gelesen werden können.

Vor der Operation haben sowohl die Personen, die eine Knie-TEP erhalten werden, als auch die Personen, die eine Teil-Endoprothese erhalten werden, ihre Funktionsfähigkeit und Schmerzen bei Alltagsaktivitäten durchschnittlich mit 19 von 100 Punkten bewertet. Dabei bedeuten 0 keine Funktionsfähigkeit und stärkste Schmerzen und 100 volle Funktionsfähigkeit und Schmerzfreiheit.

2 Monate nach der OP bewerteten die Teilnehmenden aus beiden Gruppen (Knie-TEP und Teil-Endoprothese) ihre Funktionsfähigkeit und Schmerzen bei Alltagsaktivitäten durchschnittlich zwischen 29 und 31 von 100 Punkten.

Im Vergleich zum Wert vor der Behandlung (19), gaben sowohl Teilnehmende nach einer Knie-TEP als auch Teilnehmende nach einer Teil-Endoprothese eine bessere Funktionsfähigkeit und weniger Schmerzen bei Alltagsaktivitäten nach der OP an (29-31). Es besteht kein Unterschied zwischen den Verfahren hinsichtlich Funktionsfähigkeit und Schmerzen bei Alltagsaktivitäten.

Diese Ergebnisse basieren auf 1 Studie mit 528 Teilnehmenden. Nach 2 Monaten haben noch 486 der ursprünglich 528 Teilnehmenden den Fragebogen ausgefüllt.

Lebensqualität

100 Punkte = vollstes Wohlbefinden

vor OP	61 – 63 Punkten	1 506
12, 24 Monate, 3, 4 und 5 Jahre nach OP	**keine Unterschiede zwischen den Verfahren** 73 – 76 von 100 Punkten	1 424
5 Jahre nach OP	**Hinweise auf höheres Wohlbefinden mit Teilprothese unklar, ob der geringe Unterschied zwischen den Werten wahrnehmbar ist**	1 445

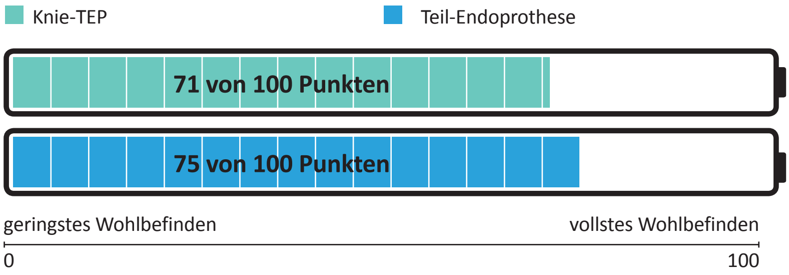

Sportliche Aktivität

keine Erkenntnisse aus Studien vorliegend

Komplikationen einer Total-Endoprothese (TEP) und Teil-Endoprothese im Vergleich

In randomisiert-kontrollierten Studien, die den Nachweis erbringen können, ob eine Behandlung besser als eine andere ist (siehe Seite 10), zeigten sich keine Unterschiede hinsichtlich der Häufigkeit von Komplikationen.

Zusätzlich haben wir Daten aus sogenannten Registerstudien angeschaut. In manchen Ländern werden große Datenbanken zu den eingebauten Knie-Endoprothesen angelegt. Dabei wird neben den Details zur Operation auch erfasst, ob Prothesenteile ausgetauscht werden mussten.

In Daten von 176.569 Operationen konnten Hinweise gefunden werden, dass mit einer Total-Endoprothese ein geringerer Anteil an Prothesen ausgetauscht werden musste, als mit einer Teil-Endoprothese. Die Personen wurden zwischen 5 und 8 Jahren beobachtet.

Aufgrund methodischer Einschränkungen von Registerstudien kann nicht eindeutig gesagt werden, ob der geringere Anteil an ausgetauschten Prothesenteilen tatsächlich auf die Total-Endoprothese zurückzuführen ist oder unterschiedliche Ausgangsvoraussetzungen ursächlich sind.

Eine ausführliche Übersicht zu den Komplikationen einer Total-Endoprothese finden Sie im Aufklärungsbogen auf den Seiten 14-20.

Total-Endoprothese mit verschiedenen Kopplungsgraden [5]

Nutzen einer ungekoppelten und stabilisierten Total-Endoprothese im Vergleich

Zahlen sind Schätzungen von Wahrscheinlichkeiten und lassen keine konkreten Vorhersagen für eine Person zu. Fragen Sie Ihr Behandlungsteam, wenn Sie sich unsicher sind, ob sich die Ergebnisse auf Ihre Situation übertragen lassen.

i

Alltagsaktivitäten

100 Punkte = volle Funktionsfähigkeit

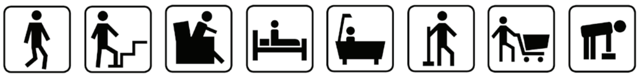

vor OP	43 – 56 von 100 Punkten	4	324
3 Monate nach OP	**keine Unterschiede zwischen den Verfahren**<br>76 von 100 Punkten	3	252
6 Monate nach OP	**keine Unterschiede zwischen den Verfahren**<br>79 – 80 von 100 Punkten	2	173
12 Monate nach OP	**keine Unterschiede zwischen den Verfahren**<br>79 – 81 von 100 Punkten	3	213
24 Monate nach OP	**keine Unterschiede zwischen den Verfahren**<br>80 von 100 Punkten	2	138

Schmerzen

100 Punkte = Schmerzfreiheit

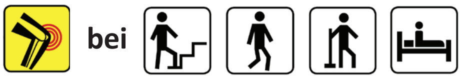

vor OP	37 – 51 von 100 Punkten	4	324
3 Monate nach OP	**keine Unterschiede zwischen den Verfahren**<br>76 – 79 von 100 Punkten	3	252
6 Monate nach OP	**keine Unterschiede zwischen den Verfahren**<br>82 von 100 Punkten	2	173
12 Monate nach OP	**keine Unterschiede zwischen den Verfahren**<br>82 – 83 von 100 Punkten	3	213
24 Monate nach OP	**keine Unterschiede zwischen den Verfahren**<br>85 – 86 von 100 Punkten	2	138

Lebensqualität

100 Punkte = vollstes Wohlbefinden

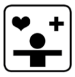

3, 12 und 24 Monate nach OP	**keine Unterschiede zwischen den Verfahren Hinweise auf höheres Wohlbefinden nach beiden Verfahren**	1	47

Sportliche Aktivitäten

100 Punkte = volle Funktionsfähigkeit

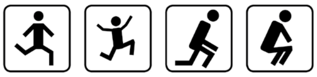

3, 12 und 24 Monate nach OP	**keine Unterschiede zwischen den Verfahren Hinweise auf bessere Funktionsfähigkeit nach beiden Verfahren**	1	47

Komplikationen einer ungekoppelten und stabilisierten Total-Endoprothese im Vergleich

In randomisiert-kontrollierten Studien, die den Nachweis erbringen können, ob eine Behandlung besser als eine andere ist (siehe Seite 10), zeigten sich keine Unterschiede hinsichtlich der Häufigkeit von Komplikationen.

Zusätzlich haben wir Daten aus sogenannten Registerstudien angeschaut. In manchen Ländern werden große Datenbanken zu den eingebauten Knie-Endoprothesen angelegt. Dabei wird neben den Details zur Operation auch erfasst, ob Prothesenteile ausgetauscht werden mussten.

In Daten von 639.139 Operationen konnten Hinweise gefunden werden, dass bei ungekoppelten Total-Endoprothesen ein geringerer Anteil an Prothesen ausgetauscht werden musste, als bei stabilisierten Prothesen. Die Personen wurden 2 Jahre beobachtet.

Aufgrund methodischer Einschränkungen von Registerstudien kann nicht eindeutig gesagt werden, ob der geringere Anteil an ausgetauschten Prothesenteilen tatsächlich auf die ungekoppelte Total-Endoprothese zurückzuführen ist oder unterschiedliche Ausgangsvoraussetzungen ursächlich sind.

Eine ausführliche Übersicht zu den Komplikationen einer Total-Endoprothese finden Sie im Aufklärungsbogen auf den Seiten 14-20.

Total-Endoprothese mit oder ohne Ersatz der Kniescheibenrückseite [6]

Nutzen einer Total-Endoprothese ohne und mit Ersatz der Kniescheibenrückseite im Vergleich

Zahlen sind Schätzungen von Wahrscheinlichkeiten und lassen keine konkreten Vorhersagen für eine Person zu. Fragen Sie Ihr Behandlungsteam, wenn Sie sich unsicher sind, ob sich die Ergebnisse auf Ihre Situation übertragen lassen.

i

Alltagsaktivitäten

100 Punkte = volle Funktionsfähigkeit

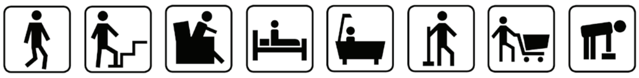

4 und 6 Monate nach OP	**keine Unterschiede zwischen den Verfahren**<br>**Hinweise auf bessere Funktionsfähigkeit**<br>**nach beiden Verfahren**	2	92

Schmerzen

100 Punkte = Schmerzfreiheit

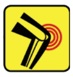

vor OP	13 – 45 von 100 Punkten	3	174
4 und 6 Monate nach OP	**keine Unterschiede zwischen den Verfahren**<br>84 von 100 Punkten	2	92
12 und 24 Monate nach OP	**keine Unterschiede zwischen den Verfahren**<br>**Hinweise auf weniger Schmerzen**<br>**nach beiden Verfahren**	2	111

Lebensqualität

100 Punkte = vollstes Wohlbefinden

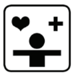

vor OP	**geistiges Wohlbefinden**<br>50 – 51 von 100 Punkten<br>**körperliches Wohlbefinden**<br>31 von 100 Punkten	1	1715
12, 24 Monate,<br>3, 4 und 5 Jahre nach OP	**keine Unterschiede zwischen den Verfahren**<br>**geistiges Wohlbefinden**<br>50 – 52 von 100 Punkten<br>**körperliches Wohlbefinden**<br>39 – 41 von 100 Punkten	1	1310

Sportliche Aktivitäten

100 Punkte = volle Funktionsfähigkeit

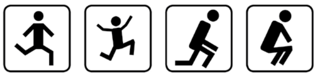

12 Monate nach OP	**keine Unterschiede zwischen den Verfahren**<br>**Hinweise auf bessere Funktionsfähigkeit nach beiden Verfahren**	1 71

Komplikationen einer ungekoppelten und stabilisierten Total-Endoprothese im Vergleich

In randomisiert-kontrollierten Studien, die den Nachweis erbringen können, ob eine Behandlung besser als eine andere ist (siehe Seite 10), zeigten sich keine Unterschiede hinsichtlich der Häufigkeit von Komplikationen.

Zusätzlich haben wir Daten aus sogenannten Registerstudien angeschaut. In manchen Ländern werden große Datenbanken zu den eingebauten Knie-Endoprothesen angelegt. Dabei wird neben den Details zur Operation auch erfasst, ob Prothesenteile ausgetauscht werden mussten.

In Daten von 898.032 Operationen konnten Hinweise gefunden werden, dass bei Total-Endoprothesen ohne Ersatz der Kniescheibenrückseite ein größerer Anteil an Prothesen ausgetauscht werden musste, als bei Prothesen bei denen ein Ersatz erfolgte. Die Personen wurden zwischen 2 und 5 Jahren beobachtet.

Aufgrund methodischer Einschränkungen von Registerstudien kann nicht eindeutig gesagt werden, ob der größere Anteil an ausgetauschten Prothesenteilen tatsächlich auf den fehlenden Ersatz der Kniescheibenrückseite zurückzuführen ist oder unterschiedliche Ausgangsvoraussetzungen ursächlich sind.

Eine ausführliche Übersicht zu den Komplikationen einer Total-Endoprothese finden Sie im Aufklärungsbogen auf den Seiten 14-20.

Total-Endoprothese mit unbeweglicher oder beweglicher Kunststoffgleitfläche [7]

Nutzen einer Total-Endoprothese mit unbeweglicher und beweglicher Kunststoffgleitfläche im Vergleich

Zahlen sind Schätzungen von Wahrscheinlichkeiten und lassen keine konkreten Vorhersagen für eine Person zu. Fragen Sie Ihr Behandlungsteam, wenn Sie sich unsicher sind, ob sich die Ergebnisse auf Ihre Situation übertragen lassen.

Alltagsaktivitäten

100 Punkte = volle Funktionsfähigkeit

vor OP	25 – 62 von 100 Punkten	12	1736
6 Wochen nach OP	**Hinweise auf auf bessere Funktionsfähigkeit mit unbeweglicher Kunststoffgleitfläche**	1	50
3 Monate nach OP	**bessere Funktionsfähigkeit mit unbeweglicher Kunststoffgleitfläche** (siehe Grafik)	2	142
6 Monate nach OP	**keine Unterschiede zwischen den Verfahren** 83 – 84 von 100 Punkten	2	142
12 Monate nach OP	**keine Unterschiede zwischen den Verfahren** 74 – 75 von 100 Punkten	6	946
24 Monate nach OP	**keine Unterschiede zwischen den Verfahren bessere Funktionsfähigkeit nach beiden Verfahren**	2	599
3,5 und 4 Jahre nach OP	**keine Unterschiede zwischen den Verfahren** 74 – 77 von 100 Punkten	2	452

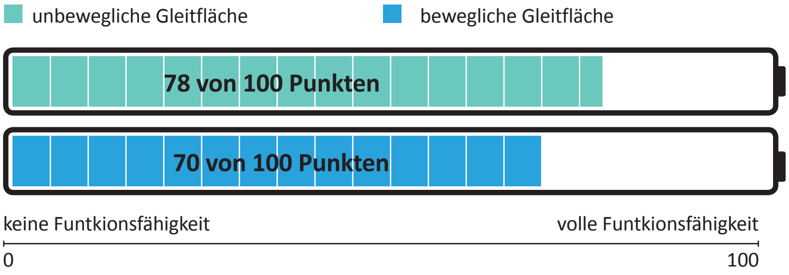

Schmerzen

100 Punkte = Schmerzfreiheit

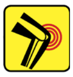

vor OP	25 – 47 von 100 Punkten	3	183

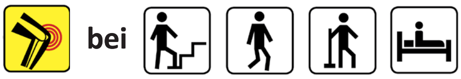

Hinweise auf weniger Schmerzen in Ruhe mit unbeweglicher Kunststoffgleitfläche nach 3 Monaten mit einem Fragebogen

unklar, ob der geringe Unterschied zwischen den Werten wahrnehmbar ist

keine Hinweise auf Unterschied zwischen den Verfahren mit anderem Fragebogen

3 Monate nach OP	**Hinweise auf weniger Schmerzen nach beiden Verfahren**	2	190

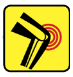

12 Monate nach OP	**keine Unterschiede zwischen den Verfahren**<br>79 – 82 von 100 Punkten	2	150

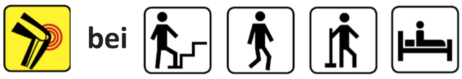

24 Monate nach OP	**keine Unterschiede zwischen den Verfahren**<br>**Hinweise auf weniger Schmerzen mit beiden Verfahren**	4	554

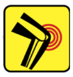

5 Jahre nach OP	**keine Unterschiede zwischen den Verfahren**<br>**Hinweise auf weniger Schmerzen mit beiden Verfahren**	2	106

Lebensqualität

100 Punkte = vollstes Wohlbefinden

vor OP	**geistiges Wohlbefinden**<br>48 – 50 von 100 Punkten<br>**körperliches Wohlbefinden**<br>30 – 31 von 100 Punkten	2	785
3 Monate nach OP	**keine Unterschiede zwischen den Verfahren**<br>**geistiges Wohlbefinden**<br>48 – 49 von 100 Punkten<br>**körperliches Wohlbefinden**<br>38 – 39 von 100 Punkten	1	456
6 Monate nach OP	**keine Erkenntnisse aus Studien vorliegend**		
12 und 24 Monate nach OP	**keine Unterschiede zwischen den Verfahren**<br>**geistiges Wohlbefinden**<br>50 – 51 von 100 Punkten<br>**körperliches Wohlbefinden**<br>39 – 41 von 100 Punkten	2	705
3, 4 und 5 Jahre nach OP	**keine Unterschiede zwischen den Verfahren**<br>**geistiges Wohlbefinden**<br>48 – 50 von 100 Punkten<br>**körperliches Wohlbefinden**<br>38 – 39 von 100 Punkten	1	404

Sportliche Aktivität

keine Erkenntnisse aus Studien vorliegend

Komplikationen einer Total-Endoprothese mit unbeweglicher und beweglicher Kunststoffgleitfläche im Vergleich

**Komplikation**	**Total-Endoprothese mit unbeweglicher und beweglicher Kunststoffgleitfläche im Vergleich**
**Gelenkinfektionen**<br>können schwerwiegende Folgen haben und sind mit erheblichen Bewegungseinschränkungen verbunden. Es können langwierige Behandlungen und ein Austausch der Prothese nötig werden.	**weniger Gelenkinfektionen mit unbeweglicher Kunststoffgleitfläche**

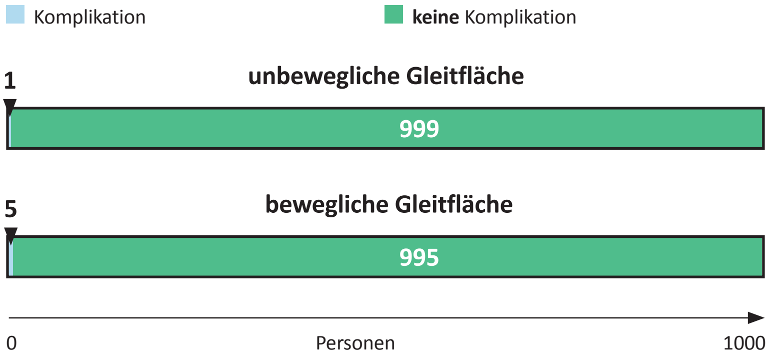

8 1378

Es zeigten sich keine weiteren Unterschiede in randomisiert-kontrollierten Studien, die den Nachweis erbringen können, ob eine Behandlung besser als eine andere ist (siehe Seite 10).

Zusätzlich haben wir Daten aus sogenannten Registerstudien angeschaut. In manchen Ländern werden große Datenbanken zu den eingebauten Knie-Endoprothesen angelegt. Dabei wird neben den Details zur Operation auch erfasst, ob Prothesenteile ausgetauscht werden mussten.

In Daten von 958.843 Operationen konnten Hinweise gefunden werden, dass bei Total-Endoprothesen mit unbeweglicher Gleitfläche ein geringerer Anteil an Prothesen ausgetauscht werden musste, als bei Prothesen mit beweglicher Gleitfläche. Die Personen wurden zwischen 2 und 10 Jahren beobachtet.

Aufgrund methodischer Einschränkungen von Registerstudien kann nicht eindeutig gesagt werden, ob der geringere Anteil an ausgetauschten Prothesenteilen tatsächlich auf die unbewegliche Gleitfläche zurückzuführen ist oder unterschiedliche Ausgangsvoraussetzungen ursächlich sind.

Eine ausführliche Übersicht zu den Komplikationen einer Total-Endoprothese finden Sie im Aufklärungsbogen auf den Seiten 14-20

Wie können die Ergbnisse gelesen werden?

Für die Komplikationen wurden mehrere Studienergebnisse zusammengefasst. Bei der Häufigkeit von Gelenkinfektionen konnten Unterschiede zwischen den Verfahren festgestellt werden. Die folgende Abbildung hilft Ihnen, die dargestellten Ergebnisse zu verstehen.

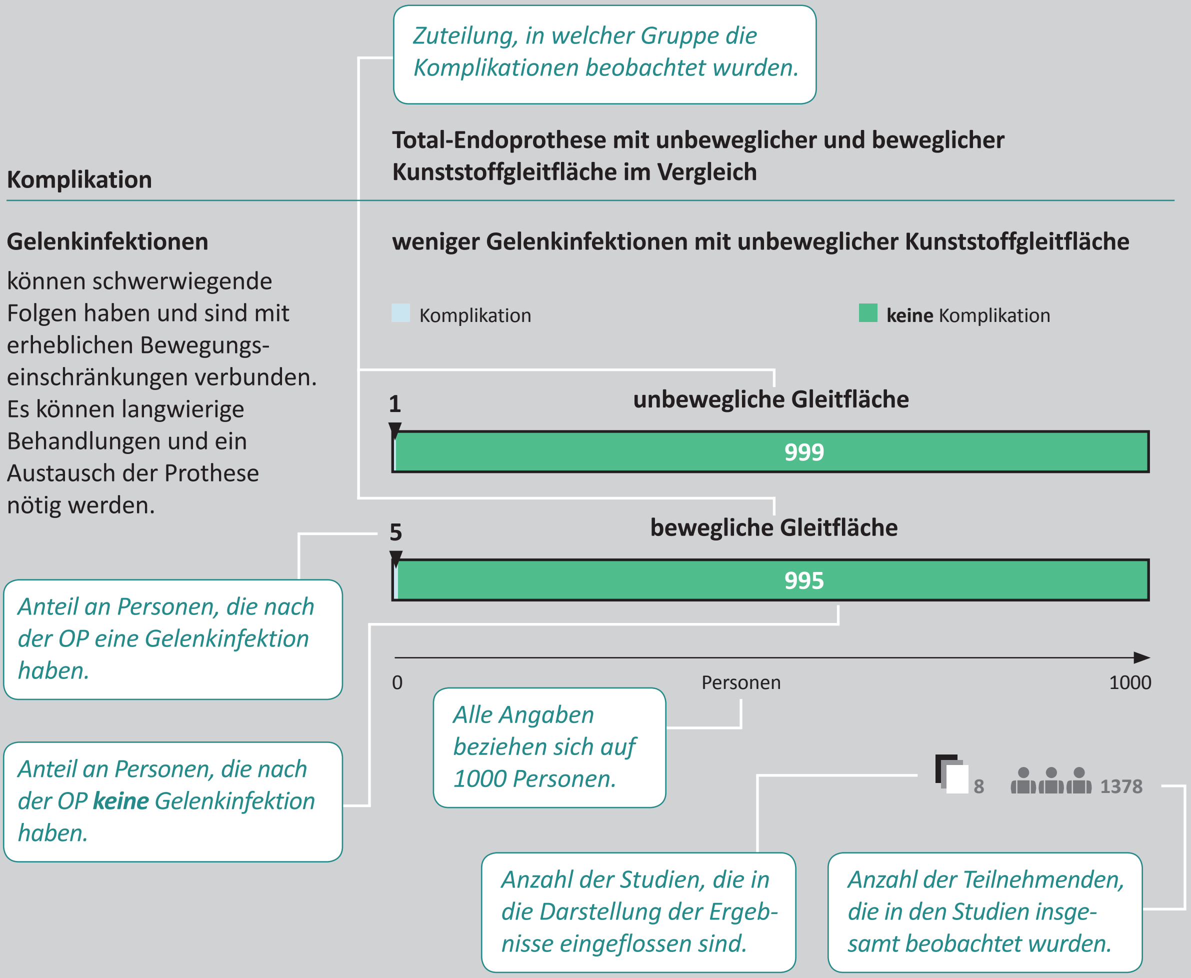

Total-Endoprothese mit oder ohne zementierte Befestigung [8]

Nutzen einer Total-Endoprothese mit zementierter und nicht zementierter Befestigung im Vergleich

Zahlen sind Schätzungen von Wahrscheinlichkeiten und lassen keine konkreten Vorhersagen für eine Person zu. Fragen Sie Ihr Behandlungsteam, wenn Sie sich unsicher sind, ob sich die Ergebnisse auf Ihre Situation übertragen lassen.

i

Alltagsaktivitäten und Schmerzen

100 Punkte = volle Funktionsfähigkeit

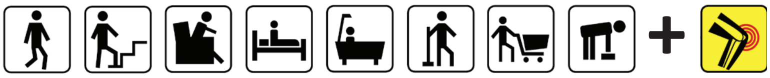

vor OP	41 – 49 von 100 Punkten	2	231
24 Monate nach OP	**keine Unterschiede zwischen den Verfahren**<br>88 von 100 Punkten	2	220

Lebensqualität

keine Erkenntnisse aus Studien vorliegend

Sportliche Aktivität

keine Erkenntnisse aus Studien vorliegend

Komplikationen einer Total-Endoprothese mit zementierter und nicht zementierter Befestigung im Vergleich

In randomisiert-kontrollierten Studien, die den Nachweis erbringen können, ob eine Behandlung besser als eine andere ist (siehe Seite 10), zeigten sich keine Unterschiede hinsichtlich der Häufigkeit von Komplikationen.

Zusätzlich haben wir Daten aus sogenannten Registerstudien angeschaut. In diesen Studien zeigten sich ebenso keine Unterschiede.

Eine ausführliche Übersicht zu den Komplikationen einer Total-Endoprothese finden Sie im Aufklärungsbogen auf den Seiten 14-20.

Total-Endoprothese mit oder ohne computergestützter Navigation [9]

Nutzen einer Total-Endoprothese mit Standard- und computergestützter Navigation im Vergleich

> *i* Zahlen sind Schätzungen von Wahrscheinlichkeiten und lassen keine konkreten Vorhersagen für eine Person zu. Fragen Sie Ihr Behandlungsteam, wenn Sie sich unsicher sind, ob sich die Ergebnisse auf Ihre Situation übertragen lassen.

Alltagsaktivitäten

100 Punkte = volle Funktionsfähigkeit

		Studien	Teilnehmende
vor OP	27 – 61 von 100 Punkten	5	623
3 Monate nach OP	**Hinweise auf bessere Funktionsfähigkeit nach computergestützter OP beim Gehen und Treppensteigen**<br>**unklar, ob der geringe Unterschied zwischen den Werten wahrnehmbar ist**<br>**keine Unterschiede zwischen Verfahren in anderen Lebensbereichen**<br>**bessere Funktionsfähigkeit nach beiden Verfahren**	1	190
6 Monate nach OP	**keine Unterschiede zwischen den Verfahren**<br>72 – 74 von 100 Punkten	3	356
12 Monate nach OP	**keine Unterschiede zwischen den Verfahren**<br>74 – 75 von 100 Punkten	2	216
24 Monate nach OP	**keine Unterschiede zwischen den Verfahren**<br>76 – 78 von 100 Punkten	4	430
4 Jahre nach OP	**keine Unterschiede zwischen den Verfahren**<br>**Hinweise auf bessere Funktionsfähigkeit nach beiden Verfahren**	1	50

Schmerzen

100 Punkte = Schmerzfreiheit

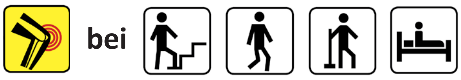

	**Hinweise mit einem Fragebogen auf weniger Schmerzen nach computergestützter OP**<br>**unklar, ob der geringe Unterschied zwischen den Werten wahrnehmbar ist**<br>**keine Hinweise auf Unterschied zwischen Verfahren mit anderem Fragebogen**		
3 Monate nach OP	**Hinweise auf weniger Schmerzen nach beiden Verfahren**	2	233
6 Monate nach OP	**keine Erkenntnisse aus Studien vorliegend**		
12 Monate nach OP	**keine Unterschiede zwischen den Verfahren**<br>**Hinweise auf weniger Schmerzen nach beiden Verfahren**	1	173
24 Monate nach OP	**keine Unterschiede zwischen den Verfahren**<br>**Hinweise auf weniger Schmerzen nach beiden Verfahren**	1	167

Lebensqualität

100 Punkte = vollstes Wohlbefinden

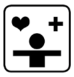

3, 6, 12 und 24 Monate nach OP	**keine Unterschiede zwischen den Verfahren**<br>**Hinweise auf höheres Wohlbefinden nach beiden Verfahren**	3	380

Sportliche Aktivitäten

100 Punkte = volle Funktionsfähigkeit

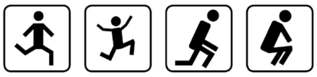

	**Hinweise auf bessere Funktionsfähigkeit nach computergestützter OP nach 3 und 24 Monaten**<br>**keine Unterschiede zwischen den Verfahren 12 Monate nach der OP**		
bis 24 Monate nach OP	**Hinweise auf bessere Funktionsfähigkeit nach beiden Verfahren**	1	167

Komplikationen einer Total-Endoprothese mit Standard- und computergestützter Navigation im Vergleich

In randomisiert-kontrollierten Studien, die den Nachweis erbringen können, ob eine Behandlung besser als eine andere ist (siehe Seite 10), zeigten sich keine Unterschiede hinsichtlich der Häufigkeit von Komplikationen.

Zusätzlich haben wir Daten aus sogenannten Registerstudien angeschaut. In diesen Studien zeigten sich ebenso keine Unterschiede.

Eine ausführliche Übersicht zu den Komplikationen einer Total-Endoprothese finden Sie im Aufklärungsbogen auf den Seiten 14-20.

Total-Endoprothese mit oder ohne patientenindividuellen Instrumenten [10]

Nutzen einer Total-Endoprothese mit Standardinstrumenten und patientenindividuellen Instrumenten im Vergleich

> **i** Zahlen sind Schätzungen von Wahrscheinlichkeiten und lassen keine konkreten Vorhersagen für eine Person zu. Fragen Sie Ihr Behandlungsteam, wenn Sie sich unsicher sind, ob sich die Ergebnisse auf Ihre Situation übertragen lassen.

Alltagsaktivitäten

100 Punkte = volle Funktionsfähigkeit

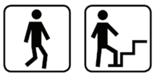

vor OP

18 – 63 von 100 Punkten

6 492

3 Monate nach OP

keine Unterschiede zwischen den Verfahren

74 – 75 von 100 Punkten

4 459

12 Monate nach OP

bessere Funktionsfähigkeit mit
mit Standardinstrumenten

unklar, ob der geringe Unterschied zwischen den Werten wahrnehmbar ist

Standardinstrumente   individuelle Instrumente

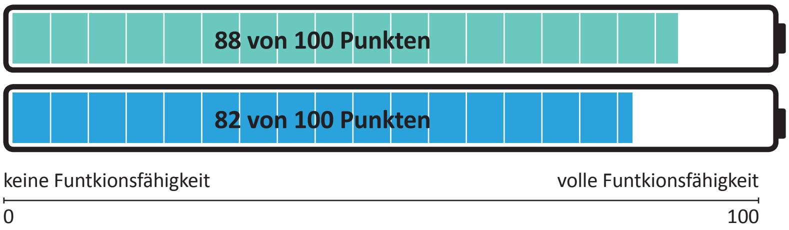

3 173

12 und 24 Monate nach OP

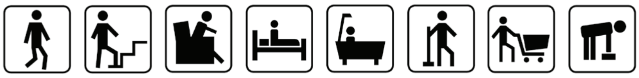

bessere Funktionsfähigkeit mit
mit individuellen Instrumenten

unklar, ob der geringe Unterschied zwischen den Werten wahrnehmbar ist

Standardinstrumente   individuelle Instrumente

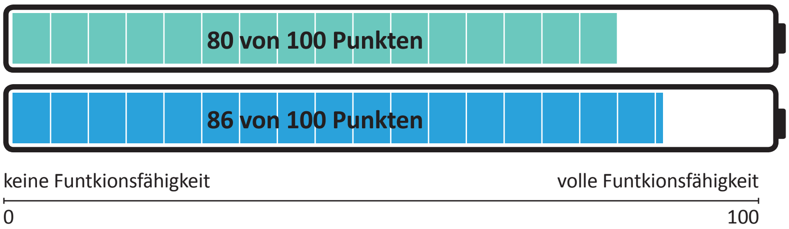

3 170

Schmerzen

100 Punkte = Schmerzfreiheit

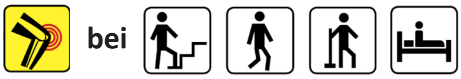

vor OP | 37 – 55 von 100 Punkten | 2 | 136

12 und 24 Monate nach OP

schmerzfreier mit individuellen Instrumenten

unklar, ob der geringe Unterschied zwischen den Werten wahrnehmbar ist

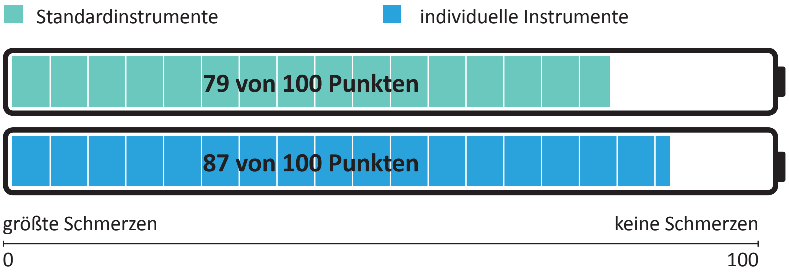

3 | 170

Lebensqualität

100 Punkte = vollstes Wohlbefinden

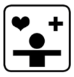

keine Unterschiede zwischen den Verfahren

vor OP | 21 – 24 von 100 Punkten | 2 | 136

12 und 24 Monate nach OP

höheres Wohlbefinden mit individuellen Instrumenten

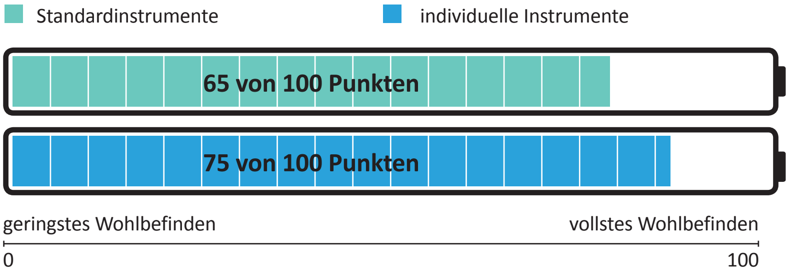

3 | 170

3 und 24 Monate nach OP

keine Unterschiede zwischen den Verfahren mit anderem Fragebogen

Hinweise auf höheres Wohlbefinden nach beiden Verfahren

2 | 274

Sportliche Aktivitäten

100 Punkte = volle Funktionsfähigkeit

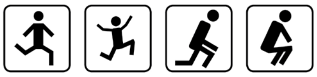

vor OP	**keine Unterschiede zwischen den Verfahren**<br>9 – 15 von 100 Punkten	2	136
12 und 24 Monate nach OP	**keine Unterschiede zwischen den Verfahren**<br>47 – 51 von 100 Punkten	3	170

Komplikationen einer Total-Endoprothese mit Standardinstrumenten und patientenindividuellen Instrumenten im Vergleich

In randomisiert-kontrollierten Studien, die den Nachweis erbringen können, ob eine Behandlung besser als eine andere ist (siehe Seite 10), zeigten sich keine Unterschiede hinsichtlich der Häufigkeit von Komplikationen.

Zusätzlich haben wir Daten aus sogenannten Registerstudien angeschaut. In diesen Studien zeigten sich ebenso keine Unterschiede.

Eine ausführliche Übersicht zu den Komplikationen einer Total-Endoprothese finden Sie im Aufklärungsbogen auf den Seiten 14-20.

Total-Endoprothese mit oder ohne minimalinvasiven Zugang (11)

Nutzen einer Total-Endoprothese mit Standard- und minimalinvasivem Zugang im Vergleich

> Zahlen sind Schätzungen von Wahrscheinlichkeiten und lassen keine konkreten Vorhersagen für eine Person zu. Fragen Sie Ihr Behandlungsteam, wenn Sie sich unsicher sind, ob sich die Ergebnisse auf Ihre Situation übertragen lassen.
>
> *i*

Alltagsaktivitäten

100 Punkte = volle Funktionsfähigkeit

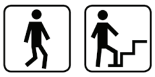

keine Unterschiede zwischen den Verfahren

vor OP — 36 – 59 von 100 Punkten — 9 — 738

1 Monat nach OP

bessere Funktionsfähigkeit mit minimalinvasivem Zugang

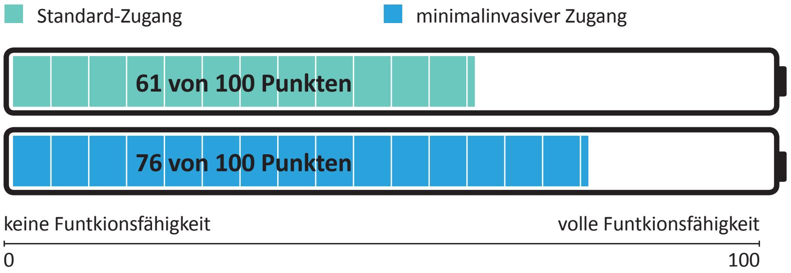

2 — 150

6 Wochen nach OP — **keine Unterschiede zwischen den Verfahren** — 62 – 72 von 100 Punkten — 3 — 301

3 Monate nach OP — **keine Unterschiede zwischen den Verfahren** — 82 – 89 von 100 Punkten — 2 — 170

12 Monate nach OP — **keine Unterschiede zwischen den Verfahren** — 86 – 88 von 100 Punkten — 4 — 381

Schmerzen

100 Punkte = Schmerzfreiheit

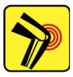

bis 6 Wochen nach OP	**Hinweise auf weniger Schmerzen unmittelbar nach OP mit minimalinvasivem Zugang**<br>**unklar, ob der geringe Unterschied zwischen den Werten wahrnehmbar ist**	2	184

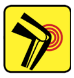 **bei** 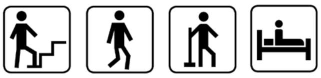

3 Monate nach OP	**keine Unterschiede zwischen den Verfahren**<br>**Hinweise auf weniger Schmerzen nach beiden Verfahren**	1	36

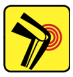

6 Monate nach OP	**Hinweise auf weniger Schmerzen mit minimalinvasivem Zugang**<br>**unklar, ob der geringe Unterschied zwischen den Werten wahrnehmbar ist**	1	50

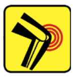 **bei** 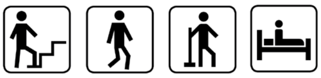

12 Monate nach OP	**keine Unterschiede zwischen den Verfahren**<br>**Hinweise auf weniger Schmerzen nach beiden Verfahren**	2	184

Lebensqualität

100 Punkte = vollstes Wohlbefinden

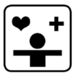

2 Monate nach OP	**keine Unterschiede zwischen den Verfahren**<br>**Hinweise auf höheres Wohlbefinden nach beiden Verfahren**	1	36

Sportliche Aktivitäten

100 Punkte = volle Funktionsfähigkeit

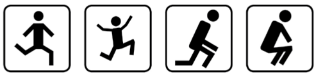

2 Monate nach OP	**keine Unterschiede zwischen den Verfahren**<br>**Hinweise auf bessere Funktionsfähigkeit nach beiden Verfahren**	1	36

Komplikationen einer Total-Endoprothese mit Standard- und minimalinvasivem Zugang im Vergleich

In randomisiert-kontrollierten Studien, die den Nachweis erbringen können, ob eine Behandlung besser als eine andere ist (siehe Seite 10), zeigten sich keine Unterschiede hinsichtlich der Häufigkeit von Komplikationen.

Zusätzliche Daten aus sogenannten Registerstudien sind nicht verfügbar.

Eine ausführliche Übersicht zu den Komplikationen einer Total-Endoprothese finden Sie im Aufklärungsbogen auf den Seiten 14-20.

Total-Endoprothese mit oder ohne robotergestützte Navigation [12]

Nutzen einer Total-Endoprothese mit Standard- und robotergestützter Navigation im Vergleich

> Zahlen sind Schätzungen von Wahrscheinlichkeiten und lassen keine konkreten Vorhersagen für eine Person zu. Fragen Sie Ihr Behandlungsteam, wenn Sie sich unsicher sind, ob sich die Ergebnisse auf Ihre Situation übertragen lassen.
>
> *i*

Alltagsaktivitäten

100 Punkte = volle Funktionsfähigkeit

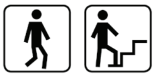

6, 24 Monate und 3 Jahre nach OP	**keine Unterschiede zwischen den Verfahren**<br>**Hinweise auf bessere Funktionsfähigkeit nach beiden Verfahren**	1	60

Schmerzen

keine Erkenntnisse aus Studien vorliegend

Lebensqualität

100 Punkte = vollstes Wohlbefinden

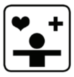

3, 6, 12 und 24 Monate nach OP	**keine Unterschiede zwischen den Verfahren**<br>**Hinweise auf höheres Wohlbefinden nach beiden Verfahren**	1	60

Sportliche Aktivität

keine Erkenntnisse aus Studien vorliegend

Komplikationen einer Total-Endoprothese mit Standard- und robotergestützter Navigation im Vergleich

In randomisiert-kontrollierten Studien, die den Nachweis erbringen können, ob eine Behandlung besser als eine andere ist (siehe Seite 10), zeigten sich keine Unterschiede hinsichtlich der Häufigkeit von Komplikationen.

Zusätzliche Daten aus sogenannten Registerstudien sind nicht verfügbar.

Eine ausführliche Übersicht zu den Komplikationen einer Total-Endoprothese finden Sie im Aufklärungsbogen auf den Seiten 14-20.

Was kann ich für die Entlassung vorbereiten? [1]

Gestaltung des häuslichen Umfeldes

Nach dem Eingriff können Sie ihr Bein zunächst nicht wie gewohnt belasten. Sie benötigen Hilfsmittel beim Gehen. Dies sind in der Regel entweder Gehhilfen oder ein Gehwagen. Es kann hilfreich sein, Vorkehrungen zu treffen und Tipps zu berücksichtigen.

Beseitigen Sie alle Stolperfallen wie lose Teppiche, nicht standfeste Möbel und legen Sie einen Stolperschutz über kleine Stufen. Positionieren Sie Möbel nach Möglichkeit so, dass sie ausreichend Platz haben, sich mit Gehhilfen problemlos zu bewegen. Decken Sie spitze Möbelkanten ab. Beachten Sie, dass alle losen Gegenstände oder leichte Möbel/Möbel auf Rollen eine Sturzgefahr darstellen können, wenn Sie sich daran festhalten. Prüfen Sie, ob Treppen rutschfest und eben sind und Treppengeländer stabil sind.

Für die erste Zeit zuhause ist es wichtig, sich im Vorfeld Unterstützung zu organisieren. Sie können nicht alle Haushaltstätigkeiten allein ausführen. Einkäufe sollten von einer Hilfsperson erledigt werden. Neben Familie, Freunden und Verwandten kann Sie eine Haushaltshilfe unterstützen. Fragen Sie bei Bedarf bei Ihrer Krankenkasse nach einer Kostenübernahme oder wenden Sie sich an den Sozialdienst.

Vor dem Krankenhausaufenthalt können Sie Lebensmittel und andere wichtige Dinge des täglichen Bedarfs auf Vorrat einkaufen. Ordnen Sie in der Küche alle Kochutensilien und Geschirr so an, dass sie im Sitzen erreichbar sind. Stellen Sie sich einen Stuhl vor den Herd, sodass Sie gegebenenfalls sitzend kochen können.

Sorgen Sie im Badezimmer für ausreichend Platz, um sich sicher mit Ihrer Gehhilfe bewegen zu können. Stellen Sie sich gegebenenfalls eine Sitzmöglichkeit vor das Waschbecken. Falls Sie eine tiefliegende Toilette haben, können Sie sich eine Sitzerhöhung beschaffen. In der Dusche können Sie einen stabilen Hocker platzieren. Organisieren Sie sich gegebenenfalls Anti-Rutschmatten und legen Sie das Bad und die Dusche damit aus.

Haustiere

Wenn Sie Haustiere besitzen, können Sie sich bereits im Vorfeld um eine Person kümmern, die Sie unterstützt. Diese kann zum Beispiel Spaziergänge, Tierarztbesuche oder die Pflege des Tieres übernehmen.

Hinweise für die Zeit nach der Operation [1]

Vor der Rehabilitation

Bevor Sie das Krankenhaus verlassen, bekommen Sie vom Behandlungsteam Übungen, die Sie im Anschluss an Ihren Krankenhausaufenthalt selbstständig durchführen können, wenn Sie ihre Rehabilitation nicht nahtlos antreten (können). Sie können mit diesen Übungen versuchen, den Heilungsprozess aktiv zu gestalten und die Übungen selbstständig regelmäßig ausführen.

Arbeitsfähigkeit

Wann man mit einem künstlichen Kniegelenk wieder arbeitsfähig ist, ist sehr unterschiedlich. Es ist abhängig vom Zustand vor der Operation, dem individuellen Heilungsprozess und der Art des Berufes. Sie sollten von acht bis über 12 Wochen ausgehen, je nachdem ob Sie einer sitzenden Tätigkeit oder einer körperlich anstrengenden Tätigkeit nachgehen. Manche Berufe sind mit einem künstlichen Kniegelenk möglicherweise nicht geeignet. Hierzu zählen beispielsweise Tätigkeiten, die mit häufigem Knien oder Hocken verbunden sind. Sprechen Sie mit Ihrer Ärztin oder Ihrem Arzt über die voraussichtliche Dauer Ihrer Arbeitsunfähigkeit. Bei Fragen zu Ihrer individuellen beruflichen Situation können Sie sich ebenfalls an das Behandlungsteam wenden.

Autofahren

Wann Sie wieder selbst Autofahren können, ist von verschiedenen Faktoren abhängig. Zum Beispiel davon, ob es ein Automatik- oder Schaltgetriebe ist und an welchem Bein Sie operiert wurden. In jedem Fall müssen Sie in der Lage sein, das Fahrzeug sicher zu führen. Sie müssen unter anderem genügend Kraft und Reaktionsschnelligkeit aufbringen können, um sicher zu bremsen. Dazu müssen Sie auch das Knie entsprechend strecken können. Je nach Heilungsverlauf sollten sie mit 12 Wochen rechnen, wenn Sie das betroffene Bein zum Autofahren benötigen. Sprechen Sie für individuelle Informationen mit Ihrem Behandlungsteam.

Sportliche Aktivitäten

Viele Menschen können nach der Operation wieder aktiver sein, da sie nicht mehr durch die starken Knieschmerzen eingeschränkt sind. Auch mit künstlichem Kniegelenk sind viele Sportarten möglich. Besonders eigenen sich zum Beispiel Radfahren, Schwimmen und Nordic-Walking. Gelenkbelastende Sportarten können dazu führen, dass die Prothese eher verschleißt und möglicherweise früher ausgetauscht werden muss. Dazu gehören vor allem Sportarten mit wiederholten Stoßbelastungen wie Fußball oder Joggen. Daneben kommt es auch darauf an, ob Sie eine Sportart neu erlernen möchten oder diese gut beherrschen. Fragen Sie im Zweifel Ihr Behandlungsteam.

Sexualität

Wenn Sie nach der Operation sexuell aktiv werden möchten, wählen Sie am besten eine Stellung, in der Sie keine Schmerzen im operierten Knie haben. Außerdem können Sie ihre Partnerin/ihren Partner bitten, den aktiveren Teil zu übernehmen. Sollten Sie Fragen haben, wenden Sie sich an Ihr Behandlungsteam.

Wo finde ich weitere Informationen?

Weitere Informationen zur Kniearthrose und deren Behandlungsmöglichkeiten finden Sie auf der Internetseite des **Instituts für Qualität und Wirtschaftlichkeit im Gesundheitswesen** (IQWiG).

www.gesundheitsinformation.de/kniearthrose-gonarthrose.html

Informationen und Möglichkeiten zur Selbsthilfe finden Sie außerdem auf folgenden Internetseiten:

Deutsche Arthrose Stiftung

www.deutsche-arthrose-stiftung.de

Deutsche Arthrose-Hilfe e.V.

www.arthrose.de

Wer hat die Informationsbroschüre mit welchen Quellen, wie erstellt?

Auf dieser Webseite sind alle verwendeten Quellen sowie alle beteiligten Personen aufgelistet.

https://evab-pilot.leitlinie-gesundheitsinformation.de

Ihre Fragen/Notizen

Anästhesieverfahren für einen Gelenkersatz an den unteren Gliedmaßen

Aufklärungsbogen für die Vollnarkose und die Spinalanästhesie

Klinikdruck/Stempel

Patientendaten

Sehr geehrte Patientin, sehr geehrter Patient,

Sie erwägen, sich einem operativen Eingriff zu unterziehen, bei dem eine Anästhesie erforderlich ist. Dieser Aufklärungsbogen hat das Ziel, Sie bei einer informierten Entscheidung zum Anästhesieverfahren zu unterstützen und auf das Aufklärungsgespräch vorzubereiten. Bitte lesen Sie den Aufklärungsbogen aufmerksam durch und bringen diesen zum Aufklärungsgespräch mit.

An wen richtet sich der Aufklärungsbogen?

Die folgenden Informationen richten sich an Patientinnen und Patienten, die sich einem Gelenkersatz am Knie oder an der Hüfte unterziehen. Die folgenden Informationen gelten nicht für Patientinnen und Patienten, die sich einem notfallmäßigen Eingriff unterziehen müssen.

Inhalt

Welche Anästhesieverfahren sind möglich? 2
Was sollte ich zu den Informationen in diesem Aufklärungsbogen wissen? 4
Welche Komplikationen und Risiken können auftreten? 5-21
Welche zusätzlichen Maßnahmen sind während der Anästhesie möglich? 22
Was gibt es vor der Anästhesie zu beachten? 22
Was geschieht nach der Operation? 23
Was gibt es nach der Anästhesie zu beachten? 23
Wer hat den Aufklärungsbogen mit welchen Quellen, wie erstellt? 23
Wichtige Fragen zu Ihrem Gesundheitszustand 24-28
Anmerkungen zum Aufklärungsgespräch 29
Einwilligung 30

Welche Anästhesieverfahren sind möglich?

Damit Sie während der Operation keine Schmerzen haben, gibt es zwei Möglichkeiten diese zu betäuben. Es besteht die Auswahl zwischen einer Vollnarkose oder einer Spinalanästhesie. Abgesehen von Ihrer persönlichen Vorliebe kann es sein, dass ein Verfahren für Sie individuell besser geeignet ist als das andere. Zum Beispiel, weil Sie bestimmte Nebenerkrankungen haben und diese mit einem der Anästhesieverfahren weniger gut vereinbar sind. Gemeinsam mit dem Behandlungsteam können Sie Ihre Wünsche und Sorgen besprechen und das für Sie passende Verfahren auswählen. Unabhängig von dem gewählten Verfahren erhalten Sie vor der Operation einen Venenzugang (Venenverweilkanüle), über den während der Operation Medikamente gegeben werden können.

Während der gesamten Operation werden Sie von einer Ärztin oder einem Arzt begleitet und überwacht. Die Risiken einer Vollnarkose und Spinalanästhesie im Vergleich finden Sie auf den Seiten 7-12.

Was ist eine Vollnarkose? [1]

Bei einer Vollnarkose bekommen Sie über den Venenzugang oder die Atemluft (Inhalationsnarkose) zunächst ein Narkosemittel verabreicht, das sehr schnell wirkt. Dann werden für die Dauer der Operation Medikamente in den Körper geleitet, die Sie in einem sehr tiefen Schlaf halten. Für die Dauer der Narkose ist sowohl ihr Bewusstsein als auch ihr Schmerzempfinden ausgeschaltet. Ist die Operation vorüber, wird die Gabe der Narkosemedikamente gestoppt und Sie wachen langsam wieder auf.

Während der Vollnarkose ist es nötig, Sie zu beatmen. Nachdem Sie eingeschlafen sind, wird die Ärztin oder der Arzt bei Ihnen entweder eine sogenannte Kehlkopfmaske einsetzen oder eine Intubation durchführen. Mit Ihrem Behandlungsteam können Sie besprechen, welches Verfahren für Sie am besten geeignet ist. Risiken einer Kehlkopfmaske und einer Intubation im Vergleich finden Sie auf den Seiten 13-17.

Kehlkopfmaske (Larynxmaske)

Eine Kehlkopfmaske besteht aus einem ovalen Endstück, dessen Rand mit Luft befüllt werden kann und einem Beatmungsschlauch. Eine Ärztin oder ein Arzt führt die Maske über den Mund in den Rachenraum bis auf Höhe des Kehlkopfes ein.

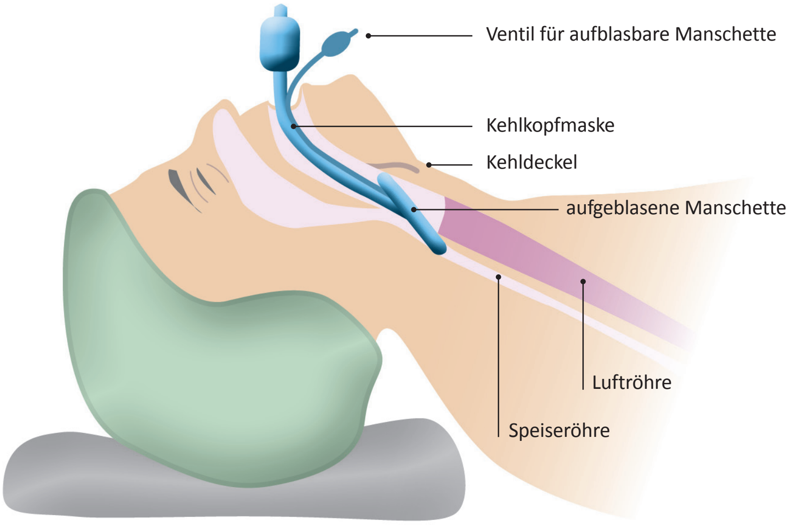

Abbildung 1: Beatmung mit Kehlkopfmaske

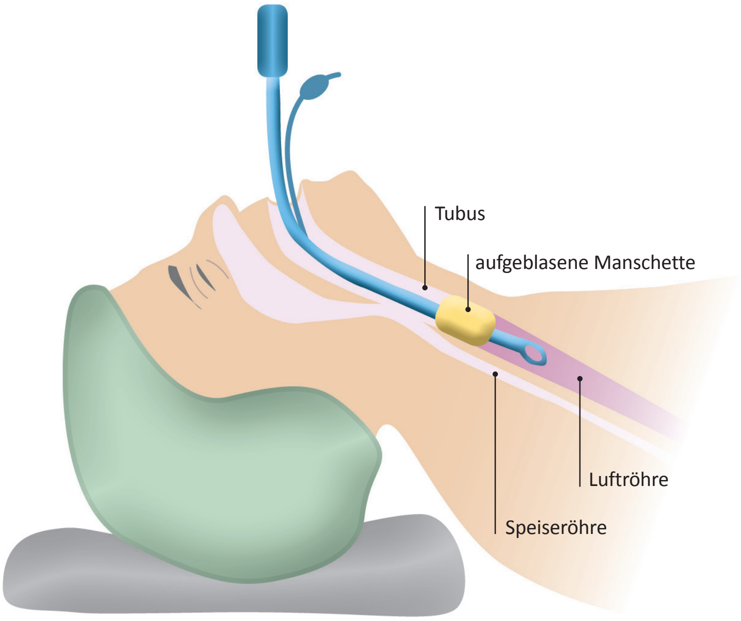

Abbildung 2: Beatmung mit Intubation

Endotracheale Intubation

Bei der Intubation führt die Ärztin oder der Arzt mithilfe eines Instruments (Laryngoskop) einen Schlauch in die Luftröhre ein. Am oberen Ende kann der Beatmungsschlauch angeschlossen werden. Am unteren Ende des Schlauches befindet sich ein kleiner Ballon, der aufgeblasen werden kann.

Was ist eine Spinalanästhesie? [1]

Bei einer Spinalanästhesie wird ein örtliches Betäubungsmittel in den Spinalraum im unteren Rückenbereich eingebracht. Die Betäubung setzt erst langsam ein und beginnt mit einem zunehmenden Kribbeln, bis Sie ihre Beine nicht mehr bewegen können und keinen Schmerz mehr verspüren. Dieser Zustand kann mehrere Stunden anhalten. Zuerst wird eine etwas dickere, spitze Nadel bis kurz vor die harte Haut des Rückenmarks eingeführt. Durch diese hindurch wird eine dünnere Nadel, die vorne stumpf ist, geschoben. Diese durchdringt die harte Rückenmarkshaut und das Betäubungsmittel wird eingebracht.

Die Haut an der Einstichstelle am Rücken kann vorher örtlich betäubt werden. Bevor die Operation beginnt, überprüfen die Ärztinnen und Ärzte noch, ob Sie am Bein tatsächlich nichts spüren.

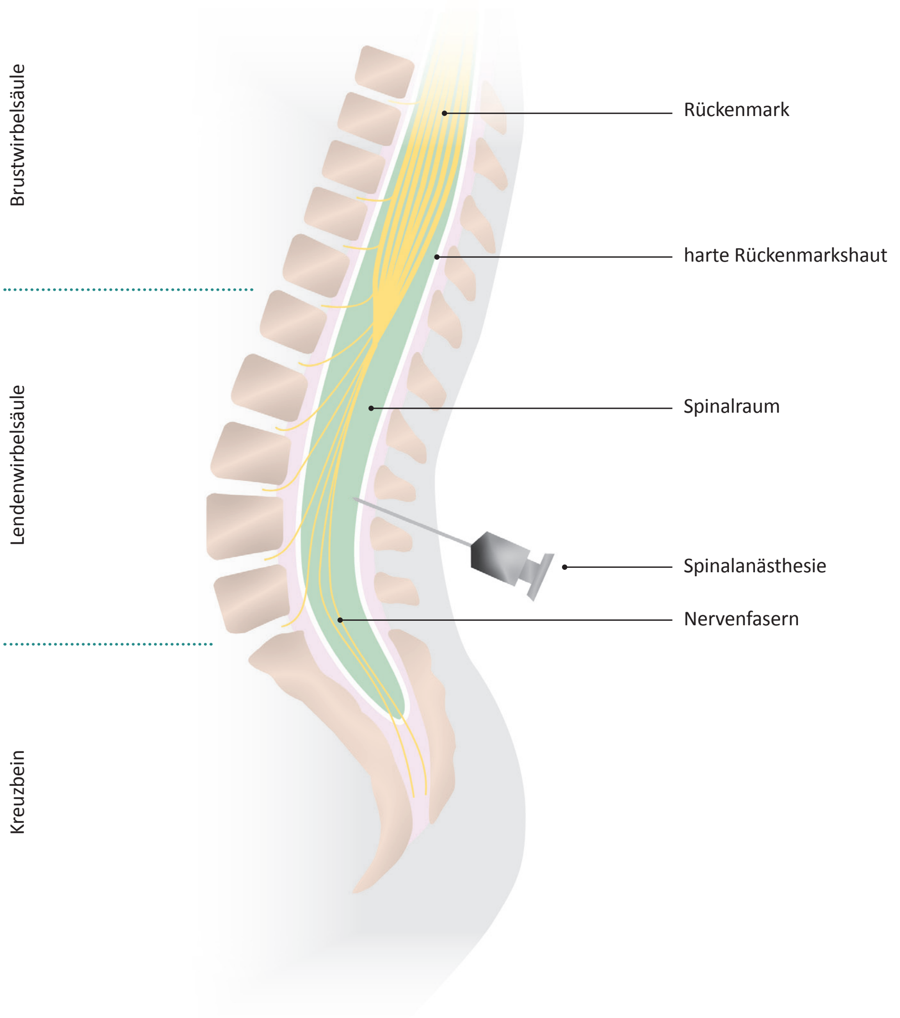

Abbildung 3: Wirbelsäule mit Spinalanästhesie

Wird bei der Spinalanästhesie das Rückenmark berührt?

Nein. Das Rückenmark endet im Bereich der Lendenwirbelsäule. Dort sind nur noch einzelne Nervenfasern, die aus dem Rückenmark hervorgehen. Diese versorgen den unteren Bereich des Körpers. Das Betäubungsmittel wird unterhalb des Rückenmarks auf Höhe der einzelnen Nervenfasern eingebracht.

Bin ich bei der Spinalanästhesie die ganze Zeit wach? Kann ich ein Beruhigungsmittel erhalten?

Nachdem nur der untere Teil des Körpers von der Betäubung betroffen ist, sind Sie während der Operation wach und ansprechbar. Wenn Sie dies nicht möchten, ist eine Sedierung möglich. Bei einer Sedierung können verschiedene Beruhigungsmittel verabreicht werden, die angstlösend, beruhigend und/oder schlaffördernd wirken. Je nach Dosierung und persönlichen Voraussetzungen kann es auch zu einem tiefen Schlaf kommen. Die Risiken einer Sedierung im Vergleich zum Verzicht auf die Gabe von Beruhigungsmitteln finden Sie auf den Seiten 17-19.

Was sollte ich zu den Informationen in diesem Aufklärungsbogen wissen?

Diese Broschüre wurde mit den Methoden der evidenzbasierten Medizin entwickelt. Hierbei werden die derzeit besten verfügbaren Studien als Informationsquelle genutzt.

Häufigkeiten zu den Komplikationen wurden aus verschiedenen Arten von Studien ermittelt. Es ist gut, den Unterschied zwischen diesen Studienarten zu kennen, weil ihre Aussagekraft unterschiedlich ist.

Randomisiert-kontrollierte Studien (RCTs)

Ein Teil der Zahlen stammt aus RCTs. Durch die methodischen Standards dieser Art von Studien kann ein direkter Nachweis erbracht werden, bei welchem Anästhesieverfahren weniger Komplikationen auftreten. Weitere Informationen dazu, wie randomisiert-kontrollierte Studien genau ablaufen, können Sie auf Seite 3 im Aufklärungsbogen „Künstliches Kniegelenk" finden.

Registerstudien

Ein anderer Teil der Zahlen stammt aus sogenannten Registerstudien. In manchen Ländern werden große Datenbanken zu den eingesetzten Anästhesieverfahren angelegt und es wird erfasst, wie oft bestimmte Komplikationen bei den verschiedenen Verfahren jeweils aufgetreten sind. Die Ergebnisse aus Registerstudien beruhen auf den Daten von sehr vielen Personen. Sie erfüllen aber nicht die methodischen Standards von randomisiert-kontrollierten Studien. Es kann nicht sicher gesagt werden, ob ein Unterschied in den Ergebnissen tatsächlich auf ein bestimmtes Anästhesieverfahren zurückzuführen ist oder unterschiedliche Ausgangsvoraussetzungen dafür verantwortlich sind.

Welche Sicherheiten und Unsicherheiten sind mit den Zahlen verbunden?

Zahlen vermitteln den Eindruck von Genauigkeit. Tatsächlich sind sie mit vielen Unsicherheiten verbunden. Zahlen als Ergebnisse wissenschaftlicher Studien sind nur Schätzwerte. Wie genau die Zahlen geschätzt werden, hängt zum Beispiel davon ab, wie groß eine Studie ist. Außerdem handelt es sich um Wahrscheinlichkeiten. Für die einzelne Person lassen sich keine sicheren Vorhersagen treffen.

Sind die Zahlen im Aufklärungsbogen vollständig auf mich übertragbar?

An den Studien, die für die Erstellung des Aufklärungsbogens verwendet wurden, nahmen auch Menschen teil, die sich hinsichtlich der Erkrankung und möglicher Nebenerkrankungen von der Zielgruppe dieser Information unterscheiden. Es ist daher möglich, dass sich die Ergebnisse nicht vollständig auf Sie übertragen lassen. Wenn Sie wissen wollen, ob sich die Studienergebnisse auf Ihre Situation übertragen lassen, sprechen Sie bitte mit Ihrer Ärztin oder Ihrem Arzt darüber.

Ihre Fragen/Notizen

Welche Komplikationen und Risiken können auftreten?

Für die Komplikationen wurden mehrere Studienergebnisse zusammengefasst. Der Verlauf einer Komplikation kann nicht konkret vorhergesagt werden. Komplikationen können verschiedene Schweregrade haben. Folgen können vorübergehend oder bleibend sein und zu keinen bis stärksten Einschränkungen, einschließlich Tod, führen. Die Folge von Komplikationen können weitere Behandlungen und ein verlängerter oder erneuter Krankenhausaufenthalt sein. Das Auftreten von Komplikationen kann auch von individuellen Neben- und Vorerkrankungen und anderen individuellen Besonderheiten beeinflusst werden.

Es können im Rahmen der Anästhesie auch Medikamente eingesetzt werden, die keine offizielle Zulassung für diesen Zweck haben (Off-Label-Use). Das Behandlungsteam wird Sie in diesem Fall über die jeweiligen speziellen Komplikationen aufklären. Es können hierbei auch bislang unbekannte Risiken auftreten. Der Hersteller des Medikamentes übernimmt dann möglicherweise keine Haftung.

Während der Operation werden Sie durchgängig überwacht. Auf Komplikationen kann so zeitnah reagiert werden.

Wie können die Ergebnisse gelesen werden?

Die folgenden Abbildungen helfen Ihnen, die Häufigkeit von Komplikationen zu verstehen. Zwischen den verschiedenen Verfahren (z.B. zwischen Vollnarkose und Spinalanästhesie) kann es hinsichtlich einer Komplikation Unterschiede gegeben haben. Es können sich aber genauso keine Unterschiede gezeigt haben.

Die folgende Abbildung gibt Ihnen eine Anleitung, wie Sie die Abbildungen lesen können, wenn keine Unterschiede zwischen Verfahren berichtet wurden.

Komplikation	Häufigkeit
**Harnverhalt**<br>Es kann zu **Harnverhalt** infolge der Operation kommen. Wenn die Blase voll ist und nicht selbstständig entleert werden kann, muss der Urin durch einen Blasenkatheter abgeleitet werden.	**Keine Unterschiede zwischen den Verfahren**

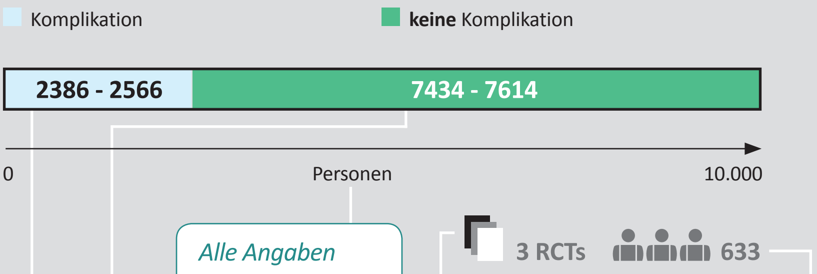

Alle Angaben beziehen sich auf 10.000 Personen.

Anzahl und Typ der Studien, die in die Darstellung der Ergebnisse eingeflossen sind.

Anzahl der Teilnehmenden, die in die Darstellung der Ergebnisse eingeflossen sind.

Minimaler und maximaler Anteil an Personen, die nach der Anästhesie Harnverhalt hatten.

„Der Anteil an Personen mit Harnverhalt liegt zwischen 2800 und 2970 von 10.000 Personen (28% - 29,7%).“

*Minimaler und maximaler Anteil an Personen, die nach der Anästhesie **keinen** Harnverhalt hatten.*

*„Der Anteil an Personen mit **keinem** Harnverhalt liegt zwischen 7030 und 7200 von 10.000 Personen (70,3% - 72%).“*

Wie können die Ergebnisse gelesen werden?

Die folgende Abbildung gibt Ihnen eine Anleitung, wie Sie die Abbildungen lesen können, wenn Unterschiede zwischen Verfahren berichtet wurden.

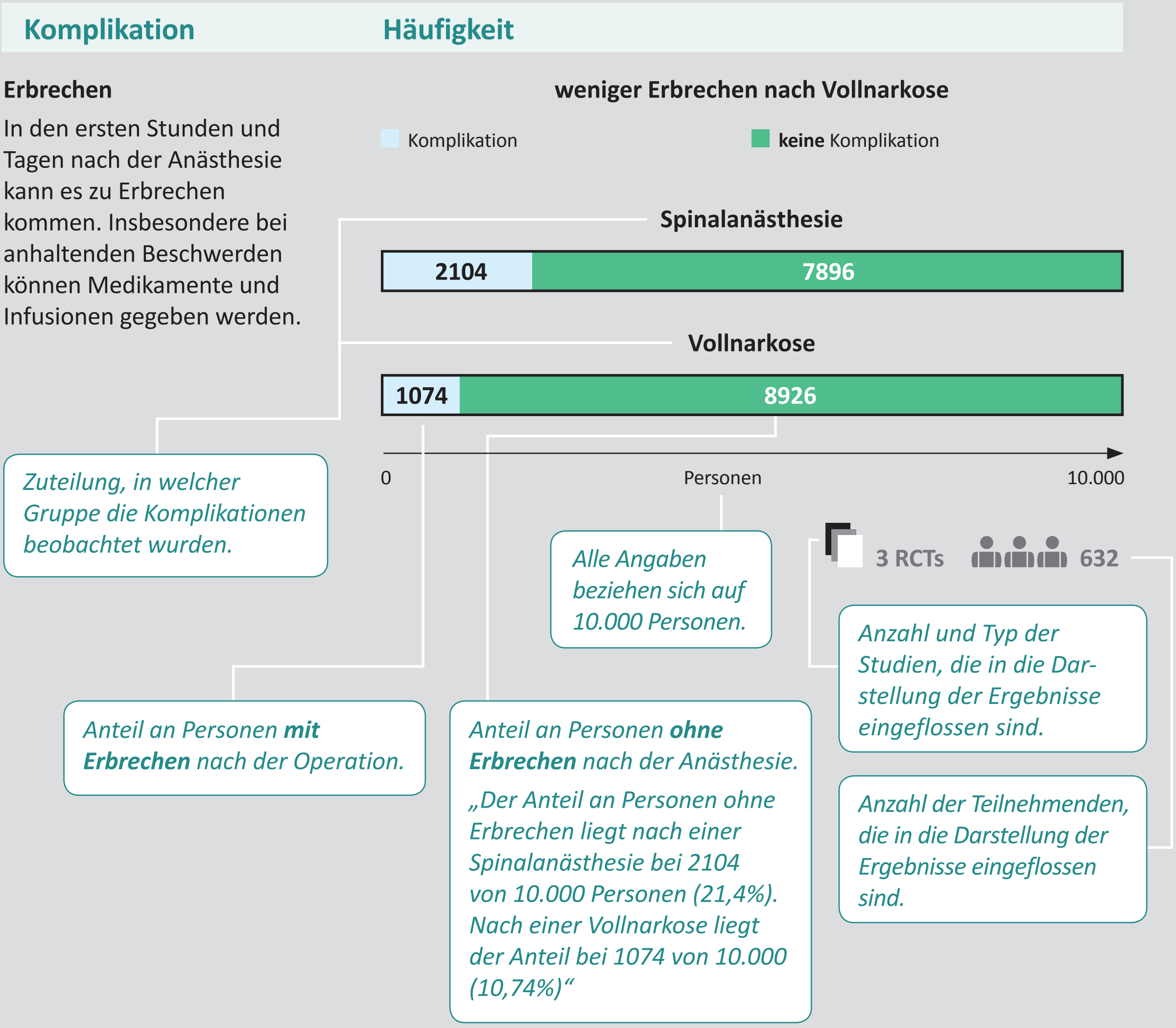

i Zahlen sind Schätzungen von Wahrscheinlichkeiten und lassen keine konkreten Vorhersagen für eine Person zu. Fragen Sie Ihr Behandlungsteam, wenn Sie sich unsicher sind, ob sich die Ergebnisse auf Ihre Situation übertragen lassen.

Vollnarkose und Spinalanästhesie: Welche Komplikationen können im Allgemeinen auftreten? [2]

Für die Angaben zu den Häufigkeiten der Komplikationen wurden mehrere Studien zusammengefasst. Die Teilnehmenden in den Studien wurden in der Regel bis 30 Tage nach der Operation beobachtet. Wann genau die Komplikation jeweils aufgetreten ist, lässt sich nicht genau sagen. Diese können während, kurz nach oder erst später nach der Operation auftreten. Manchen Komplikationen (z.B. Übelkeit, Erbrechen, Delir) treten frühzeitig nach der OP auf und wurden bis zu 4 Tage nach der OP erfasst. Andere Komplikationen (z.B. Lungenentzündung, Niereninsuffizienz) können auch erst später nach der Operation auftreten und wurden bis zu 30 Tage nach der OP erfasst.

Risiken und Komplikationen, die das Gehirn und das Nervensystem betreffen

Komplikation | **Häufigkeit** — Vollnarkose verglichen mit Spinalanästhesie

Ein **Delir (Durchgangs-Syndrom)** ist ein akut auftretender Zustand von Verwirrung. Es kann zu Unruhe, Problemen mit der Orientierung, fehlender Konzentration und Erinnerungsschwäche kommen. Auch kann nach der Operation die geistige Leitungsfähigkeit kurzfristig oder dauerhaft beeinträchtigt sein. Ältere Personen und Personen mit Vorerkrankungen (z.B. Schlaganfall) sind davon eher betroffen.

Keine Unterschiede zwischen den Verfahren

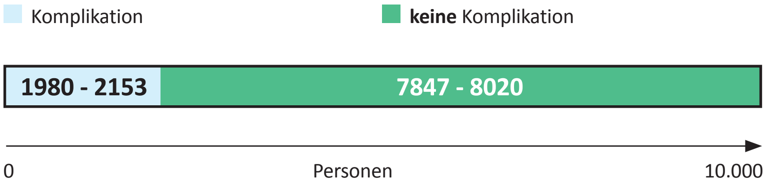

3 RCTs 545

Ein **Schlaganfall** kann lebensbedrohlich sein und infolge von geschädigten Hirnarealen zu vorübergehenden und/oder anhaltenden Funktionseinschränkungen führen.

Hinweise auf geringeren Anteil an Personen mit Schlaganfall nach Spinalanästhesie

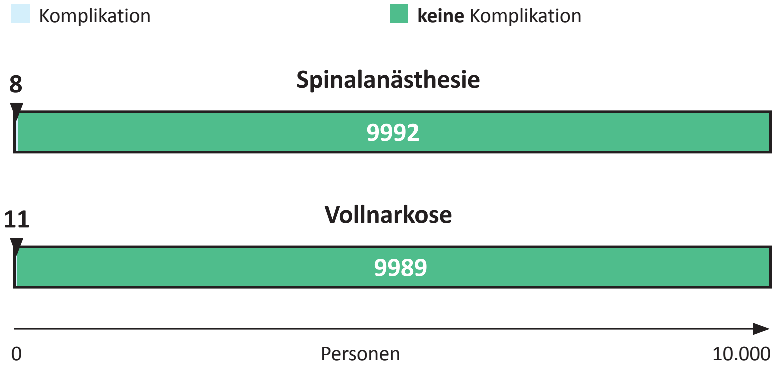

6 Register 363.521

Komplikationen, die das Herz- Kreislaufsystem betreffen

Komplikation	Häufigkeit	Vollnarkose verglichen mit Spinalanästhesie

Ein **Herzinfarkt** kann lebensbedrohlich sein und erfordert eine sofortige Behandlung. Er kann während oder nach der Operation auftreten. Je nach Schwere können anhaltende Einschränkungen und weitere Behandlungen die Folge sein.

keine Hinweise auf Unterschiede zwischen den Verfahren

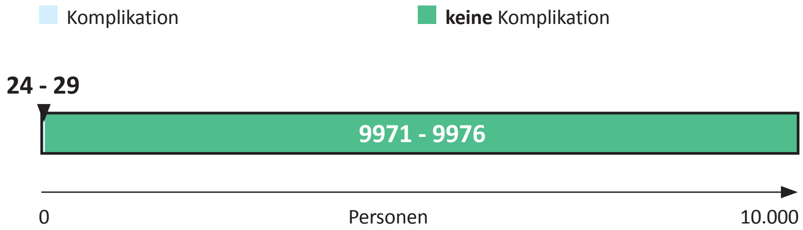

5 Register 349.193

Ein **Herzstillstand** ist lebensbedrohlich und erfordert eine sofortige Behandlung. Es kann während oder nach der Operation auftreten. Je nach Dauer des Herzstillstandes können anhaltende Einschränkungen die Folge sein.

keine Hinweise auf Unterschiede zwischen den Verfahren

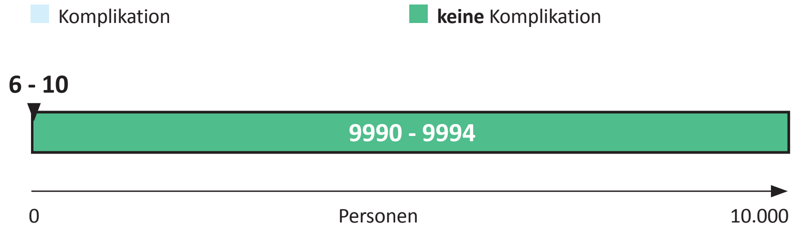

3 Register 329.031

Störungen des Herzrhythmus können zu weiteren Erkrankungen führen. Je nach Schwere und Dauer der Störung können weitere Behandlungen notwendig werden.

Diese Komplikation wurde nur in 1 RCT betrachtet.
Es kann daher über die Häufigkeit keine Angabe gemacht werden.

1 RCT 391

Komplikationen, die das Atmungssystem betreffen

Komplikation	Häufigkeit	Vollnarkose verglichen mit Spinalanästhesie

Eine **Lungenentzündung (Pneumonie)** ist eine Infektion, die durch Viren, Bakterien, oder eine Aspiration (Einatmung von Fremdkörpern oder Flüssigkeiten) entstehen kann. Je nach Schwere kann eine Behandlung notwendig werden (z.B. Antibiotika, Entfernen von Fremdkörpern/ Flüssigkeiten).

Hinweise auf geringeren Anteil an Personen mit Lungenentzündung nach Spinalanästhesie

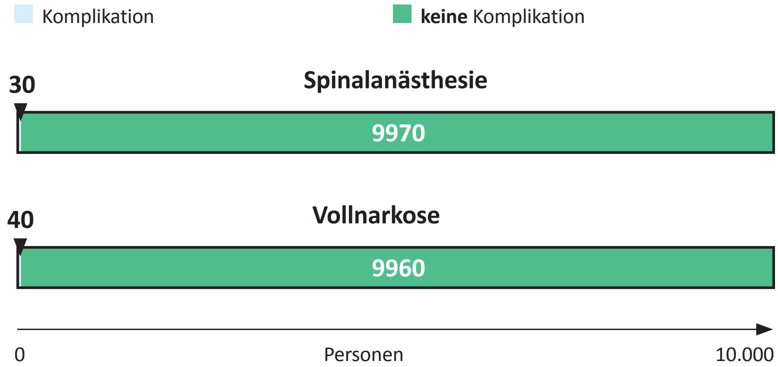

5 Register 349.193

Komplikation | **Häufigkeit** | Vollnarkose verglichen mit Spinalanästhesie

Eine **ungeplante Intubation** kann notwendig werden, wenn eine Spinalanästhesie nicht erfolgreich ist und eine Allgemeinanästhesie eingesetzt werden muss, die Beatmung über die Kehlkopfmaske bei der Allgemeinanästhesie nicht ausreichend ist oder das Einsetzen der Kehlkopfmaske fehlschlägt.

keine Hinweise auf Unterschiede zwischen den Verfahren

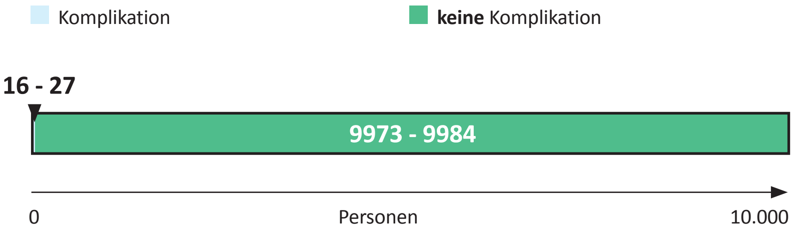

6 Register 359.643

Komplikationen, die den Magen-Darm-Trakt betreffen

Komplikation | **Häufigkeit** | Vollnarkose verglichen mit Spinalanästhesie

Erbrechen kann in den ersten Stunden und Tagen nach der Anästhesie auftreten. Insbesondere bei anhaltenden Beschwerden können Medikamente und Infusionen gegeben werden. Je nach Schwere und Dauer kann es zu Schäden an der Speiseröhre, des Magens oder der Bauchwand kommen. Dies kann eine weitere Behandlung notwendig machen.

weniger Erbrechen nach Vollnarkose

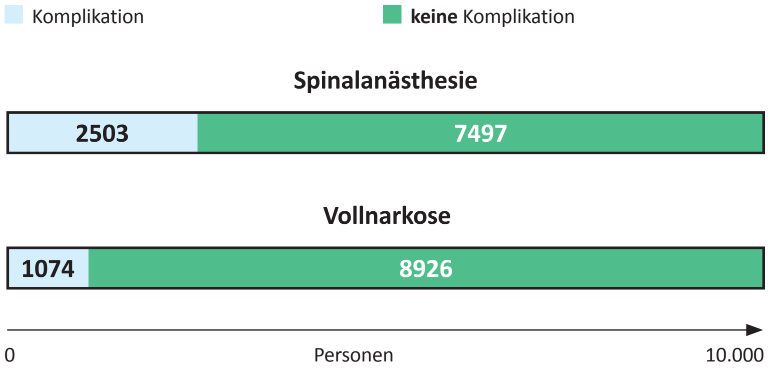

3 RCTs 632

Übelkeit kann in den ersten Stunden und Tagen nach der Anästhesie auftreten. Insbesondere bei anhaltenden Beschwerden können Medikamente gegeben werden.

keine Unterschiede zwischen den Verfahren

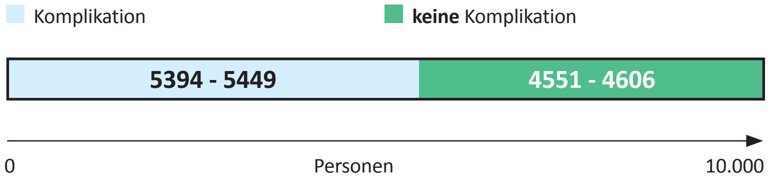

2 RCTs 425

Risiken und Komplikationen, die den Harntrakt betreffen

Komplikation	Häufigkeit	Vollnarkose verglichen mit Spinalanästhesie

Infektionen des Harntraktes können zum Beispiel durch die Anlage eines Blasenkatheters entstehen. Je nach Schwere der Infektion können weitere Behandlungen möglicherweise mit Antibiotika notwendig werden.

Hinweise auf weniger Harnwegsinfektionen nach Spinalanästhesie

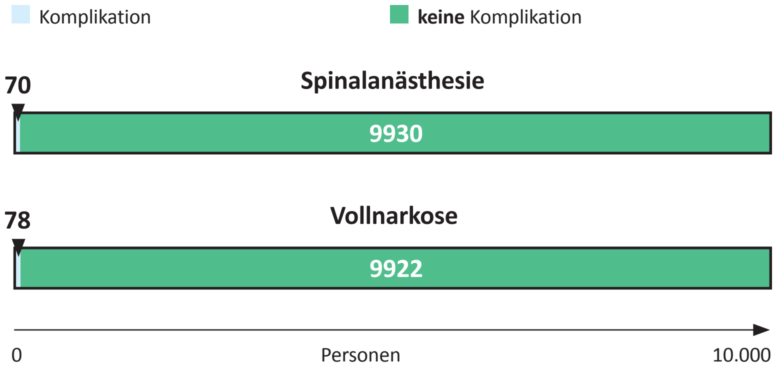

8 Register 1.146.936

Bei einer **Niereninsuffizienz** ist die Funktion der Nieren vorübergehend oder anhaltend (chronisch) eingeschränkt. Stoffe, die sonst über die Niere ausgeschieden werden, bleiben im Körper und können sich auf den gesamten Organismus auswirken. Je nach Schwere und Dauer der Einschränkung können verschiedene Behandlungen notwendig werden.

Hinweise auf geringeren Anteil an Personen mit Niereninsuffizienz nach Spinalanästhesie

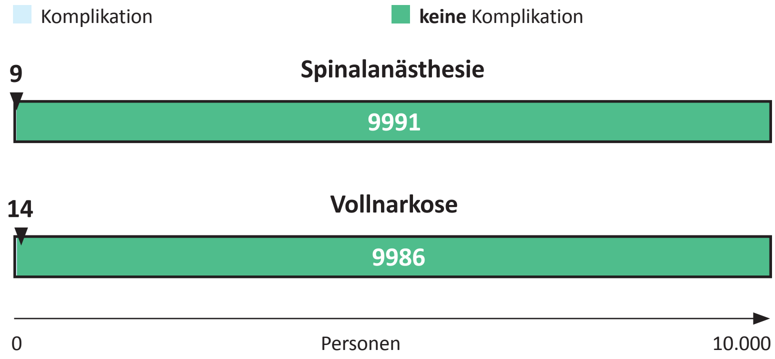

4 Register 331.062

Es kann zu **Harnverhalt** infolge der Operation kommen. Wenn die Blase voll ist und nicht selbstständig entleert werden kann, muss der Urin durch einen Blasenkatheter abgeleitet werden. Der Blasenkatheter kann mit weiteren Folgen verbunden sein (z. B. Verletzung der Harnröhre/-blase)

keine Unterschiede zwischen den Verfahren

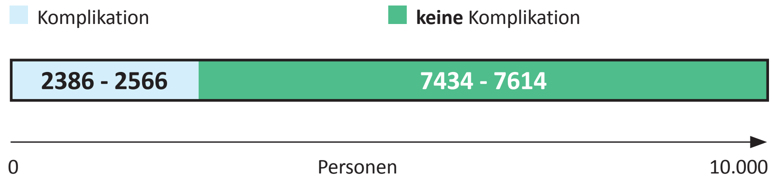

3 RCTs 633

Komplikation	Häufigkeit	Vollnarkose verglichen mit Spinalanästhesie

Ein plötzliches **Versagen der Nieren** erfordert sofortige Behandlung (z.B. Dialyse). Der Verlust der Organfunktion tritt innerhalb weniger Stunden auf und kann lebensgefährliche Folgen haben und anhaltende Einschränkungen mit sich bringen.

keine Hinweise auf Unterschiede zwischen den Verfahren

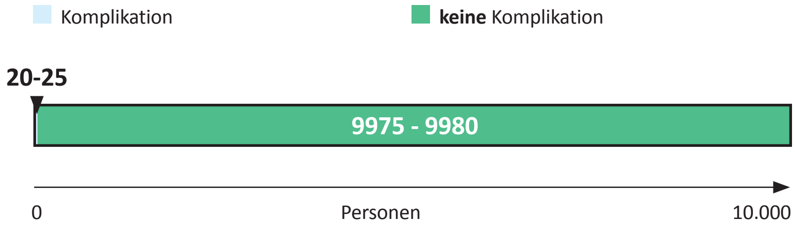

4 Register 340.554

Risiken und Komplikationen, die den gesamten Körper/Organismus betreffen

Komplikation	Häufigkeit	Vollnarkose verglichen mit Spinalanästhesie

Eine **Sepsis** kann infolge einer Infektion auftreten. Im Rahmen einer überschießenden Immunreaktion des Körpers kann sich die Infektion über die Blutbahn im gesamten Körper ausbreiten. Das kann lebensgefährliche Folgen haben und eine weitere Behandlung und einen Aufenthalt auf der Intensivstation notwendig machen.

Hinweise auf geringeren Anteil an Personen mit Sepsis nach Spinalanästhesie

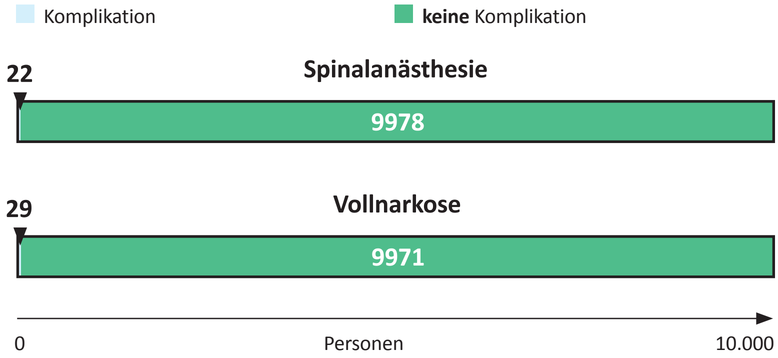

4 Register 331.062

Ein **septischer Schock** kann infolge einer Sepsis auftreten und durch einen sehr niedrigen Blutdruck die Sauerstoffversorgung lebenswichtiger Organe gefährden. Er macht eine sofortige Behandlung notwendig und kann lebensbedrohliche Folgen haben.

Hinweise auf geringeren Anteil an Personen mit septischem Schock nach Spinalanästhesie

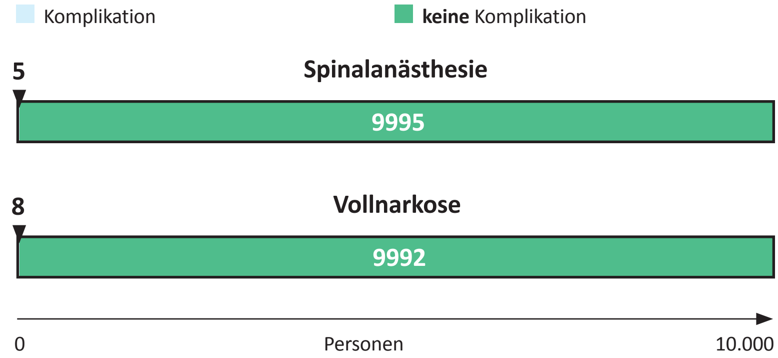

2 Register 308.095

Komplikation	Häufigkeit	Vollnarkose verglichen mit Spinalanästhesie
Es kann zum **Tod** infolge von Komplikationen während und nach der Anästhesie kommen. Es können aber auch andere Erkrankungen wie zum Beispiel Krebs oder Demenz für den Tod einer Person ursächlich sein.	**keine Hinweise auf Unterschiede zwischen den Verfahren** 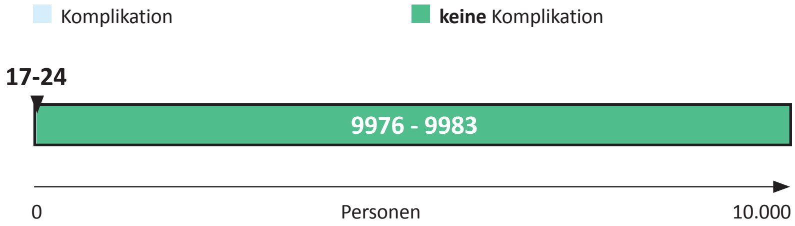 7 Register 375.881	
Ein **Koma** ist ein Zustand tiefer Bewusstlosigkeit. Es kann als Folge von Komplikationen auftreten oder als therapeutische Maßnahme eingeleitet werden.	Diese Komplikation wurde nur in 1 Register betrachtet. Es kann daher über die Häufigkeit keine Angabe gemacht werden. 1 Register 14.052	

Ihre Fragen/Notizen

Kehlkopfmaske und Intubation: Welche Komplikationen können auftreten? [3]

Während der Vollnarkose werden Sie beatmet. Dies kann entweder mit einer Kehlkopfmaske oder durch eine Intubation erfolgen. Die folgenden Komplikationen können während oder wenige Tage nach der Operation auftreten. Auf den Seiten 5 und 6 finden Sie eine Anleitung, wie die folgenden Balkendiagramme gelesen werden können.

Komplikationen, die den Mund- und Rachenraum betreffen

Komplikation	Häufigkeit	Kehlkopfmaske verglichen mit Intubation

Heiserkeit kann durch Verletzung oder Reizung der Schleimhäute durch das Beatmungsinstrument entstehen. Je nach Schwere der Reizung können die Beschwerden vorübergehend sein und über einige Stunden bis Tage anhalten. Sie können aber auch über einen längeren Zeitraum oder dauerhaft bestehen bleiben.

weniger Heiserkeit mit Kehlkopfmaske 2 Stunden nach OP

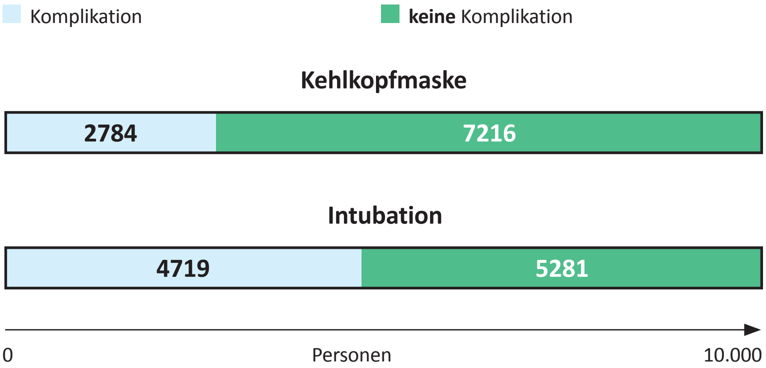

24 Stunden nach der Operation konnte kein Unterschied mehr zwischen den Verfahren ermittelt werden.

5 RCTs 458

Halsschmerzen können durch Verletzung oder Reizung der Schleimhäute durch das Beatmungsinstrument entstehen. Je nach Schwere der Reizung können die Beschwerden über einige Stunden bis Tage anhalten. Sie können aber auch über einen längeren Zeitraum oder dauerhaft bestehen bleiben.

weniger Halsschmerzen mit Kehlkopfmaske 24 Stunden nach OP

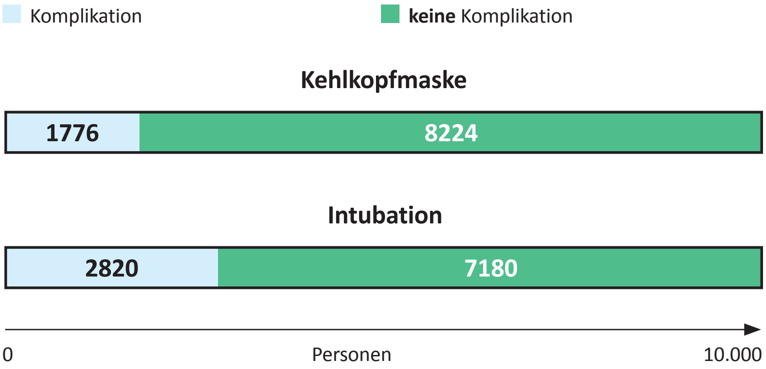

Auch 2 und 6 Stunden nach der Operation hatten die Studienteilnehmenden mit Kehlkopfmaske weniger Halsschmerzen.

14 RCTs 1.577

Komplikation	Häufigkeit	Kehlkopfmaske verglichen mit Intubation

Stimmstörungen können durch eine Verletzung oder Reizung im Bereich der Stimmbänder durch das Beatmungsinstrument entstehen. Es kann zum Beispiel zu einem veränderten Klang der Stimme oder zu einer verminderten Sprechlautstärke kommen. Je nach Schwere der Reizung können die Beschwerden vorübergehend sein und über einige Stunden bis Tage anhalten. Sie können aber auch über einen längeren Zeitraum oder dauerhaft bestehen bleiben.

weniger Stimmstörungen 2 Stunden nach OP mit Kehlkopfmaske

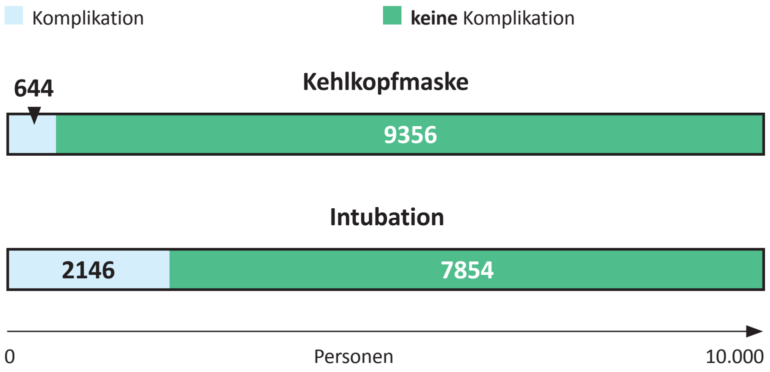

6 und 24 Stunden nach der Operation konnte kein Unterschied mehr zwischen den Verfahren ermittelt werden.

4 RCTs 470

Schluckstörungen können durch Verletzung oder Reizung der Schleimhäute durch das Beatmungsinstrument entstehen. Je nach Schwere der Reizung können die Schwierigkeiten beim Schlucken über einige Stunden bis Tage anhalten. Sie können aber auch über einen längeren Zeitraum oder dauerhaft bestehen bleiben.

keine Unterschiede zwischen den Verfahren 24 Stunden nach OP

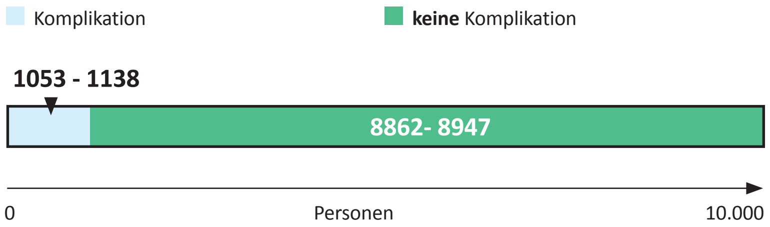

Auch 2 und 6 Stunden nach der Operation konnte kein Unterschied zwischen den Verfahren ermittelt werden.

7 RCTs 670

Verletzungen im Mundraum können während des Einführens oder Entfernens des Beatmungsinstrumentes entstehen. Zähne, Zahnersatz, Lippen, Zunge oder Gaumen können betroffen sein. Neben vorübergehenden Verletzungen können bleibende Missempfindungen zum Beispiel an der Zunge oder Zahnverlust entstehen. Je nach Schwere der Verletzung kann eine weitere Behandlung notwendig werden.

keine Unterschiede zwischen den Verfahren

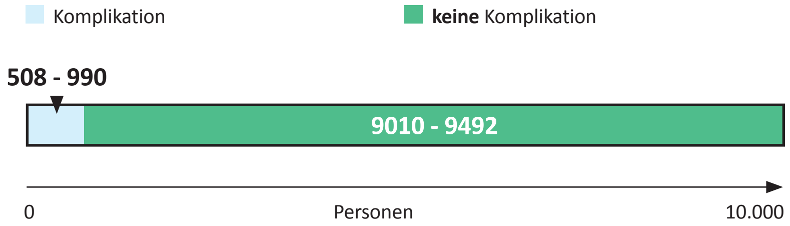

2 RCTs 147

Komplikation	Häufigkeit	Kehlkopfmaske verglichen mit Intubation

Eine **Verkrampfung der Kehlkopfmuskulatur (Laryngospasmus)** während der Operation kann verschiedene Ursachen haben. Je nach Schwere der Verkrampfung kann eine weitere Beatmung dadurch erschwert oder unmöglich werden und eine Behandlung mit Medikamenten erfordern.

keine Unterschiede zwischen den Verfahren

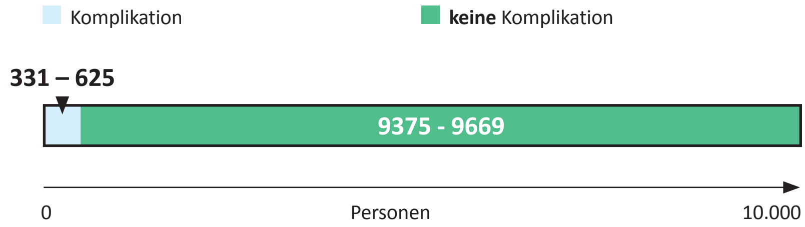

11 RCTs 790

Komplikationen, die das Atmungssystem betreffen

Komplikation	Häufigkeit	Kehlkopfmaske verglichen mit Intubation

Verletzungen der Atemwege/der Atemwegs-Schleimhaut können infolge des Einsetzens oder Entfernens des Beatmungsinstrumentes entstehen.
Je nach Schwere der Verletzung kann eine weitere Behandlung notwendig werden.

keine Unterschiede zwischen den Verfahren

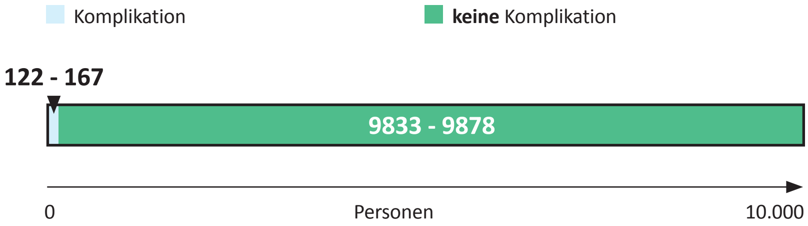

2 RCTs 164

Eine **Verkrampfung der Atemwegsmuskulatur (Bronchospasmus)** während der Operation kann verschiedene Ursachen haben. Je nach Schwere der Verkrampfung kann eine weitere Beatmung dadurch erschwert oder unmöglich werden und eine Behandlung mit Medikamenten erfordern.

keine Unterschiede zwischen den Verfahren

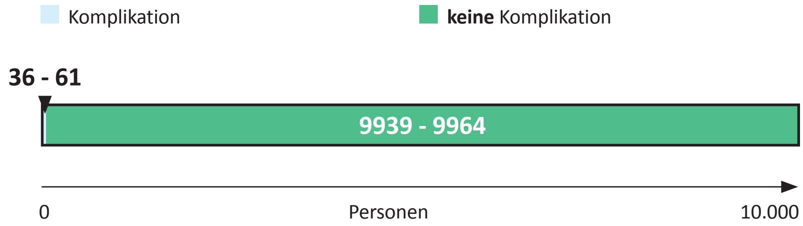

4 RCTs 339

Komplikation	Häufigkeit Kehlkopfmaske verglichen mit Intubation

Bei einer **Aspiration** gelangen Flüssigkeit, Essensreste oder Mageninhalt in die Atemwege. Dies kann zu einer Reizung der Atemwege und zu Infektionen führen. Je nach Schwere kann eine weitere Behandlung der Infektion oder eine Entfernung der Fremdkörper notwendig werden.

keine Unterschiede zwischen den Verfahren

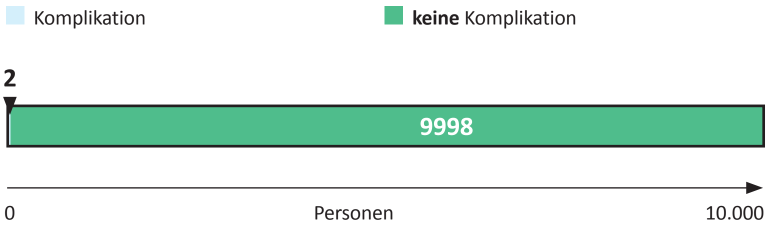

In der Studie wurden auch Kinder einbezogen. Die Zahlen sollten also mit Vorsicht interpretiert werden und ermöglichen nur einen Eindruck der Häufigkeit.

1 Register 65.712

Risiken und Komplikationen, die den gesamten Körper/Organismus betreffen

Komplikation	Häufigkeit Kehlkopfmaske verglichen mit Intubation

Unwillkürliches Zittern (Kältezittern) kann nach der Operation auftreten und unangenehm sein, je nach Schwere aber auch zu weiteren Komplikationen (z.B. Aufreißen von Nähten oder Störungen des Herz-Kreislaufsystems) führen. Je nach Dauer und Schwere des Zitterns können weitere Behandlungen nötig werden.

keine Unterschiede zwischen den Verfahren

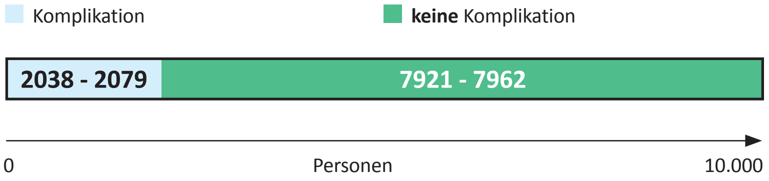

5 RCTs 345

Technische Risiken und Komplikationen

Komplikation	Häufigkeit	Kehlkopfmaske verglichen mit Intubation

Die **künstliche Beatmung kann fehlschlagen,** weil zum Beispiel das Beatmungsinstrument nicht eingeführt werden kann. Dies kann den Wechsel der Beatmungstechnik oder den Abbruch der Operation zur Folge haben.

weniger Fehlschläge bei Intubation

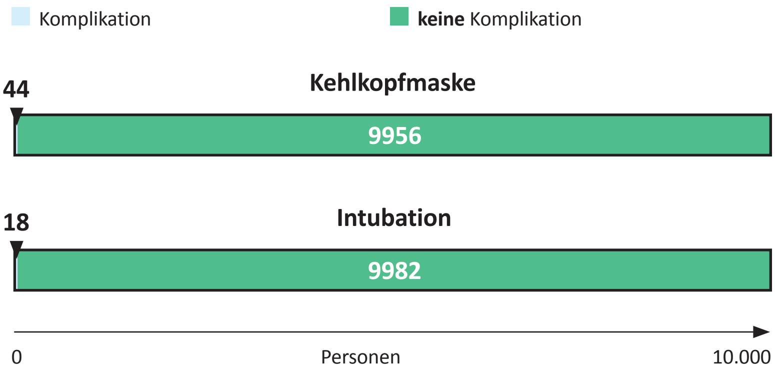

24 RCTs 2.326

Sedierung während der Spinalanästhesie: Welche Komplikationen können auftreten? [4]

Bei einer Spinalanästhesie können Sie entscheiden, ob Sie zusätzlich eine Sedierung haben möchten oder nicht. Bei der Sedierung werden Beruhigungsmittel verabreicht, die angstlösend, beruhigend und/oder schlaffördernd wirken. Die folgenden Komplikationen können während oder kurz nach der Operation auftreten. Auf den Seiten 5 und 6 finden Sie eine Anleitung, wie die folgenden Balkendiagramme gelesen werden können.

Komplikationen, die das Gehirn und das Nervensystem betreffen

Komplikation	Häufigkeit	Sedierung verglichen mit keiner Sedierung

Kopfschmerzen können aus verschiedenen Gründen nach der Operation (z.B. nach der Spinalanästhesie) auftreten. Je nach Schwere können die Schmerzen durch die Gabe von Medikamenten gelindert werden.

keine Unterschiede zwischen den Verfahren

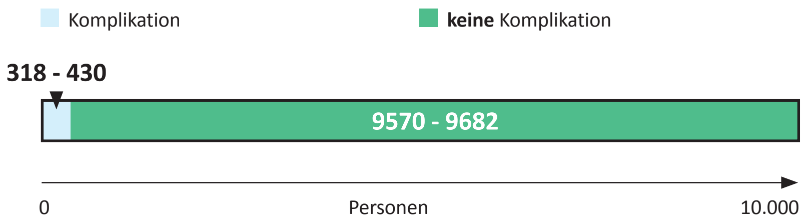

2 RCTs 208

Halluzinationen (Sinnestäuschungen) können nach oder während der Operation vorkommen und das Sehen, Riechen oder Hören betreffen.

Diese Komplikation wurde in 2 Studien betrachtet, ist jedoch nur in einer Studie überhaupt aufgetreten. Es kann daher über die Häufigkeit keine Angabe gemacht werden.

2 RCTs 221

Risiken und Komplikationen, die das Atmungssystem betreffen

Komplikation	Häufigkeit	Sedierung verglichen mit keiner Sedierung

Eine **rapide Verlangsamung der Atmung (Atemdepression)** zusammen mit einer reduzierten Sauerstoffsättigung während der Operation erfordert eine Behandlung. Dies kann durch die Gabe von Medikamenten oder die Sicherung der Atemwege durch eine Kehlkopfmaske oder Intubation erfolgen.

weniger Fälle von Atemdepression ohne Sedierung

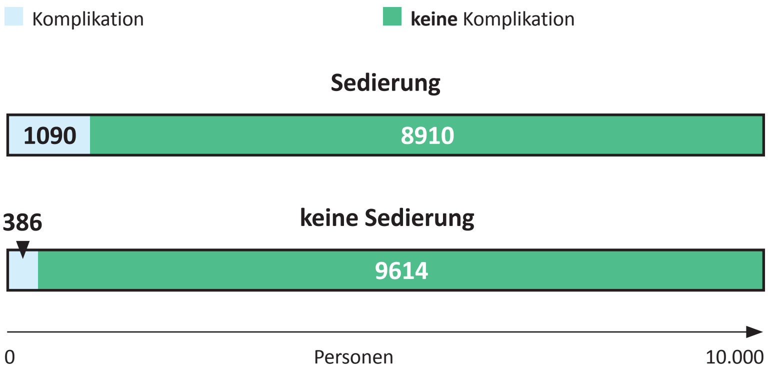

6 RCTs 554

Ein **Atemstillstand (Apnoe)** ist lebensbedrohlich und erfordert eine sofortige Behandlung. Er kann während oder nach der Operation auftreten. Je nach Dauer des Atemstillstandes können anhaltende Einschränkungen die Folge sein.

keine Unterschiede zwischen den Verfahren

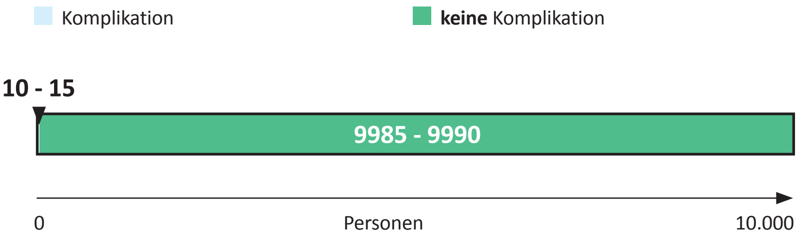

2 RCTs 133

Komplikationen, die das Herz- Kreislaufsystem betreffen

Komplikation	Häufigkeit	Sedierung verglichen mit keiner Sedierung

Ein **niedriger Blutdruck (Hypotonie)** während der Operation kann die ausreichende Versorgung der Organe mit Sauerstoff gefährden und eine Behandlung mit Medikamenten wird notwendig.

keine Unterschiede zwischen den Verfahren

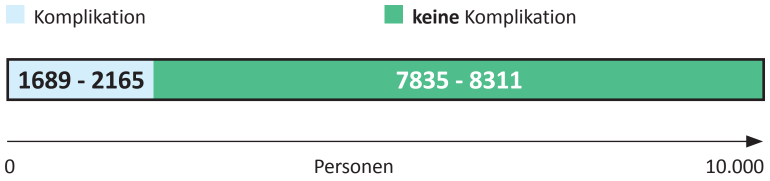

8 RCTs 676

Komplikation	Häufigkeit	Sedierung verglichen mit keiner Sedierung

Eine starke **Verlangsamung des Herzschlags (Bradykardie)** während der Operation kann die ausreichende Versorgung der Organe mit Sauerstoff gefährden und eine Behandlung mit Medikamenten wird notwendig.

keine Unterschiede zwischen den Verfahren

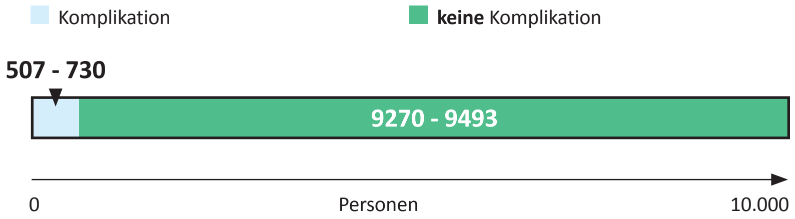

4 RCTs 386

Komplikationen, die den gesamten Körper/Organismus betreffen

Komplikation	Häufigkeit	Sedierung verglichen mit keiner Sedierung

Unwillkürliches Zittern (Kältezittern) kann während und nach der Operation auftreten und unangenehm sein, je nach Schwere aber auch zu weiteren Komplikationen (z.B. Aufreißen von Nähten oder Störungen des Herz-Kreislaufsystems) führen. Je nach Dauer und Schwere des Zitterns können weitere Behandlungen nötig werden.

weniger Zittern mit Sedierung

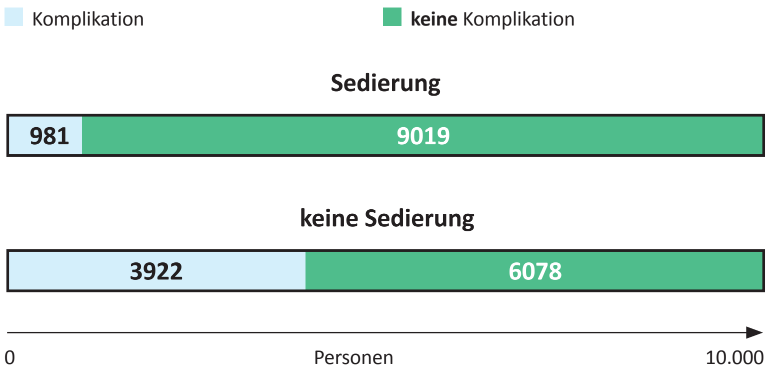

2 RCTs 221

Ein **Schwindelgefühl** kann nach der Operation durch verschiedene Ursachen vorübergehend auftreten. Je nach Ursache und Schwere kann eine weitere Behandlung notwendig werden. Auf Schwindel ist besonders beim Aufstehen und Gehen zu achten.

Diese Komplikation wurde in 1 Studie betrachtet, es sind jedoch keine Fälle aufgetreten. Es kann daher über die Häufigkeit keine Angabe gemacht werden.

1 RCT 120

Eine **zu tiefe Sedierung** kann durch die Gabe von Medikamenten entstehen. Dies kann verschiedene Folgen haben (z.B. Delir).

Diese Komplikation wurde in 2 Studien betrachtet. Die Berechnung der Häufigkeit ist mit erheblichen Unsicherheiten verbunden, wodurch keine Angaben zur Häufigkeit gemacht werden können.

2 RCTs 110

Welche weiteren Komplikationen und unerwünschten Ereignisse können auftreten?

Es ist bekannt, dass die folgenden Ereignisse und Komplikationen in der Vergangenheit aufgetreten sind. Wie häufig diese aber tatsächlich auftreten, kann aus den vorliegenden Studien nicht ermittelt werden.

Ereignisse und Komplikationen in Verbindung mit der Anlage eines Venenzugangs [5]

Blutergüsse
Durch die Anlage eines Venenkatheters können Blutergüsse oder Blutungen hervorgerufen werden.

Infektionen der Einstichstelle
Die Folge von Infektionen an der Einstichstelle können Abszesse (Eiteransammlungen), das Absterben von Gewebe, die Bildung von Narben und Venenentzündungen sein.

Nervenverletzungen
Durch die Anlage eines Venenkatheters kann es zu Nervenverletzungen mit vorübergehenden oder dauerhaften Schäden (z.B. Taubheitsgefühle, Missempfindungen, Störungen der Bewegung, Lähmungen, Schmerzen) kommen.

Schmerzen durch die Anlage des Venenzugangs
Der Venenzugang wird mithilfe einer Nadel in die Vene eingebracht. Dies kann Schmerzen an der Einstichstelle verursachen.

Komplikationen, die unabhängig vom Anästhesieverfahren auftreten können [6]

Allergische Reaktionen und Unverträglichkeiten
Allergische Reaktionen und Unverträglichkeiten auf verabreichte Medikamente können zu leichten Reaktionen (z.B. Übelkeit, Erbrechen oder Ausschlag) führen und lassen sich in der Regel leicht behandeln. Unter Umständen können auch schwere Reaktion auftreten und zu lebensbedrohlichen Beeinträchtigungen führen.

Beschleunigter Herzschlag (Tachykardie)
Durch die Gabe von Medikamenten kann es zu einem beschleunigten Herzschlag kommen, der eine Behandlung erfordern kann.

Erhöhter CO2-Gehalt im Blut (Hyperkapnie/Hyperkarbie)
Ein erhöhter Anteil an Kohlendioxid im Blut führt zu einer Stresssituation des Körpers mit beschleunigtem Herzschlag und Bluthochdruck. Während der CO2-Gehalt zu hoch ist, kann es zu Einschränkungen des Bewusstseins kommen.

Hirnvenenthrombose
Es kann während oder nach der Anästhesie zu einer Hirnvenenthrombose kommen. Dies kann verschiedene Ursachen haben und dauerhafte Schädigungen des Gehirns können die Folge sein.

Juckreiz (Pruritus)
Vorübergehend können Juckreiz und Hautrötungen (auch der Schleimhäute) auftreten. Zur Linderung kann ein Medikament eingesetzt werden.

Lagerungsschäden
Während der Operation können vorübergehende oder dauerhafte Schädigungen der Haut, des Gewebes oder der Nerven auftreten.

Multiples Organversagen
Es kann während einer Anästhesie zum Versagen eines oder mehrerer Organe kommen.

Nebenwirkungen von Medikamenten während der Anästhesie
Jedes Medikament, das während der Anästhesie verabreicht wird, hat seine eigenen Risiken und kann ebenso zu Schäden (z.B. Leberschädigung oder Verminderung weißer Blutkörperchen) führen.

Pneumothorax
Durch eine Verletzung der Haut, die die Lunge umgibt (Pleura), kann es zu einem Pneumothorax, einer Ansammlung von Luft zwischen Pleura und der Lunge selbst kommen.

Rapider Anstieg der Körpertemperatur (Hyperthermie)
Durch Medikamente kann bei manchen Menschen ein lebensbedrohlicher Anstieg der Körpertemperatur ausgelöst werden, der eine intensivmedizinische Behandlung erfordert.

Schmerzen bei der Injektion
Bei manchen Medikamenten kann es während der Verabreichung zu Schmerzen an der Injektionsstelle kommen.

Veränderungen des Blutzuckerspiegels (Hyperglykämie/Hypoglykämie)
Es kann während einer Anästhesie zu einer behandlungsbedürftigen Erhöhung/einem behandlungsbedürftigen Absinken des Blutzuckerspiegels kommen.

Ereignisse und Komplikationen in Verbindung mit einer Vollnarkose [2]

Vorzeitiges Erwachen
Während der Operation kann es passieren, dass Sie wach werden und/oder Schmerzen empfinden. Die Folge können psychische Beschwerden sein.

Ereignisse und Komplikationen in Verbindung mit einer Spinalanästhesie [2]

Blutungen im Gehirn oder Spinalraum
Blut oder Flüssigkeitsansammlungen können lebensbedrohliche und/oder dauerhafte Folgen haben.

Fehlschlag der Spinalanästhesie

Eine Spinalanästhesie kann unter Umständen nicht gelingen, beispielsweise, wenn kein Zugang zum Spinalkanal gelegt werden kann oder die Betäubung der Gliedmaßen nicht ausreichend ist. Wenn dies vor Beginn der Operation geschieht, können Sie sich für eine Vollnarkose entscheiden oder die Operation gegebenenfalls verschieben.

Hirnhautentzündung
Durch eine aufsteigende Infektion kann eine Hirnhautentzündung hervorgerufen werden. Diese kann zu dauerhaften Schäden führen.

Lähmungen
Vorübergehende Lähmungen sind nach einer Spinalanästhesie zu erwarten und verschwinden in der Regel innerhalb weniger Stunden. Es können aber auch dauerhafte Lähmungen auftreten (z. B. Querschnittslähmung).

Postspinale Kopfschmerzen
Durch den Einstich in den Rückenmarkskanal können vorübergehend Kopfschmerzen auftreten, bis sich die Einstichstelle wieder verschlossen hat.

Potenzstörungen
Vorübergehende oder auch dauerhafte Potenzstörungen können infolge einer Spinalanästhesie auftreten.

Rückenschmerzen
Durch die Spinalanästhesie können vorübergehende oder dauerhafte Rückenschmerzen auftreten.

Seh- und Hörstörungen
Durch Reizung von Hirnnerven können vorübergehende oder dauerhafte Seh- und Hörstörungen entstehen.

Verabreichung der Betäubungsmittel in den Blutkreislauf
Wenn das Betäubungsmittel sofort in den Blutkreislauf gelangt oder schneller als gewöhnlich vom Gewebe ins Blut übergeht, können Krampfanfälle, Bewusstlosigkeit und lebensgefährliche Herz-Kreislauf und Atemstörungen auftreten.

Unerwünschte Ereignisse [7]

Die folgenden Ereignisse traten in der Vergangenheit zusammen mit einer Anästhesie auf. Ob diese Ereignisse auf die Anästhesie zurückzuführen sind oder ob diese andere Ursachen hatten, ist unklar.

- Erkrankungen des Magen-Darm-Traktes
- Herzinsuffizienz
- Infektionen des Organraumes
- Psychische Störungen (Stimmungsschwankungen)
- Unwillkürliche Muskelzuckungen (Myoklonien)
- Wasseransammlungen in der Lunge (Lungenödem)

Welche zusätzlichen Maßnahmen sind während der Anästhesie möglich?

Vor oder während der Anästhesie kann es aus verschiedenen Gründen notwendig sein, zuvor geplante Maßnahmen zu ändern oder zu erweitern. Zum Beispiel kann es vorkommen, dass eine geplante Spinalanästhesie nicht durchgeführt werden kann. In dem Fall ist es möglich, sich stattdessen für eine Vollnarkose zu entscheiden. Auch während der Operation kann es vorkommen, dass die Spinalanästhesie in eine Vollnarkose umgewandelt werden muss. Welche Komplikationen mit einer Spinalanästhesie und einer Vollnarkose verbunden sind, können Sie auf den Seiten 7-21 nachlesen.

Weiterhin kann es vorkommen, dass die geplante Form der Beatmung (z.B. Kehlkopfmaske) nicht eingesetzt werden kann. Es wird dann auf eine andere Methode zurückgegriffen. Auf Seite 17 können Sie lesen, mit welcher Häufigkeit das eintreten kann.

Vor der Operation erhalten Sie einen Venenzugang (Venenverweilkanüle). Dieser wird meist an der Hand oder am Unterarm angelegt. Es kann vorkommen, dass während der Operation ein anderer Venenzugang gelegt werden muss. In dem Fall kann es notwendig werden, dass ein zentraler Venenkatheter (ZVK) gelegt wird. Dieser wird in ein zentrales Blutgefäß (Vene) eingelegt. Dies kann zum Beispiel im Bereich des Halses erfolgen. Auf diese Weise können zum Beispiel für den Kreislauf wichtige Medikamente zuverlässig verabreicht werden. Durch die Anlage eines ZVKs können ebenso Komplikationen hervorgerufen werden. Weitere Informationen zu den Komplikationen finden Sie auf den Seiten 20 und 21.

Was gibt es vor der Anästhesie zu beachten? [1]

Ihr Behandlungsteam wird Ihnen weitere Informationen geben, wenn für Sie individuell weitere Dinge zu beachten sind. Informieren Sie Ihr Behandlungsteam, wenn Sie von den folgenden Empfehlungen abgewichen sind.

Bis wann kann ich vor der Anästhesie etwas essen?

Sie können bis 6 Stunden vor der Anästhesie noch eine kleine Mahlzeit zu sich nehmen (z.B. eine Scheibe Brot). Danach sollten Sie nichts Festes mehr zu sich nehmen (auch keinen Kaugummi).

Bis wann kann ich vor der Anästhesie etwas trinken?

Bis zwei Stunden vor der Anästhesie können Sie noch 1-2 Gläser oder Tassen mit klarer Flüssigkeit (Kaffee ohne Milch und Zucker, Wasser oder Tee) zu sich nehmen. Verzichten Sie auf andere Getränke, die Fett oder feste Bestandteile enthalten (z.B. Milch, Orangensaft) und auf Alkohol. Ab 2 Stunden vor der Operation sollten Sie keine Flüssigkeiten mehr zu sich nehmen.

Was gibt es noch zu beachten?

Wenn Sie Medikamente einnehmen, wird Ihr Behandlungsteam mit Ihnen besprechen, welche Medikamente Sie für die Anästhesie absetzen sollten oder welche gegebenenfalls ersetzt werden sollten. Bringen Sie außerdem wichtige Unterlagen und Patientinnen- oder Patientenpass mit, falls vorhanden (z.B. zu Allergien, Anästhesie, Herzschrittmacher oder den Marcumar©-Ausweis). Wenn Sie Dokumente besitzen wie zum Beispiel eine Vorsorgevollmacht, eine Patientinnen- oder Patientenverfügung oder eine Betreuungsverfügung, können Sie diese auch mitbringen und dem Behandlungsteam für die Dauer des Aufenthaltes aushändigen.

Bitte legen Sie alle Gegenstände, die abnehmbar oder herausnehmbar sind vor der Anästhesie ab (z.B. Kontaktlinsen, jeglichen Schmuck oder Piercings, künstliche Haare, herausnehmbare Zahnprothesen…) und verwenden Sie kein Make-up oder Gesichtscreme. Klären Sie mit Ihrem Behandlungsteam, ob sie Hörgerät oder Brille gegebenenfalls bei sich behalten können bis kurz vor der Operation.

Sollte ich vor der Anästhesie aufhören zu rauchen?

Wenn Sie vor dem Operationstermin noch ausreichend Zeit haben, kann es hilfreich sein, vier Wochen oder früher vor der Operation das Rauchen einzustellen. Es gibt Hinweise, dass Sie so das Auftreten von Komplikationen (z.B. bezogen auf die Atemwege) reduzieren können.

Was geschieht nach der Operation? [1]

Nach der Operation werden Sie in einen Aufwachraum oder auf die Intensivstation gebracht, wo Sie und Ihre Körperfunktionen von geschultem Fachpersonal engmaschig überwacht werden. Durch die Anästhesie können Sie verwirrt sein und zum Beispiel versuchen, aufzustehen, obwohl Sie noch nicht komplett wach sind. Es kann dann nötig werden, Ihre Bewegungsfreiheit zu ihrem eigenen Schutz kurzfristig einzuschränken (z.B. durch ein Bettgitter).

Was gibt es nach der Anästhesie zu beachten? [1]

Was muss ich beachten, um Stürze und Verletzungen zu vermeiden?

Durch die Anästhesie und die Operation selbst bedingt, besteht die Gefahr eines Sturzes, falls Ihnen zum Beispiel schwindelig wird, Sie noch nicht ausreichend Kraft in den Beinen oder Schmerzen haben. Wenn Sie eine Spinalanästhesie erhalten haben, können Sie darauf achten, ihre Beine vor Verletzungen zu schützen. Das Gefühl in Ihren Beinen kommt erst nach und nach zurück und Sie spüren nicht sofort, wenn Sie sich stoßen oder auf etwas liegen.

Worauf muss ich bei der Einnahme von Medikamenten oder anderen Präparaten achten?

Nehmen Sie vorerst nur die Medikamente ein, die sie mit dem Behandlungsteam besprochen haben. Sprechen Sie Ihr Behandlungsteam an, wenn Sie sich unsicher sind, wann Sie welche Medikamente nach der Operation wieder einnehmen können. Wenn Sie die Pille zur Verhütung einnehmen, achten Sie darauf, dass die empfängnisverhütende Wirkung nach der Anästhesie zeitweise herabgesetzt ist.

Auf welche Warnzeichen muss ich nach der Operation achten?

Falls Sie eines der folgenden Anzeichen bei sich nach der Operation feststellen, informieren Sie bitte unverzüglich Ihr Behandlungsteam: Bewusstseinsstörungen, Herz-/Kreislauf- oder Atemprobleme, starke Schmerzen, Fieber oder Schüttelfrost, Übelkeit oder Erbrechen, Halsschmerzen oder Heiserkeit, Probleme beim Sprechen oder Schlucken, Entzündungen, Probleme beim Stuhlgang oder beim Wasserlassen, Anzeichen von Lähmungen und/oder Störungen bei der Bewegung, anhaltende Missempfindungen.

Wer hat den Aufklärungsbogen mit welchen Quellen, wie erstellt?

Auf dieser Webseite sind alle verwendeten Quellen sowie alle beteiligten Personen aufgelistet.
Außerdem finden Sie hier den Methodenreport.

https://evab-pilot-ifom.leitlinie-gesundheitsinformation.de/

Wichtige Fragen zu Ihrem Gesundheitszustand [1]

Das Risiko ärztlicher Eingriffe kann durch Ihre körperliche Verfassung und Vorschäden beeinflusst werden. Wir bitten Sie daher, folgende Fragen zu beantworten. Anhand dieser Informationen kann das Behandlungsteam Risiken in Ihrem speziellen Fall besser abschätzen und ggf. Maßnahmen ergreifen, um Komplikationen zu vermeiden.

Alter (Jahre):	Größe (cm):	Gewicht (kg):
Geschlecht: ☐ weiblich	☐ männlich	☐ divers
Könnten Sie schwanger sein?	☐ ja	☐ nein
Stillen Sie?	☐ ja	☐ nein

1. Beruf

____

2. Erfolgte bei Ihnen in der letzten Woche eine **andere ärztliche Behandlung**? ☐ ja ☐ nein

Wenn ja, welche?

____

____

3. Hatten Sie in den letzten 4 Wochen einen **Infekt** (z.B. Atemwege, Magen-Darm, Harnwege)? ☐ ja ☐ nein

Wenn ja, welchen?

____

4. Besteht oder bestand bei Ihnen eine **Infektionskrankheit** (z.B. Hepatitis, HIV/AIDS, Hirnhautentzündung, Tuberkulose)? ☐ ja ☐ nein

Wenn ja, welche?

____

5. Nehmen Sie **Medikamente** ein (z.B. gerinnungshemmende Mittel wie Marcumar® oder Aspirin ®, Schmerzmittel, insbesondere metforminhaltige Antidiabetika, Herz-/Kreislauf-Medikamente, Hormonpräparate, Schlaf- oder Beruhigungsmittel, blutdrucksenkende Mittel)? ☐ ja ☐ nein

Wenn ja, welche?

____

____

6. Besteht eine **Allergie oder Überempfindlichkeit** gegenüber Medikamenten (z.B. Antibiotika, Novalgin, Paracetamol), Betäubungsmittel, Röntgenkontrastmittel, Latex, Desinfektionsmittel, Jod, Pflaster oder Kunststoffe? ☐ ja ☐ nein

Wenn ja, welche?

____

____

7. Wurde bei Ihnen schon einmal eine **Operation** durchgeführt? ☐ ja ☐ nein

Wenn ja, welche?

____

____

Wenn ja, traten Komplikationen auf? ☐ ja ☐ nein

Wenn ja, welche?

____

____

8. Wurde bei Ihnen schon einmal eine **Anästhesie** (z.B. Vollnarkose, Spinalanästhesie, Regionalanästhesie, örtliche Betäubung, Sedierung) durchgeführt (z.B. beim Zahnarzt)? ☐ ja ☐ nein

Wenn ja, welche?

____

____

Wenn ja, traten Komplikationen auf? ☐ ja ☐ nein

Wenn ja, welche?

____

____

9. Neigen Sie zu **Übelkeit oder Erbrechen**? ☐ ja ☐ nein

10. Besteht bei Ihnen eine Veranlagung zu **hohem Fieber** bei oder nach einer Anästhesie (maligne Hyperthermie)? ☐ ja ☐ nein

11. Trat bei Blutsverwandten bei oder nach einer Anästhesie eine maligne Hyperthermie auf? ☐ ja ☐ nein

12. Ist bei Ihnen schon einmal eine Übertragung von **Blut oder Blutbestandteilen** (Transfusion) erfolgt? ☐ ja ☐ nein

13. Besteht bei Ihnen eine **erhöhte Blutungsneigung**? Haben Sie z.B. häufig Nasen- oder Zahnfleischbluten, blaue Flecken oder nach einer Operation nachgeblutet? ☐ ja ☐ nein

14. Besteht oder bestand bei Ihnen eine **Gefäßerkrankung** (z.B. Arteriosklerose, Krampfadern, Erkrankung der Herzkranzgefäße, Durchblutungsstörung, Aneurysma, Verengung der Halsschlagader)? ☐ ja ☐ nein

Wenn ja, welche?

____

____

15. Kam es bei Ihnen schon einmal zu einem **Gefäßverschluss** durch Blutgerinnsel **(Thrombose / Embolie)**? ☐ ja ☐ nein

16. Besteht oder bestand bei Ihnen eine **Herz-Kreislauf-Erkrankung** (z.B. koronare Herzkrankheit, Bluthochdruck, Rhythmusstörungen, Schlaganfall, Herzinfarkt, Angina pectoris, Herzmuskelentzündung, Klappenfehler)? ☐ ja ☐ nein

Wenn ja, welche?

____

____

17. Haben Sie **Atemnot** beim Treppensteigen? ☐ ja ☐ nein

Wenn ja, nach wie vielen Treppenstufen müssen Sie eine Pause einlegen?

____

18. Besteht oder bestand bei Ihnen eine **Atemwegs- oder Lungenerkrankung** (z.B. chronische Bronchitis, Lungenentzündung, Asthma bronchiale, Lungenblähung, angeborene Fehlbildung) ☐ ja ☐ nein

Wenn ja, welche?

____

____

19. Haben Sie nächtliche **Atemstörungen** (z.B. starkes Schnarchen, Atempausen [Schlafapnoe])? ☐ ja ☐ nein

Wenn ja, welche?

____

____

20. Besteht bei Ihnen eine **Stimmbandlähmung**? ☐ ja ☐ nein

21. Besteht bei Ihnen eine **Zwerchfelllähmung**? ☐ ja ☐ nein

22. Besteht oder bestand bei Ihnen eine **Erkrankung des Verdauungssystems** (z.B. Speiseröhre, Magen, Darm)? ☐ ja ☐ nein

Wenn ja, welche?

____

____

23. Tritt bei Ihnen **Sodbrennen** auf? ☐ ja ☐ nein

24. Besteht bei Ihnen eine **Refluxkrankheit**? ☐ ja ☐ nein

25. Bestand bei Ihnen eine **Erkrankung der Oberbauchorgane** (z.B. Leberentzündung/Hepatitis, Fettleber, Zirrhose, Gallenkoliken/-steine, Gelbsucht, Entzündung der Bauchspeicheldrüse/Pankreatitis)? ☐ ja ☐ nein

Wenn ja, welche?

____

____

26. Besteht oder bestand bei Ihnen eine Erkrankung der **Nieren oder Harnorgane** (z.B. Nierenfunktionsstörung, Nierensteine, chronischer Harnwegsinfekt, Nierenentzündung, angeborene Fehlbildung wie bspw. eine Doppelniere, Blasenentleerungsstörung)? ☐ ja ☐ nein

Wenn ja, welche?

____

____

27. Besteht bei Ihnen eine **Stoffwechselerkrankung** (z.B. Zuckerkrankheit, Gicht)? ☐ ja ☐ nein

Wenn ja, welche?

____

____

28. Besteht oder bestand bei Ihnen eine **Schilddrüsenerkrankung** (z.B. Überfunktion, Unterfunktion, Kropf, Morbus Hashimoto)? ☐ ja ☐ nein

Wenn ja, welche?

____

____

29. Besteht oder bestand bei Ihnen eine **Muskel- oder Skeletterkrankung** (z.B. Muskelschwäche, Gelenkerkrankung, Osteoporose, Osteomalazie)? ☐ ja ☐ nein

Wenn ja, welche?

____

____

30. Besteht oder bestand bei Ihnen eine **Erkrankung des Nervensystems** (z.B. Gehstörungen, Lähmungen, Krampfleiden (Epilepsie), Morbus Parkinson, Gefühlsstörungen, Polyneuropathie, Schmerzen)? ☐ ja ☐ nein

Wenn ja, welche?

____

____

31. Besteht bei Ihnen eine **Augenerkrankung** (z.B. Grauer Star, Grüner Star)? ☐ ja ☐ nein

Wenn ja, welche?

____

____

32. Bestehen bei Ihnen **weitere Erkrankungen/Beeinträchtigungen** (z.B. Wirbelsäulenschäden, Schulter-Arm-Syndrom, Multiple Sklerose, Restless-Legs-Syndrom, häufige Kopfschmerzen, Depressionen, Hörschwäche)? ☐ ja ☐ nein

Wenn ja, welche?

____

____

33. Gibt es Besonderheiten beim **Zustand Ihrer Zähne** (z.B. lockere Zähne, Zahnspange, Prothese, Brücke, Krone, Implantat, Retainer, Paradontose)? ☐ ja ☐ nein

Wenn ja, welche?

---

34. Haben Sie **Implantate** im Körper (z.B. Schrittmacher, Defibrillator, Herzklappe, Stent, künstliches Gelenk, Silikon, Hydrogel, Zähne, Metall)? ☐ ja ☐ nein

Wenn ja, welche?

---

35. **Rauchen** Sie? ☐ ja ☐ nein

36. Trinken Sie mehrmals pro Woche **Alkohol** (z.B. Bier, Wein, hochprozentige Alkoholika)? ☐ ja ☐ nein

Wenn ja, welchen?

---

37. Nehmen Sie **Drogen**? ☐ ja ☐ nein

38. Liegen **Vorsorgeregelungen** vor (z.B. Patientenverfügung, Betreuungsverfügung, Vorsorgevollmacht)? ☐ ja ☐ nein

Wenn ja, welche?

Anmerkungen zum Aufklärungsgespräch *(durch das Behandlungsteam auszufüllen)*

(z.B. individuelle Risiken und mögliche Komplikationen, Fragen der Patientin/des Patienten, mögliche Folge- oder Nebeneingriffe, Einschränkungen im Umfang der Einwilligung, gesetzliche Vertretung der Patientin/des Patienten)

Anmerkungen zum Zahnstatus der Patientin / des Patienten

geplantes Betäubungsverfahren:

- ☐ Vollnarkose und Beatmung mittels
  - ☐ einer Kehlkopfmaske (Larynxmaske)
  - ☐ eines Beatmungsschlauchs (Tubus)
- ☐ Spinalanästhesie
  - ☐ mit Beruhigungsmittel (Sedierung)
  - ☐ ohne Beruhigungsmittel (Sedierung)
- ☐ sonstiges:

Ihre Fragen/Notizen

Einwilligung

Mit der Einwilligung bestätigen Sie, dass Sie die für Ihre Entscheidung notwendigen Informationen zum geplanten Anästhesieverfahren, einschließlich der Art und Bedeutung, Behandlungsalternativen, Risiken, mögliche Komplikationen, mögliche Neben- oder Folgeeingriffe und mögliche Abweichungen vom geplanten Verfahren in einer für Sie verständlichen Art und Weise erhalten haben.

Sie bestätigen, dass alle für Sie wichtigen Fragen verständlich geklärt werden konnten und keine weiteren Fragen bestehen. Sie bestätigen, dass Sie mit medizinisch erforderlichen Änderungen und Erweiterungen des Anästhesieverfahrens sowie Neben- oder Folgeeingriffen, die direkt während der geplanten Operation stattfinden müssen, ebenso einverstanden sind.

☐ Ich willige in das vorgesehene Anästhesieverfahren ein.

Ort, Datum , Uhrzeit

Patientin/Patient oder gesetzliche(r) Vertreter(in)

Ärztin/Arzt

☐ Ich habe eine Kopie des Aufklärungsbogens erhalten

☐ Ich möchte keine Kopie des Aufklärungsbogens erhalten.

Ort, Datum , Uhrzeit

Patientin/Patient oder gesetzliche(r) Vertreter(in)

Aktualität: 2021
Nächste geplante Aktualisierung: 2024

Impressum

Erstellung durch das EvAb-Pilot-Projektteam
Kontakt:
Prof. Dr. phil. Anke Steckelberg
Institut für Gesundheits- und Pflegewissenschaft
Martin-Luther-Universität Halle-Wittenberg
Anke.Steckelberg@medizin.uni-halle.de

Das Projekt, in dem dieses Informationsmaterial entstanden ist, wurde mit Mitteln des Innovationsausschusses beim Gemeinsamen Bundesausschuss unter dem Förderkennzeichen 01VSF19025 gefördert.

Illustration und Layout durch Martin Siegmund (Siegmund und Fischer Grafik)
