# Supplementary material for: Evidence-based informed consent forms for total knee arthroplasty and anaesthesia: development and pilot study
Source: J Orthop Surg Res. 2026 Feb 5;21:156. doi: 10.1186/s13018-026-06729-z (PMC12930710; doi:10.1186/s13018-026-06729-z)
Supplement: Supplementary file 5 — Supplementary Material 5 [file 13018_2026_6729_MOESM5_ESM.pdf]

# Additional file 5: Knowledge test to assess risk perception and knowledge of TKA and anaesthesia

## Questions of knowledge about a Total Knee Replacement Operation

* = Adjustments in the analysis

### 1st Part: Total Knee Endoprosthesis/Replacement

You have been diagnosed with osteoarthritis in your knee. You are thinking about having an operation in which your knee would be replaced with an artificial prosthesis. This can be done with a so-called Total Knee Replacement (Knee-TEP), in which both the cartilage and the bone ends adjacent to the knee joint are replaced. Under certain conditions, a partial knee prosthesis is also possible. In this operation, only a part of the knee joint is replaced. In addition, it is also possible to treat the complaint using conservative methods (e.g. physiotherapy, shoe insoles, weight reduction).

These procedures can help you but complications can also occur.

1. **What improvement can you expect following a knee TEP?**

There are several correct answers here. Please tick **all the correct** answers.

a. Functionality can **be fully restored** with a knee TEP.
b. **Pain can be reduced** through a knee TEP. (correct)
c. A knee TEP can **improve physical resilience**. (correct)
d. **No benefit** can be expected from a knee TEP

2. **Which of the following complications do you think can occur as a result of a knee TEP?**

There are several correct answers here. Please tick **all the correct** answers.

a. **Restriction of the ability to bend** the operated knee. (correct)
b. **Restriction of the ability to stretch** the operated knee (correct)
c. **Loosening of the inserted artificial joint.** (correct)
d. **Development of rheumatoid arthritis** in the operated knee**.**

3. **Out of 1000 people who have had a knee TEP, in how many cases will the knee TEP have to be replaced on the same knee within 10 years?** *

* The question could not be answered based on the completed materials and was therefore removed from the analysis.

**Which statement is correct?** (One answer option is correct.)

a. By approx. 250 people
b. By less than 150 people
c. By less than 10 people
d. By less than 1 person

4. **Under certain conditions, a partial knee replacement may also be considered instead of a total endoprosthesis (TEP)**

**Which statement is correct?** (One answer option is correct.)

a. The durability of a total knee replacement is slightly **lower** after 5 years than that of a partial knee replacement.
b. The durability of a total knee replacement is slightly **higher** after 5 years than that of a partial knee replacement.
c. Die The durability of a total knee replacement and a partial knee replacement after 5 years is **about the same**. (correct)
d. The durability of total knee replacements and partial knee replacements after 5 years has **not yet been sufficiently investigated**.

## 5. Comparison of conservative and operative procedures

In a study, people were randomly divided into two groups. The people in the first group had a knee TEP followed by conservative treatments. The second group received conservative treatments only. The conservative treatments included among others nutritional advice and physiotherapy. In order to compare the knee TEP with the mere conservative treatments, questionnaires were filled out by the patients before and after the operation resp. after the treatment programme. Among other things, questions were asked about pain (e.g. when resting at night or when climbing stairs.) and about restrictions in everyday life (e.g. when shopping or doing housework). The patients had five possible answers for each question.

**For example:**

***How intense was your pain in the past week when you had to go up or down stairs?***

| None | Slightly | Average | Bad | Very bad |
|---|---|---|---|---|
| ☐ | ☐ | x | ☐ | ☐ |
| 4 points | 3 points | 2 points | 1 point | 0 points |

A total value between 0 and 100 was calculated from all the responses. The **value 100** expressed **no pain** or **no restrictions**. **However, 0** stands for the **worst pain** or the **most severe restrictions** in everyday life.

**5.1** In This study has shown that the **pain intensity** score for people who had received only **conservative treatment** had **improved** in one year from **50 points to around 70 points**.

Estimate how the score for **pain intensity** has changed for people who received a **knee TEP**. Select the correct option from the possible answers to complete the following sentence:

For people who had received a **knee TEP** the score for the pain intensity had improved in one year from **50 to around _____ points**.

☐ 78 ☐ 67 ☐ 53 ☐ 86 (correct*)

* A value of 84 is shown in the finalised materials. For the descriptive evaluation, a value of 86 is considered correct.

**5.2** The study has also shown that the score for **restrictions in everyday life** for people who had received only a **conservative treatment** had improved in one year from **54 to around 71**.

Estimate how the score for **restrictions in everyday life** has changed for people who received a **knee TEP**. Select the correct option from the possible answers to complete the following sentence:

For people who had received a **knee TEP** the score for the perceived **restrictions in everyday life** had **improved** in one year from **54 to around ____.**

☐ 56 ☐ 84 (correct) ☐ 99 ☐ 72

**6. In a study, the occurrence of complications was examined both for people, who had received only a conservative treatment programme, and for people who had received a knee TEP operation.** *

* The materials used in the clinic do not contain enough information to answer the question correctly. The question was therefore removed from the analysis.

Both **conservative treatment methods** and **surgical treatment methods** can lead to **serious complications**. Serious complications include thrombosis in the leg.

**6.1**
Estimate how many out of 1000 people, who had received a **conservative treatment programme**, suffered from a thrombosis in the leg within one year?

**_____ out of 1000 people**

**6.2**
Estimate how many out of 1000 people, who had had a **knee TEP**, suffered from a thrombosis in the leg within one year?

**_____ out of 1000 people**

## 2nd Part: Anaesthetic

An anaesthetic procedure is used to ensure that you do not feel any pain during an operation. A general anaesthetic or a spinal anaesthetic can be used for a knee TEP. With general anaesthesia, consciousness and the sensation of pain are suppressed throughout the body. With spinal anaesthesia, only the lower part of the body is anaesthetised. For this purpose, the anaesthetic is injected at the level of the lumbar spine into the so-called spinal space, which is filled with cerebrospinal fluid.

Side effects can occur with both methods.

1. **Imagine that your knee TEP is performed under general anaesthesia. Which of the following side effects do you think may occur?**

   There are several correct answers here. Please tick **all the correct** answers.

   a. Bruising (correct)
   b. Severe allergic reactions (correct)
   c. Pneumonia (correct)
   d. Backache
   e. Cramp-like blockage of the respiratory tract (correct)
   f. Swallowing difficulties and hoarseness (correct)

2. **Following a knee TEP operation with anaesthetic, confusion and impaired mental capacity may occur.  Which of the following statements are correct?**

   There are several correct answers here. Please tick **all the correct** answers.

   a. After an operation, confusion occurs more frequently with older people than with younger people. (correct)
   b. Impaired mental capacity may be permanent. (correct)
   c. A previous stroke can increase the risk of becoming confused after an operation. (correct)
   d. Confusion (delirium) after a general anaesthetic occurs less frequently than after a spinal anaesthetic.

3. **After a knee TEP under a general anaesthetic, nausea with or without vomiting may occur.** *

   * In the materials used, the frequency data were presented with a reference group of 10,000 people. The correct answer was adjusted accordingly for the descriptive analysis.

   Please estimate how many out of 1000 people experience nausea with or without vomiting after an anaesthetic:

_______ **out of 1000 people** (500-550*)

* All answers within the specified range were counted as correct.

**4. Following a spinal anaesthetic, headaches can occur and continue for several days.***

* In the materials used, the frequency data were presented with a reference group of 10,000 people. The correct answer was adjusted accordingly for the descriptive analysis.

Please estimate how many out of 1000 people experience headaches after a spinal anaesthetic:

**_______ out of 1000 people** (30-45*)

* All answers within the specified range were counted as correct.
